# An atlas of healthy and injured cell states and niches in the human kidney

**DOI:** 10.1038/s41586-023-05769-3

**Published:** 2023-07-19

**Authors:** Blue B. Lake, Rajasree Menon, Seth Winfree, Qiwen Hu, Ricardo Melo Ferreira, Kian Kalhor, Daria Barwinska, Edgar A. Otto, Michael Ferkowicz, Dinh Diep, Nongluk Plongthongkum, Amanda Knoten, Sarah Urata, Laura H. Mariani, Abhijit S. Naik, Sean Eddy, Bo Zhang, Yan Wu, Diane Salamon, James C. Williams, Xin Wang, Karol S. Balderrama, Paul J. Hoover, Evan Murray, Jamie L. Marshall, Teia Noel, Anitha Vijayan, Austin Hartman, Fei Chen, Sushrut S. Waikar, Sylvia E. Rosas, Francis P. Wilson, Paul M. Palevsky, Krzysztof Kiryluk, John R. Sedor, Robert D. Toto, Chirag R. Parikh, Eric H. Kim, Rahul Satija, Anna Greka, Evan Z. Macosko, Peter V. Kharchenko, Joseph P. Gaut, Jeffrey B. Hodgin, KPMP Consortium, Richard Knight, Stewart H. Lecker, Isaac Stillman, Afolarin A. Amodu, Titlayo Ilori, Shana Maikhor, Insa Schmidt, Gearoid M. McMahon, Astrid Weins, Nir Hacohen, Lakeshia Bush, Agustin Gonzalez-Vicente, Jonathan Taliercio, John O’toole, Emilio Poggio, Leslie Cooperman, Stacey Jolly, Leal Herlitz, Jane Nguyen, Ellen Palmer, Dianna Sendrey, Kassandra Spates-Harden, Paul Appelbaum, Jonathan M. Barasch, Andrew S. Bomback, Vivette D. D’Agati, Karla Mehl, Pietro A. Canetta, Ning Shang, Olivia Balderes, Satoru Kudose, Laura Barisoni, Theodore Alexandrov, Yinghua Cheng, Kenneth W. Dunn, Katherine J. Kelly, Timothy A. Sutton, Yumeng Wen, Celia P. Corona-Villalobos, Steven Menez, Avi Rosenberg, Mohammed Atta, Camille Johansen, Jennifer Sun, Neil Roy, Mark Williams, Evren U. Azeloglu, Cijang He, Ravi Iyengar, Jens Hansen, Yuguang Xiong, Brad Rovin, Samir Parikh, Sethu M. Madhavan, Christopher R. Anderton, Ljiljana Pasa-Tolic, Dusan Velickovic, Olga Troyanskaya, Rachel Sealfon, Katherine R. Tuttle, Zoltan G. Laszik, Garry Nolan, Minnie Sarwal, Kavya Anjani, Tara Sigdel, Heather Ascani, Ulysses G. J. Balis, Chrysta Lienczewski, Becky Steck, Yougqun He, Jennifer Schaub, Victoria M. Blanc, Raghavan Murugan, Parmjeet Randhawa, Matthew Rosengart, Mitchell Tublin, Tina Vita, John A. Kellum, Daniel E. Hall, Michele M. Elder, James Winters, Matthew Gilliam, Charles E. Alpers, Kristina N. Blank, Jonas Carson, Ian H. De Boer, Ashveena L. Dighe, Jonathan Himmelfarb, Sean D. Mooney, Stuart Shankland, Kayleen Williams, Christopher Park, Frederick Dowd, Robyn L. McClelland, Stephen Daniel, Andrew N. Hoofnagle, Adam Wilcox, Shweta Bansal, Kumar Sharma, Manjeri Venkatachalam, Guanshi Zhang, Annapurna Pamreddy, Vijaykumar R. Kakade, Dennis Moledina, Melissa M. Shaw, Ugochukwu Ugwuowo, Tanima Arora, Joseph Ardayfio, Jack Bebiak, Keith Brown, Catherine E. Campbell, John Saul, Anna Shpigel, Christy Stutzke, Robert Koewler, Taneisha Campbell, Lynda Hayashi, Nichole Jefferson, Roy Pinkeney, Glenda V. Roberts, Michael T. Eadon, Pierre C. Dagher, Tarek M. El-Achkar, Kun Zhang, Matthias Kretzler, Sanjay Jain

**Affiliations:** 1 Department of Bioengineering, University of California, San Diego, La Jolla, CA USA; 2 Department of Computational Medicine and Bioinformatics, University of Michigan, Ann Arbor, MI USA; 3 Department of Pathology and Microbiology, University of Nebraska Medical Center, Omaha, NE USA; 4 Department of Biomedical Informatics, Harvard Medical School, Boston, MA USA; 5 Department of Medicine, Indiana University School of Medicine, Indianapolis, IN USA; 6 Department of Internal Medicine, Division of Nephrology, University of Michigan, Ann Arbor, MI USA; 7 Department of Medicine, Washington University School of Medicine, St Louis, MO USA; 8 Broad Institute of Harvard and MIT, Cambridge, MA USA; 9 New York Genome Center, New York, NY USA; 10 Section of Nephrology, Boston University School of Medicine and Boston Medical Center, Boston, MA USA; 11 Kidney and Hypertension Unit, Joslin Diabetes Center, Boston, MA USA; 12 Harvard Medical School, Boston, MA USA; 13 Department of Medicine, Yale University School of Medicine, New Haven, CT USA; 14 Department of Medicine, University of Pittsburgh School of Medicine, Pittsburgh, PA USA; 15 Department of Medicine, Columbia University, New York, NY USA; 16 Lerner Research and Glickman Urology and Kidney Institutes, Cleveland Clinic, Cleveland, OH USA; 17 Department of Internal Medicine, UT Southwestern Medical Center, Dallas, TX USA; 18 Division of Nephrology, Johns Hopkins School of Medicine, Baltimore, MD USA; 19 Department of Surgery, Washington University School of Medicine, St Louis, MO USA; 20 Department of Pathology and Immunology, Washington University School of Medicine, St Louis, MO USA; 21 Department of Pathology, University of Michigan, Ann Arbor, MI USA; 22 American Association of Kidney Patients, Tampa, FL USA; 23 Beth Israel Deaconess Medical Center, Boston, MA USA; 24 Brigham and Women’s Hospital, Boston, MA USA; 25 Duke University, Durham, NC USA; 26 Structural and Computational Biology Unit, European Molecular Biology Laboratory, Heidelberg, Germany; 27 Department of Pathology, Johns Hopkins School of Medicine, Baltimore, MD USA; 28 Icahn School of Medicine at Mount Sinai, New York, NY USA; 29 Ohio State University, Columbus, OH USA; 30 Pacific Northwest National Laboratories, Richland, WA USA; 31 Princeton University, Princeton, NJ USA; 32 Providence Health, Spokane, WA USA; 33 University of California, San Francisco, CA USA; 34 Stanford University, Stanford, CA USA; 35 University of Michigan, Ann Arbor, MI USA; 36 University of Pittsburgh, Pittsburgh, PA USA; 37 University of Washington, Seattle, WA USA; 38 UT Health San Antonio, San Antonio, TX USA; 39 Yale University, New Haven, CT USA; 40 Present Address: San Diego Institute of Science, Altos Labs, San Diego, CA USA

**Keywords:** Cellular signalling networks, Translational research

## Abstract

Understanding kidney disease relies on defining the complexity of cell types and states, their associated molecular profiles and interactions within tissue neighbourhoods^1^. Here we applied multiple single-cell and single-nucleus assays (>400,000 nuclei or cells) and spatial imaging technologies to a broad spectrum of healthy reference kidneys (45 donors) and diseased kidneys (48 patients). This has provided a high-resolution cellular atlas of 51 main cell types, which include rare and previously undescribed cell populations. The multi-omic approach provides detailed transcriptomic profiles, regulatory factors and spatial localizations spanning the entire kidney. We also define 28 cellular states across nephron segments and interstitium that were altered in kidney injury, encompassing cycling, adaptive (successful or maladaptive repair), transitioning and degenerative states. Molecular signatures permitted the localization of these states within injury neighbourhoods using spatial transcriptomics, while large-scale 3D imaging analysis (around 1.2 million neighbourhoods) provided corresponding linkages to active immune responses. These analyses defined biological pathways that are relevant to injury time-course and niches, including signatures underlying epithelial repair that predicted maladaptive states associated with a decline in kidney function. This integrated multimodal spatial cell atlas of healthy and diseased human kidneys represents a comprehensive benchmark of cellular states, neighbourhoods, outcome-associated signatures and publicly available interactive visualizations.

## Abstract (web summary)

A high-resolution kidney cellular atlas of 51 main cell types, including rare and previously undescribed cell populations, represents a comprehensive benchmark of cellular states, neighbourhoods, outcome-associated signatures and publicly available interactive visualizations.

## Main

The human kidneys have vital systemic roles in the preservation of body fluid homeostasis, metabolic waste product removal and blood pressure maintenance. After injury, dynamic acute and chronic changes occur in the renal tubules and surrounding interstitial niche. The balance between successful or maladaptive repair processes may ultimately contribute to the progressive decline in kidney function^2–5^. Defining the underlying molecular diversity at a single-cell level is key to understanding progression of acute kidney injury (AKI) to chronic kidney disease (CKD), kidney failure, heart disease or death—issues that remain a global concern^6,7^.

We report a multimodal single-cell and spatial atlas with integrated transcriptomic, epigenomic and imaging data over three major consortia: the Human Biomolecular Atlas Program (HuBMAP)^8^, the Kidney Precision Medicine Project (KPMP)^9^ and the Human Cell Atlas (HCA)^10^. To ensure robust cell state profiles, healthy reference tissues were obtained from multiple sources, and biopsies were collected from patients with AKI and CKD under rigorous quality assurance and control procedures^8,9,11^. We define niches for healthy and altered states across different regions of the human kidney spanning the cortex to the papillary tip, and identify gene expression and regulatory modules in altered states associated with worsening kidney function. The resultant atlas greatly expands on existing efforts^12–15^ and will serve as an important resource for investigators and clinicians working towards a better understanding of kidney pathophysiology.

## Constructing a kidney cellular atlas

**Fig. 1:**
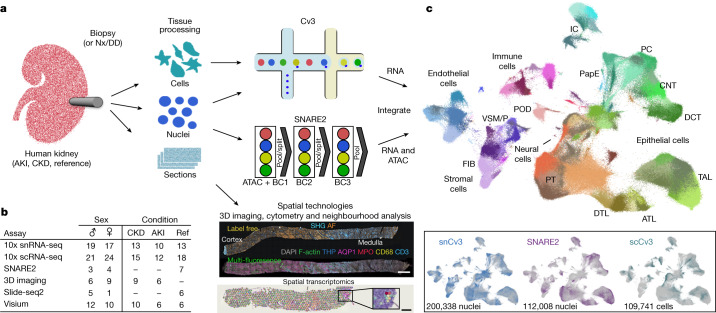
Overview of the technologies used to generate a human kidney cell atlas. **a**, Human kidney samplesconsisted of healthy reference, AKI or CKD nephrectomies (Nx), deceased donors (DD) or biopsies. Tissues were processed for one or more assays, including snCv3, scCv3, SNARE2, 3D imaging or spatial transcriptomics (Slide-seq2, Visium). Scale bars, 1 mm (top) and 300 µm (bottom). **b**, Summary of the samples. Ref, reference. **c**, Omic RNA data were integrated, as shown by joint UMAP embedding, for alignment of cell type annotations across the three different data modalities. IC, intercalated cells; PC, principal cells; VSM/P, vascular smooth muscle cell or pericyte.

**Fig. 2:**
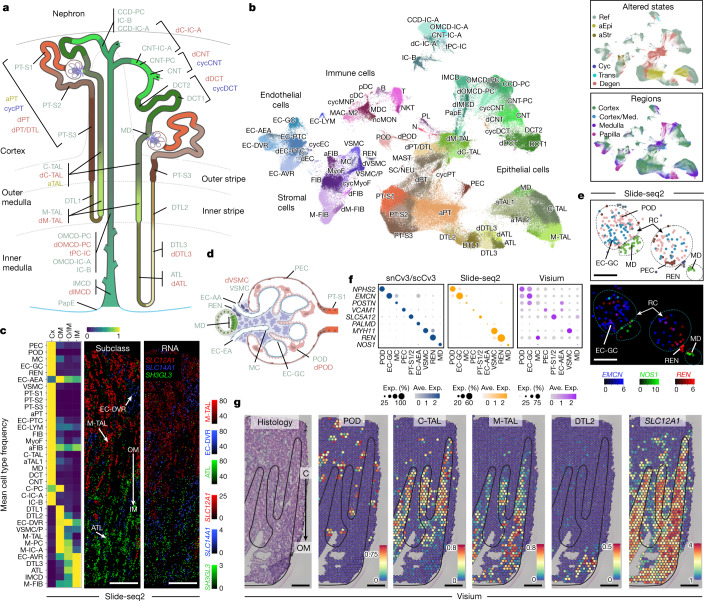
Spatially resolved atlas of molecular cell types. **a**, Schematic of the human nephron showing cell types and states. **b**, UMAP embedding showing cell types (subclass level 3) for snCv3. Insets: overlays for both regional origin and altered-state status. Cyc, cycling; degen, degenerative; trans, transitioning. See Supplementary Table 4 for cell type definitions. **c**, Heat map of Slide-seq cell type frequencies along the corticomedullary axis (three individuals) (left). Middle, representative tissue puck region showing the transition of ATL to M-TAL segments. Right, corresponding expression of marker genes (scaled). Scale bar, 300 µm. **d**, Schematic of the renal corpuscle showing resolved cell types. **e**, The Slide-seq puck area indicated in Extended Data Fig. 4c and predicted cell types for renal corpuscles (top). Bottom, mapped expression values for corresponding marker genes (scaled). Scale bar, 100 µm. **f**, The average expression values for renal corpuscle cell types for markers shown in **e** and Extended Data Fig. 4f for all datasets. Ave., average; Exp., expression. **g**, Visium data on a healthy reference kidney (cortex, top; medulla, bottom). Left, haematoxylin and eosin (H&E)-stained tissue. Right, the per-bead predicted transfer scores for cell types or transcript expression values. Scale bar, 300 µm. Cx, cortex; OM, outer medulla; IM, inner medulla. The black lines outline histologically confirmed medullary rays leading into medulla. Source Data

**Extended Data Fig. 1:**
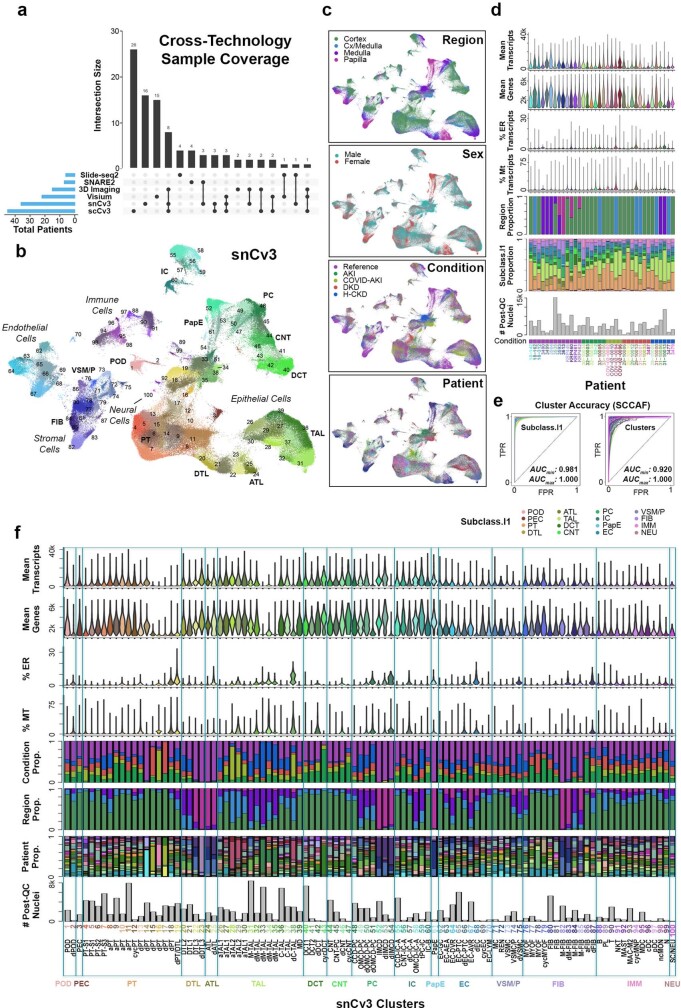
snCv3 cell types and quality metrics. **a**. Number of samples processed across technologies assessed both individually and in combination. **b**. UMAP plots for snCv3 clusters. **c**. UMAP plots as in (**b**) showing the corresponding tissue regions, sex, patient identities and conditions. **d**. Bar and violin plots for snCv3 patients shown in (**c**). Barplots showing the total number of post-QC nuclei used in the snCv3 clustering analysis, and the proportions that were associated with level 1 subclasses, regions sampled or the health or disease conditions. Violin plots show the percentage of transcripts associated with the mitochondria (Mt) or endoplasmic reticulum (ER), as well as mean genes and mean transcripts detected per patient sample. **e**. Receiver operating characteristic (ROC) curve showing snCv3 clustering quality as assessed by the descrimination between subclasses (level 1) or clusters (**b**) using the Single Cell Clustering Assessment Framework (SCCAF). **f**. Bar and violin plots as in (**d**) for snCv3 clusters shown in (**b**), including proportion of nuclei contributed by each patient. Source Data

**Extended Data Fig. 2:**
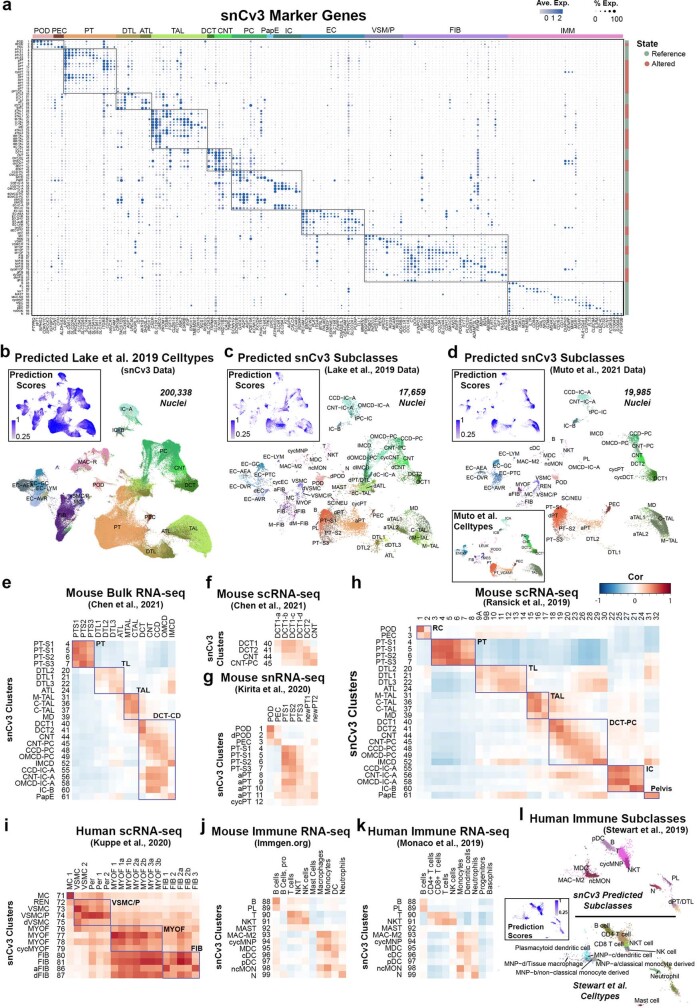
snCv3 marker genes and comparison with reference data. **a**. Dot plot showing averaged marker gene expression values (log scale) and proportion expressed for snCv3 clusters. **b**. Cell type labels predicted from Lake et. al. 2019^12^ mapped on the snCv3 UMAP embedding. Inset shows the corresponding prediction score values. **c**. UMAP of Lake et. al. 2019^12^ data mapped to snCv3 embeddings showing subclass level 3 predicted labels. Inset shows the corresponding prediction score values. **d**. UMAP of Muto et al. 2021^15^ data mapped to snCv3 embeddings showing subclass level 3 predicted labels. Inset shows the corresponding prediction score values. **e**. Heatmap showing correlation of averaged scaled gene expression values for snCv3 epithelial (reference state) clusters and mouse bulk segmental RNA-seq data from Chen et al., 2021^61^. **f**. Heatmap showing correlation of averaged scaled gene expression values for snCv3 distal tubule clusters (reference states) and mouse scRNA-seq data from Chen et al., 2021^61^. **g**. Heatmap showing correlation of averaged scaled gene expression values for snCv3 clusters (reference and altered/adaptive states) and mouse snRNA-seq clusters from Kirita et al., 2020^4^. **h**. Heatmap showing correlation of averaged scaled gene expression values (reference states) for snCv3 clusters and mouse scRNA-seq clusters from Ransick et al., 2019^59^. **i**. Heatmap showing correlation of averaged scaled gene expression values for snCv3 stromal clusters (reference and altered/adaptive states) against human scRNA-seq clusters from Kuppe et al., 2020^25^. **j**. Heatmap showing correlation of averaged scaled gene expression values for snCv3 immune cell clusters and mouse immune cell types from Immgen.org. **k**. Heatmap showing correlation of averaged scaled gene expression values for snCv3 immune cell clusters and human immune cell types from Monaco et al. 2019^62^. **l**. UMAP of Stewart et al., 2019^14^ immune single-cell RNA-seq data mapped to snCv3 embeddings showing subclass level 3 predicted labels (top) and the prior published cell type annotations (bottom). Inset shows the corresponding prediction score values. Source Data

**Extended Data Fig. 3:**
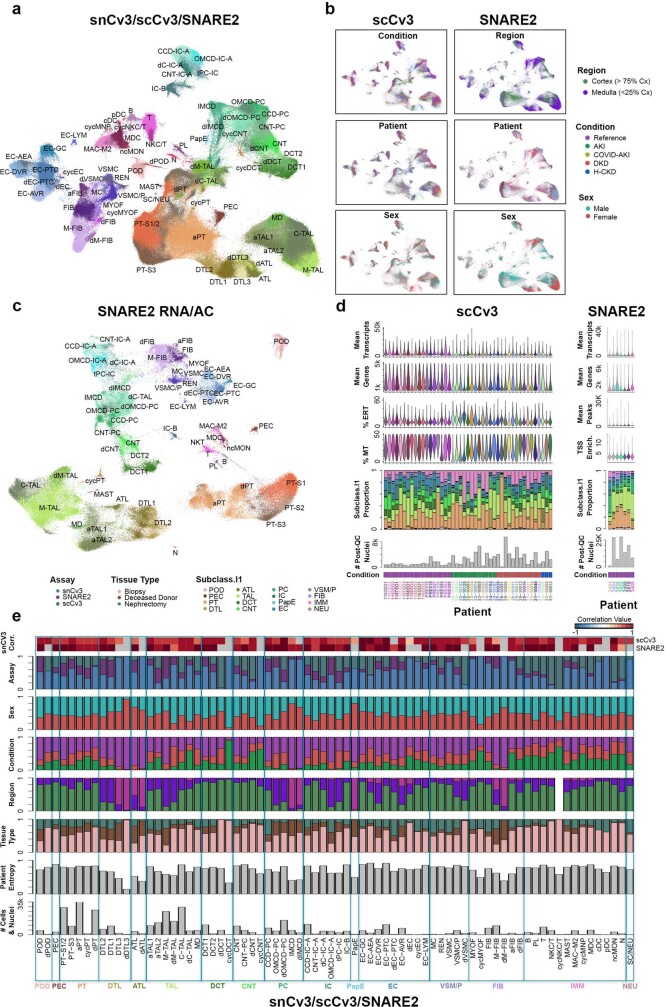
scCv3 integration and quality metrics. **a**. UMAP plot showing integrated snCv3, scCv3 and SNARE2 (RNA) subclass level 3 annotations. scCv3 and SNARE2 (RNA) datasets were projected onto the snCv3 embeddings. **b**. UMAP plots as in (**a**) show mapping of the corresponding sex, patient identities and conditions for scCv3 and SNARE2 datasets. **c**. Joint embedding of SNARE2 RNA and AC modalities. **d**. Barplots showing the total number of post-QC nuclei and subclass level 1 cell types detected per scCv3 or SNARE2 patient. Violin plots show the percentage of transcripts associated with the mitochondria (Mt) or endoplasmic reticulum (ER), as well as mean genes, mean transcripts, mean accessible peaks or mean TSS enrichment scores detected per patient. **e**. Barplots showing the total number of post-QC nuclei/cells per subclass (level 3) combined across platforms (snCv3, scCv3, SNARE2). Patient entropy as well as tissue type, region, condition, sex and assay proportions are shown. Heatmap of correlation values for each scCv3 and SNARE2 subclass against the corresponding snCv3 subclass is shown (top panel). Grey values indicate absence of a comparison where subclasses were not covered by one or more of the technologies. Source Data

To fully examine the molecular profile of kidney cell types, we used droplet-based transcriptomic assays (Chromium v3) for single nuclei (snCv3) and single cells (scCv3) and the multiomic assay for single-nucleus chromatin accessibility and mRNA expression sequencing (SNARE-seq2, or SNARE2)^16–18^ (Supplementary Tables 1–3). Integrative transcriptome analyses were performed on more than 400,000 high-quality nuclei/cells (Methods) from 58 reference tissues (35 donors) and 52 diseased tissues (36 patients) that covered the spectrum of conditions from healthy to AKI and CKD (Fig. 1, Extended Data Figs. 1–3 and Supplementary Fig. 1). Unsupervised clustering was first performed on snCv3 data, permitting the discovery of 100 distinct cell populations, which were annotated to 77 subclasses of epithelial, endothelial, stromal, immune and neural cell types (Fig. 2, Methods, Extended Data Figs. 1 and 2 and Supplementary Tables 4 and 5). To further extend cell type annotations across omic platforms, snCv3 data were used to anchor scCv3 and SNARE2 datasets to the same embedding space, and cell type labels were assigned through integrative clustering (Methods, Extended Data Fig. 3 and Supplementary Tables 6 and 7). For spatial localization of these cell types or states in situ, we applied 3D label-free imaging, multiplex fluorescence imaging (15 individuals) and spatial transcriptomic Slide-seq2^19,20^ (6 individuals, 67 pucks) and Visium assays (22 individuals, 23 samples) (Fig. 1, Methods and Supplementary Table 2). To ensure consistency and agreement of findings across technologies and minimize procurement- and assay-related biases, multiple samples were processed with more than one assay (Supplementary Table 3 and Extended Data Fig. 1a). Our approach permitted deep and cross-validated molecular profiles for aligned kidney cell types, leveraging the distinct advantages of each technology; for example, the addition of cytosolic transcripts from scCv3, regulatory elements from SNARE2 accessible chromatin, and in situ cell type/state localization and interactions from spatial technologies.

## Reference and altered states

**Extended Data Fig. 4:**
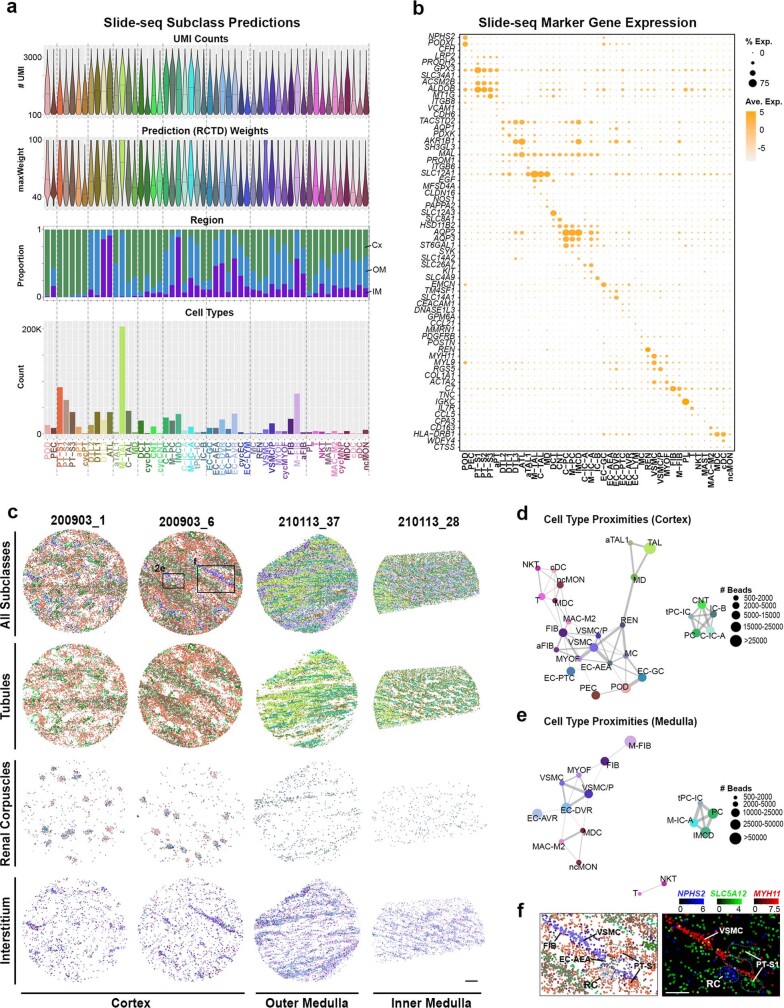
Slide-seq predicted cell types. **a**. UMI counts per bead for classified beads. Normalized RCTD weights for the beads classified at subclass level 2 (Methods). Region of the tissue associated with beads for each subclass. Frequency of cell types predicted across pucks. **b**. Dot plot showing expression of cell type markers identified by snCv3 in the classified Slide-seq beads. **c**. Representative pucks showing subclass level 2 classifications. Cell types are grouped into 3 categories and plotted separately for clarity. Scale bar is 300 µm. **d-e**. Cell proximity networks for Slide-seq cell types associated with cortical or medullary regions. For panels **a**, **b**, **d** and **e** all pucks (6 individuals) were combined. **f**. Left panel: Slide-seq puck area indicated in (**c**) and predicted cell types for the AEAs and surrounding cell types. Right panel: mapped expression values for corresponding marker genes (scaled). AEA mapping over Visium histology is depicted in Extended Data Fig. 5j, colocalized with *REN* expression. Scale bar is 100 µm. Source Data

**Extended Data Fig. 5:**
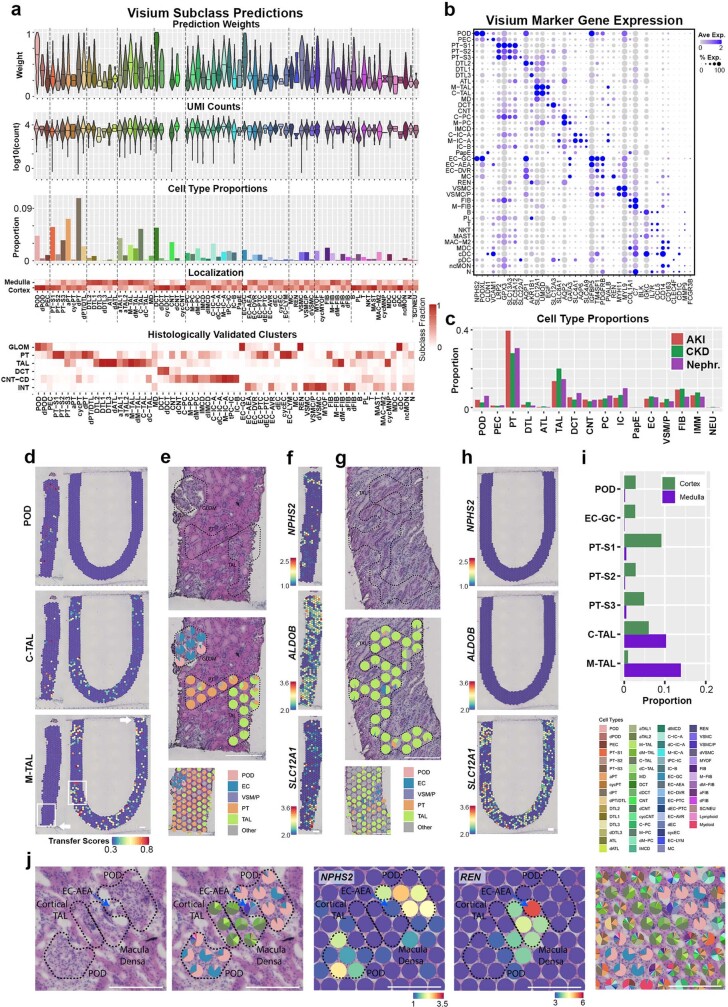
10X Visium predicted cell types. **a**. Analysis of subclass (level 2) predictions on 10x Visium spots (23 samples, 22 individuals). The top panel presents the distribution of transfer scores for the subclass (level 2) with the highest score in each spot. The UMI count panel presents the UMI counts associated with these spots. The cell type proportion panel depicts the proportion of transcriptomic signatures for each subclass, corresponding to its transfer score relative to all non-zero transfer scores in that spot. The relative proportion of cell type subclass signatures arising from the cortex or medulla in the 23 samples is shown. The bottom panel reveals the alignment between the predicted cell type subclass and unsupervised clusters that were histologically validated (Methods). **b**. Dot plot showing gene expression of select cell markers by predicted subclass (level 2) for all 23 Visium samples. **c**. The proportion of transcriptomic signatures in the 23 samples revealed a similar distribution of cell types across healthy reference nephrectomies, chronic kidney disease (CKD), and acute kidney injury (AKI) samples. **d**. Cortical (left, I) and medullary (right, U) portions of specimen 21-0063 reveal POD signatures confined to the cortex, while M-TAL signatures were found in the medulla. White arrows denote the connection point between the cortex and medulla portions of the sample. **e**. A histologic image of the cortex (bounded in **d**) reveals level 1 cell type mapping of POD, EC-GC, and VSM/P cells to a glomerulus. PT and TAL signatures were seen mapped over distinct regions of tubules. **f**. Expression of *NPHS2* (for glomeruli), *ALDOB* (for PT), and *SLC12A1* (for TAL) in the cortex. **g**. A histologic image of the medulla (bounded in **d**) reveals level 1 cell type mapping of a high proportion of TAL cells within the medulla. **h**. Feature plots showing *SLC12A1* but not *NPHS2* or *ALDOB* expression in the medulla. **i**. Proportion of cortex and medulla cell types for sample 21-0063 (9555 total spots over two sections of the same individual). **j**. A cortical image in a healthy reference sample (19-M61) showing EC-AEA entering the glomerular corpuscle near the MD. Two glomeruli contain signatures arising predominantly from POD and EC-GC. Two TAL niches are outlined. TAL niche 1 is enriched in healthy cortical TAL signature and TAL niche 8 near the afferent arteriole is enriched for Macula Densa (MD) signature. *NPHS2* expression is found within the glomeruli and renin (*REN*) expression is highest in the EC-AEA. A full level 2 cell type deconvolution is provided in the final panel (right). Scale bars are 300 μm in length. Source Data

We provide a very high level of complexity for all cell types along the depth of the kidney from the cortex to the papillary tip, in each nephron segment and the interstitium (Fig. 2a), identifying 51 canonical human kidney cell types with associated biomarkers (Methods and Supplementary Tables 5−8). This includes cell type epigenetic maps, comprising open chromatin regions and *cis*-regulatory elements with enriched transcription-factor-binding motifs (Supplementary Fig. 1 and Supplementary Table 9). To spatially localize cell types within the tissue, snCv3 subclasses were used to predict identities in Slide-seq and Visium transcriptomic data at different resolutions (10 µm and 55 µm beads, respectively) (Fig. 2c–g, Methods and Extended Data Fig. 4–5). This enabled us to recapitulate renal corpuscle, tubular, vascular and interstitial cell types with proportions, marker profiles and spatial organizations consistent with expected or observed (Visium) histopathology (Extended Data Fig. 5). Proximity enrichment analysis based on the cell type composition of adjacent Slide-seq beads across 32 cortical and 35 medullary tissue pucks (6 participants) delineated region-specific cellular neighbourhoods (Extended Data Fig. 4d,e), including the renal corpuscle composition of podocytes (PODs), glomerular capillaries (EC-GC), mesangial cells and parietal epithelial cells. These renal corpuscle neighbourhoods localized adjacent to the juxtaglomerular apparatus cells—renin-producing granular (REN) cells and macula densa cells—and endothelial cells of the afferent/efferent arterioles (EC-AEA) leading into and out of the renal corpuscle (Fig. 2e–f). This neighbourhood analysis further confirmed a distinct vascular smooth muscle cell (VSMC) population flanking the afferent/efferent arterioles (Extended Data Fig. 4f). Consistent with these annotations, we validated the appropriate localization of associated cell type markers across platforms (Fig. 2f and Extended Data Fig. 5d–j). In addition to the renal corpuscle, we spatially anchored cell type subpopulations to the cortex or medulla (Fig. 2c and Extended Data Fig. 5a). The transition of the ascending thin limbs (ATL) of the inner medulla to the medullary thick ascending limb (M-TAL) of the outer medullary stripe was observed in Slide-seq (Fig. 2c), along with the transition from descending thin limb (DTL2) and M-TAL in the medulla to the cortical thick ascending limb (C-TAL) in the cortex in Visium (Fig. 2g and Extended Data Fig. 5d). Thus, the unique strengths of each spatial technology enabled the validation of our omic-defined cell types.

A critical and new element of this reference atlas is the characterization of cellular states associated with pathophysiological stress or injury. We carefully defined these altered states on the basis of previous studies and known features of injury (Methods and Supplementary Table 10). We established multiple putative states—namely cycling, transitioning, adaptive (successful or maladaptive repair) and degenerative (damaged or stressed). These altered states were identified for epithelial cells along the nephron, as well as within the stroma and vasculature (Fig. 2a,d). Altered states, from reference and disease tissues in different proportions, were found to exist across technologies (Extended Data Figs. 1 and 3) and showed distinct expression signatures (Supplementary Tables 11–15).

**Extended Data Fig. 6:**
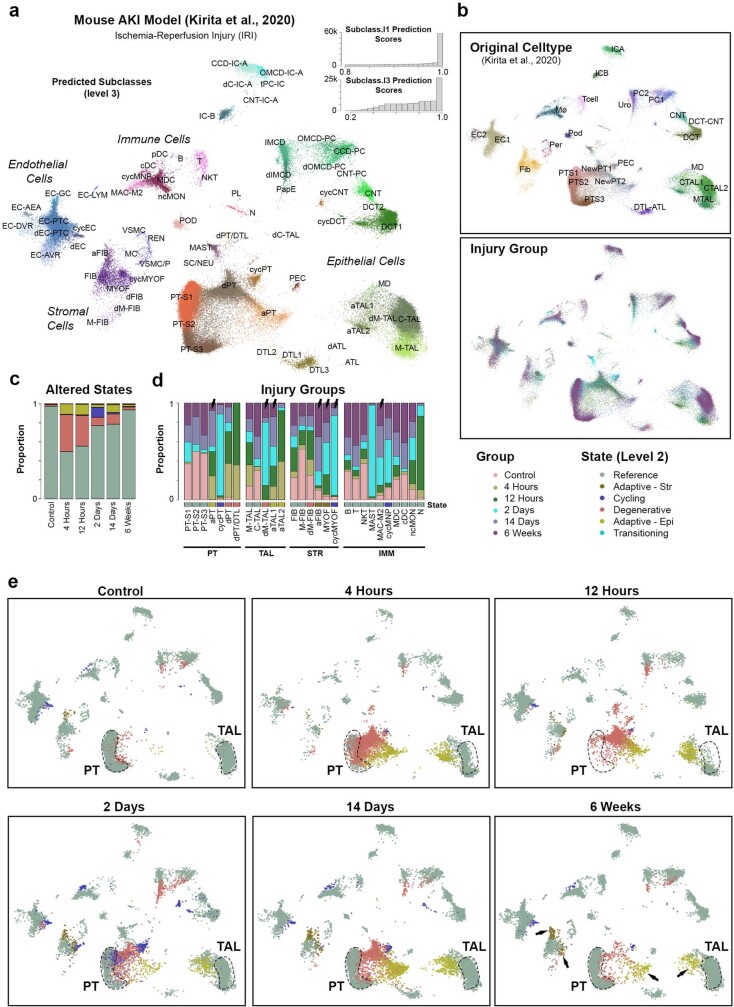
Altered states in a mouse model of AKI. **a**. UMAP showing mouse AKI (IRI) data^4^ with cell types predicted from snCv3. Mouse datasets were projected onto the snCv3 UMAP embeddings (Fig. 2b). Histograms of prediction scores for subclasses (level 1 and 3) are shown. **b**. UMAP plots as in (**a**) showing the original cell type annotations^4^ and injury groups (time points following IRI) for mouse data. **c**. Barplot showing the proportion of altered states for each mouse injury group. **d**. Barplot showing proportion of each injury group for a subset of predicted subclasses. Arrows indicate altered states or immune cells (MAC-M2) that persisted at 6 weeks following injury. **e**. UMAP as in (**a**) showing the distribution of reference and altered states over the different injury groups. Source Data

**Extended Data Fig. 7:**
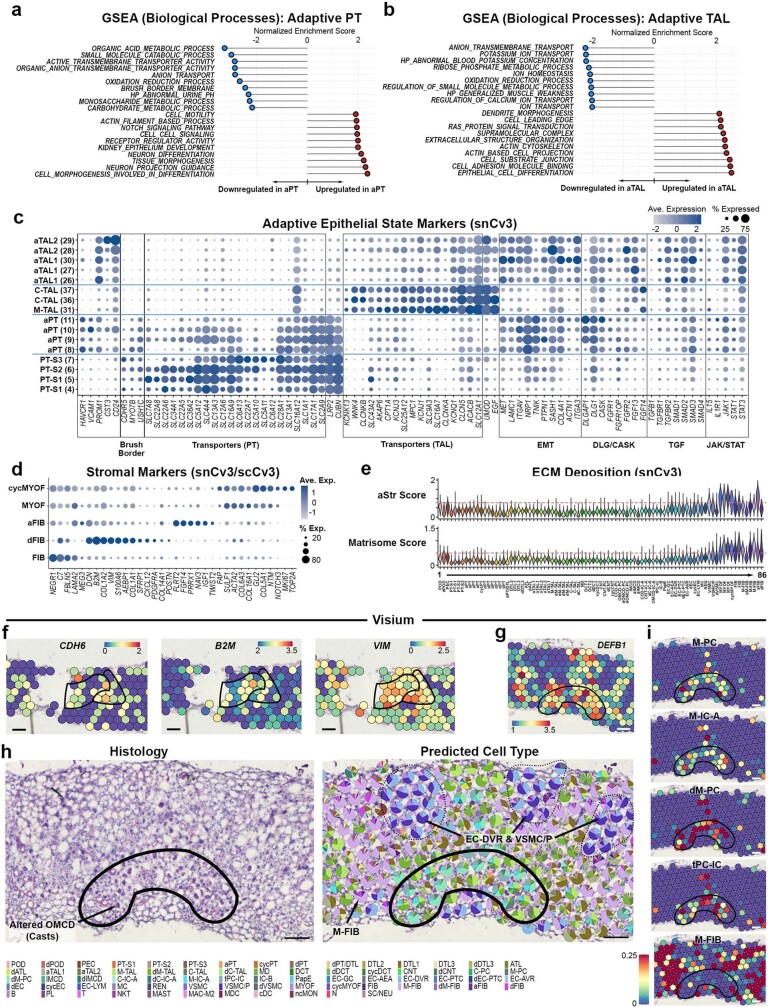
Altered state expression signatures. **a-b**. Gene Set Enrichment Analyses (GSEA) for genes upregulated or downregulated in adaptive PT (**a**) and TAL (**b**) states compared to reference states. **c**. Dot plot showing averaged marker gene expression values (log scale) and proportion expressed for snCv3 clusters. **d**. Dot plot showing averaged marker gene expression values (log scale) and proportion expressed for integrated snCv3/scCv3 reference, degenerative and adaptive stromal clusters. **e**. Violin plots showing aSTR and ECM (matrisome) scores for snCv3 clusters. **f**. Visium feature plots of normalized counts for select markers mapped to regions shown in Fig. 3e. Scale bar is 100 µm. **g**. Visium feature plot of normalized counts for a select marker mapped to region shown in (**h**). Scale bar is 100 µm. **h**. Histology and predicted cell types for a medullary region of acute tubular necrosis (cellular cast formation within tubular lumens, loss of brush border, loss of nuclei, and epithelial simplification). Pie charts are proportions of predicted transfer scores. Area corresponds to the upper bounded region in Fig. 3b. Scale bar is 100 µm. **i**. Predicted transfer scores for area shown in (**h**). Scale bar is 100 µm.

We used several methods to confirm these altered states. Mapping our annotations onto an existing mouse AKI model^4^ provided insights into their timecourse after an acute injury event (Extended Data Fig. 6). Degenerative states, coinciding with elevated expression of the known injury markers *SPP1*, *CST3*, *CLU* and *IGFBP7*^21^ in humans (Supplementary Fig. 2), arose early in mice after injury (Extended Data Fig. 6c–e). These states showed a common expression and regulatory signature across cell types associated with FOS/JUN signalling (Supplementary Fig. 2) and were largely depleted in recovered mouse kidneys, consistent with possible cell death or a progression into repair states. Putative adaptive (successful or maladaptive tubular repair) states were primarily found within the proximal tubule (PT) and TAL subclasses in mouse and human kidneys. Both adaptive epithelial (aEpi) cell types showed expression profiles associated with epithelial differentiation, morphogenesis, mesenchymal differentiation and EMT, while also exhibiting a marked downregulation of transporters critical to their normal function (Extended Data Fig. 7a–c). The adaptive PT (aPT) population both mapped to and correlated with failed repair in rodents (Extended Data Fig. 2g), with characteristic expressions of *VCAM1*, *DCDC2* and *HAVCR1*^4,22^ (Extended Data Fig. 7c). Notably, we now identify a similar state within the TAL (aTAL), marked in humans by *PROM1* (encoding CD133) and *DCDC2* (Supplementary Table 13). These are consistent with CD133^+^PAX2^+^ lineage-restricted progenitors that are known to exist in the proximal and distal tubules of the adult kidney^23,24^. Analysis of the mouse AKI data revealed that these originated predominantly from C-TAL, and followed a similar time course as aPT, persisting 6 weeks after AKI, consistent with a potential failed-repair population^4^. This suggests a common aEpi state, sharing molecular signatures associated with injury and repair, that occurs in higher abundance within the PT and cortical TAL.

Distinct altered states were identified within the stroma (aStr) that were consistent with cell types involved in wound healing and fibrosis after tissue injury^25^ (Extended Data Fig. 2i). These cell populations encompass myofibroblasts (MyoF), cycling MyoF (cycMyoF) and a group of adaptive fibroblasts (aFIB) representing potential MyoF progenitors^25^. Their expression signatures included genes encoding periostin (*POSTN*), fibroblast activation protein alpha (*FAP*), smooth muscle actin (*ACTA2*) and collagens (Extended Data Fig. 7d). aStr cells were enriched after mouse AKI, and they persisted at later timepoints (Extended Data Fig. 6d,e). Furthermore, they exhibited high matrisome expression^25^, consistent with their predicted role in extracellular matrix deposition and fibrosis (Extended Data Fig. 7e). Thus, careful annotation of altered states across kidney cell types has provided a means for labelling injury populations. This is important not only for diseased tissues, but also in reference tissues in which they might arise from ischaemic stress during sample acquisition or normal ageing. Key outcomes are the ability to annotate healthy reference cell clusters (Supplementary Fig. 3) as well as providing insights into the pathogenetic mechanisms of disease.

## Spatially mapped injury neighbourhoods

**Fig. 3:**
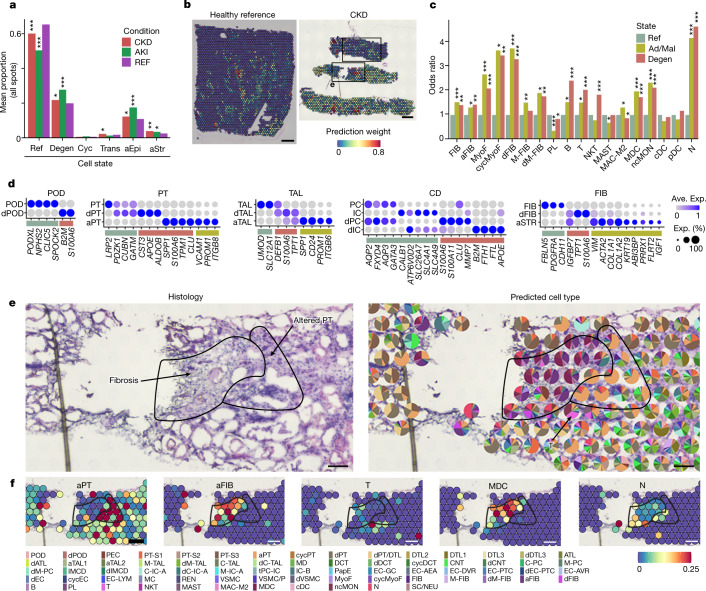
Transcriptomically defined injury neighbourhoods. **a**, The mean proportion of altered-state expression signatures (see Methods, 10x Visium spatial transcriptomics) for all Visium spots (146,460 total spots over 22 individuals). *P* values were calculated using Fisher’s exact tests over the spot proportions. **b**, Feature plots of the aEpi cell state. Scale bar, 300 µm. The top bounded region is shown in Extended Data Fig. 7h. **c**, Colocalization of immune and stromal cells with epithelial cell injury states. The *y* axis shows the odds ratio of colocalization (40,326 total spots over 22 individuals). *P* values were calculated using Fisher’s exact tests over the colocalization events. Ad/Mal, adaptive/maladaptive representing successful or maladaptive tubular repair. **d**, The average expression values for healthy reference and altered-state markers across cell types identified using Visium. **e**, Histology and predicted cell types in a cortical region (CKD) of interstitial fibrosis and neighbouring PT atrophy (altered PT). The pie charts show the proportions of predicted transfer scores for cell type annotations from snCv3 (Fig. 2b). The area corresponds to the bottom bounded region in **b**. Scale bar, 100 µm. **f**, The per-bead predicted transfer scores for cell types for area shown in **e**. Scale bar, 100 µm. *P < 0.01, **P < 1 × 10^−^^5^, ***P < 1 × 10^−10^. Exact *P* values are provided with the Source Data. Source Data

For spatial localization of injury, altered states were mapped to Visium data generated on a range of healthy reference, AKI and CKD tissues (Supplementary Tables 2 and 3). As expected, altered cell state signatures were enriched in AKI and CKD samples compared with in reference tissues (Fig. 3a,b). On the basis of cell type colocalization in the relatively larger area of Visium spots, immune and stromal cells colocalized more frequently with altered epithelial cells (Fig. 3c), consistent with increased fibrosis and inflammation around damaged tubules. Furthermore, cell-type-specific altered states in Visium data that showed expression profiles consistent with snCv3/scCv3 (Fig. 3d) were directly mapped to histological areas of injury. For example, stromal (fibroblast (FIB)), aStr (aFIB) and immune cells (monocyte-derived cells (MDCs)) localized to a region of fibrosis within the cortex of a CKD biopsy (Fig. 3e,f). This region abutted dilated and atrophic tubules that showed an aPT signature marked by *CDH6*^22^ (Extended Data Fig. 7f and Supplementary Table 11). We also found evidence for injury of the medullary tubules (Extended Data Fig. 7g–i), with an area showing intraluminal cellular cast formation, cell sloughing and loss of nuclei that were associated with degenerative CD cells, including degenerative medullary principal cells (dM-PCs) and transitioning principal and intercalated cells. This region increased expression of the degenerative marker *DEFB1*, which was previously shown to contribute to fibrosis through immune cell recruitment^26^. These results support co-mapping of snCv3/scCv3 reference and altered cell types to histological areas of injury.

**Fig. 4:**
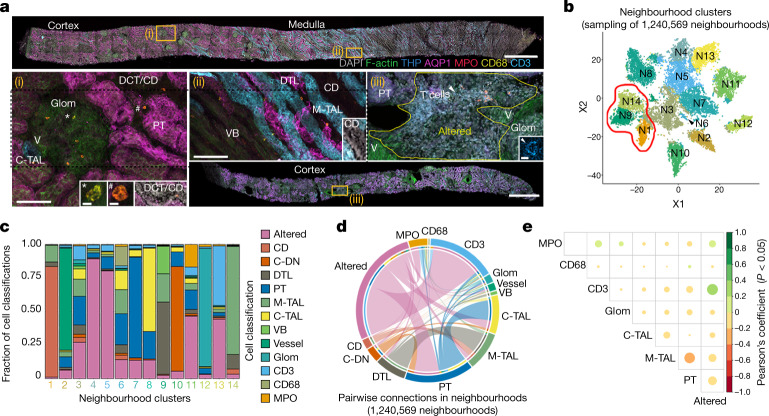
Defining cellular niches in renal disease from 3D fluorescence imaging. **a**, Maximum-intensity projections of representative biopsies (cortex or medulla) showing classification label examples (insets i–iii). Altered, altered morphology or injury; C-DN, cortical distal nephron; Glom, glomeruli; V, vessels; VB, vascular bundle. Examples of MPO^+^ and CD68^+^ are indicated (i). The symbols * and # indicate CD68^+^ and MPO^+^ cells, respectively, in (i) and insets.  Arrowhead indicates T cell in (iii) and inset. Scale bars, 1 mm (biopsy images), 100 μm (i and ii) and 5 μm (insets). **b**, Community-based clustering on cell composition for around 20,000 randomly chosen neighbourhoods (15 individuals). The red outline indicates neighbourhoods including the medulla. **c**, The cellular composition of the neighbourhoods identified in **b**. **d**, Pairwise analysis of cells within 1.2 million neighbourhoods (15 individuals); colours are as indicated in **c**. **e**, Pearson’s coefficients for select interactions, the colour indicates both the value and direction of the correlation. *P* values were generated using two-sided *t*-tests. Source Data

**Extended Data Fig. 8:**
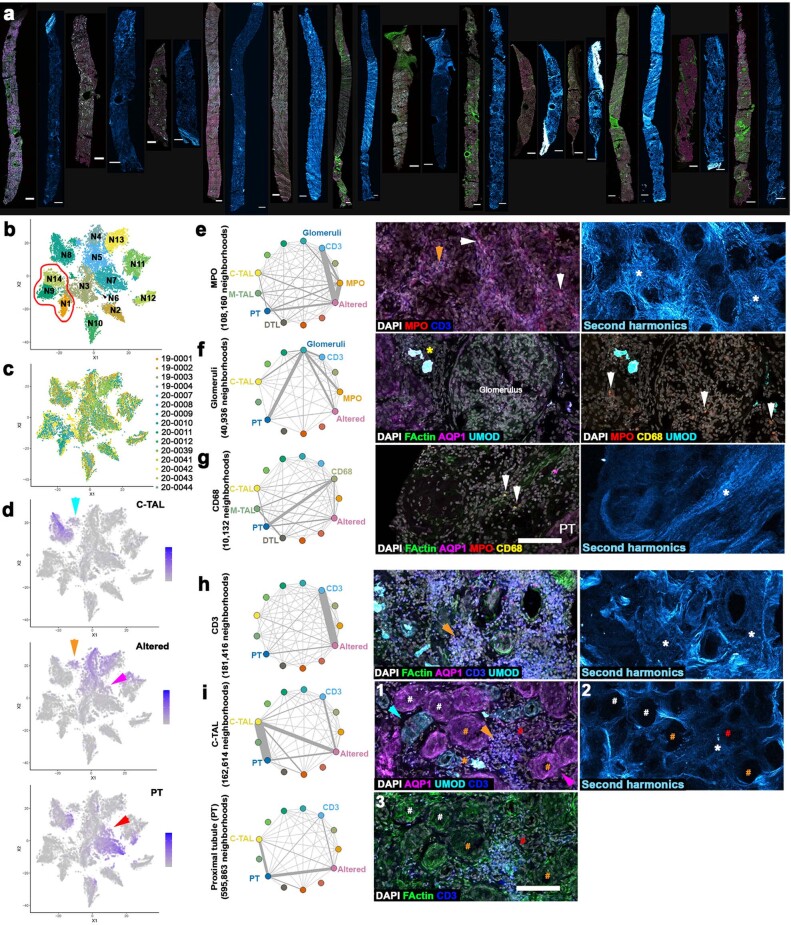
3D imaging identifies injury neighbourhoods. **a**. Maximum intensity projections of immunofluorescence and second harmonic images for 13 example biopsies, scale bars 500 µm. **b**. Overview of neighbourhood classes as given in Fig. 4b for reference. **c**. Distribution of neighbourhoods by specimen in neighbourhood clusters plotted in tSNE space from Fig. 4. **d**. Feature plots of the number of cells per neighbourhood for cortical TAL (C-TAL), altered morphology and proximal tubule (PT). C-TALs and PTs are found in neighbourhoods with altered morphology, cyan and orange vs. red and magenta arrowheads. **e-h**. Neighbourhoods with at least one cell for the labels indicated were subsetted and neighbourhood graphs generated to indicate the pairwise interaction between cell labels. At right: maximum Z-projections of 3D confocal fluorescence images with white arrow indicating MPO+ cells (**e** and **f**) or CD68+ cells (**g**), orange arrows indicating CD3+ cells and asterisks highlighting fibrosis (white) or areas of altered morphology/injury (yellow). Scale bar = 100 μm. **h** and **i**, pairwise subset analysis of CD3+, PT and TAL (orange, magenta and cyan arrows respectively). CD3+ cells cluster in regions of fibrosis (orange arrowhead and white asterisks). UMOD positive casts associate with regions of injury and CD3+ cells (orange asterisk), the tubular epithelium is intact with brush borders (white #), has evidence of epithelial simplification (orange #) or less AQP1 marker and epithelial simplification (red #). Scale bar = 100 µm. Source Data

To further uncover in situ cellular niches and injured microenvironments across kidney disease, we performed 3D multiplexed immunofluorescence imaging and label-free cytometry (3DTC) with second harmonic generation for collagen content^27^ on KPMP AKI and CKD kidney biopsy samples (Extended Data Fig. 8a and Supplementary Tables 2 and 3). 3DTC defined cellular niches for 1,540,563 cells by neighbourhood analysis of 14 classes of cells covering renal cortical and medullary structures (Fig. 4a, Methods and Extended Data Fig. 8b–i). We identified 14 cellular niches through community detection that included expected niches of cortical or medullary epithelium (N7 and N8 versus N14, N9 and N1, respectively; Fig. 4b,c). The TAL and PT neighbourhoods (N7 and N8) were enriched in areas of injury (Fig. 4c and Extended Data Fig. 8i). Furthermore, areas of injury were associated with infiltrating leukocytes, including CD68^+^ (myeloid), MPO^+^ (N) and CD3^+^ (lymphoid or T) cells (N6, N11 and N13, respectively). Uniquely, CD3^+^ cells were almost exclusively detected in a subset of neighbourhoods with areas of tissue damage including presumptive epithelial degeneration (loss of markers and simplification) and fibrosis (N13; Fig. 4a (iii) and 4c and Extended Data Fig. 8h), consistent with degenerative epithelial enrichment found using Visium (Fig. 3c). By contrast, myeloid cells were found in cellular diverse niches with cortical or medullary epithelium (N6 and N11; Fig. 4c). This is consistent with the association of M2 macrophages (MAC-M2) with adaptive rather than degenerative epithelia in Visium data (Fig. 3c) and their sustained presence in mouse ischaemia–reperfusion injury (IRI) (Extended Data Fig. 6d). The leukocyte diversity was specific in 3D neighbourhoods, as MPO^+^ and CD3^+^ cells were overlapping, whereas CD3^+^ cells were conspicuously low in neighbourhoods with CD68^+^ cells (N11 versus N6; Fig. 4c and Extended Data Fig. 8g). As neutrophils colocalized with putative adaptive and degenerative states (Fig. 3c) and transiently infiltrate early in mouse IRI (Extended Data Fig. 6d), neutrophils may infiltrate along with T cells predominantly in areas of acute injury marked by mixed degenerative and adaptive states. Alternatively, myeloid cells (such as MAC-M2) may occur more predominantly within relatively healthy areas showing active repair (adaptive or maladaptive). Overall, the results from spatial transcriptomics, histological correlation and 3DTC demonstrate that altered states were enriched in PT and TAL neighbourhoods, with distinct immune-active cellular niches associated with healthy and injured tubules.

## Stages and niches of epithelial repair

**Fig. 5:**
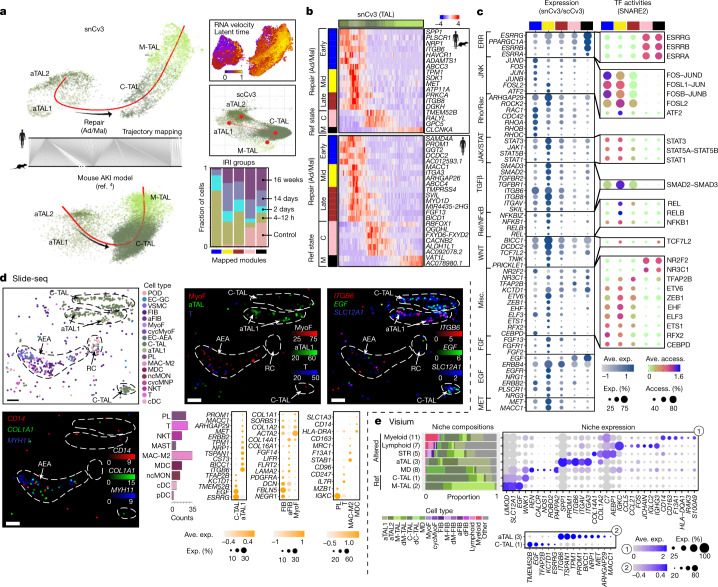
Expression and regulatory signatures of adaptive epithelial cells. **a**, Trajectory of TAL cells for snCv3, scCv3 and mouse AKI^4^ data, showing mouse to human mapping. Top right, latent time heat map from RNA velocity estimates. Bottom right, bar plot of collection groups after IRI across mouse trajectory modules. **b**, Heat map of smoothened gene expression (conserved or human specific) along the inferred TAL pseudotime. State modules based on the gene expression profiles are shown. M, M-TAL; C, C-TAL; Ad/Mal, adaptive/maladaptive, representing successful or maladaptive tubular repair. **c**, SNARE2 average accessibilities (access.) (chromVAR) and the proportion accessible for transcription-factor-binding sites (TFBSs) (right), and the averaged gene expression values (log scale) and the proportion expressed for integrated snCv3/scCv3 modules (left). TF, transcription factor. **d**, Slide-seq fibrotic regions. Top and bottom right, bead locations for a representative region, coloured by predicted subclasses, prediction weights or scaled gene expression values. Marker genes are *ITGB6* (aTAL), *EGF* and *SLC12A1* (TAL), *CD14* (MAC-M2/MDC), *MYH11* (VSMC/MyoF) and *COL1A1* (aStr). The bar plot shows the immune subclass counts and the dot plots show the average expression of marker genes generated from three fibrotic regions (two individuals; Extended Data Fig. 11a). Scale bar, 50 μm. **e**, Visium TAL niches identified from all Visium spots and defined by colocalized cells (Methods and Extended Data Fig. 11b–e), showing the proportion of component cell type signatures. The dot plots show the niche marker gene average expression values. Source Data

**Extended Data Fig. 9:**
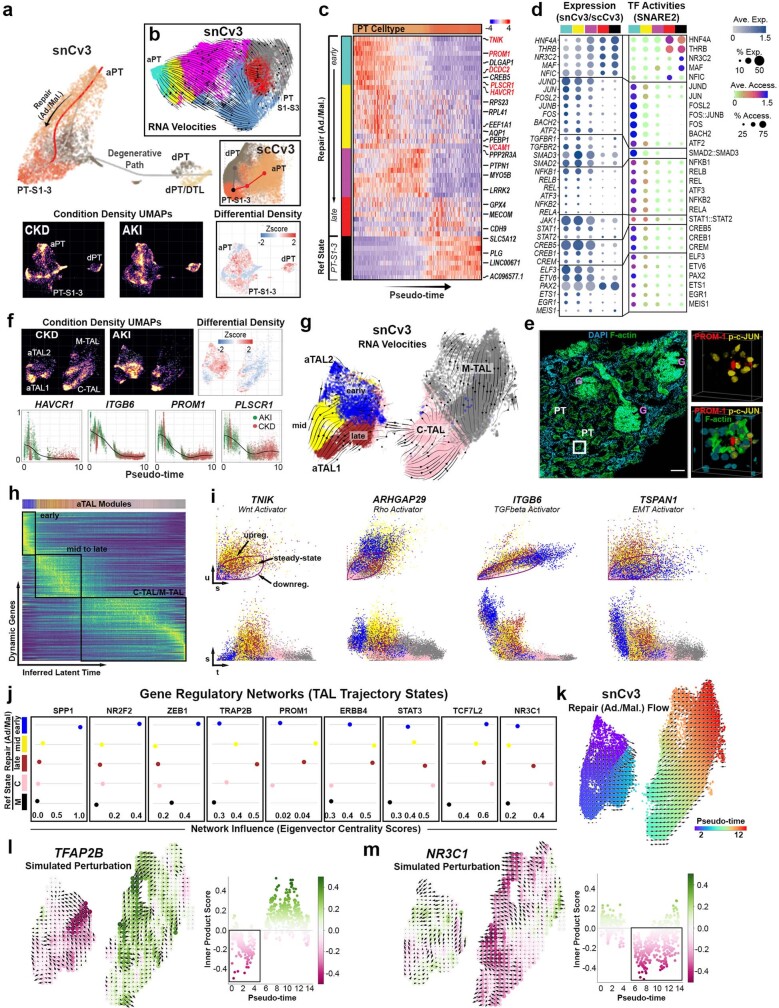
PT and TAL repair trajectories. **a**. Trajectory of PT cells for snCv3 and scCv3 datasets. Bottom UMAPs are coloured by cell density for each condition (AKI/CKD), including the cell density difference between AKI and CKD. **b**. UMAP of PT subclasses (PT-S1-S3, aPT) with projected RNA velocities, derived from a dynamical model of PT repair modules, visualized as streamlines (Methods). **c**. Heatmap of smoothed gene expression profiles along the inferred pseudo-time for PT cells. Colour blocks on the left show different repair states or modules identified based on the gene expression profiles. **d**. Right panel: dot plot of SNARE2 average accessibilities (chromVAR) and proportion accessible for TFBSs showing differential activity in aPT modules. Left panel: dot plot of averaged gene expression values (log scale) and proportion expressed for integrated snCv3/scCv3 modules. **e**. 3D confocal imaging of a reference kidney tissue section stained for PROM-1 (red), Phopho-c-Jun (p-c-JUN, yellow), F-actin (with FITC phalloidin, green) and DNA with DAPI (cyan) (scale bar 100 µm). Regions of PROM-1 within a glomerulus (G) and a proximal tubule (PT) are indicated and enlarged in the right panels (rendered 3D volumes, scale bar 10 μm). This area shows the association of PROM-1 expression with p-c-Jun+ cells in the tubules. 3D rendering was performed using the Voxx software from the Indiana Center for Biological Microscopy (voxx.sitehost.iu.edu/). **f**. Top panels: TAL UMAPs as in Fig. 5a (snCv3) showing condition densities as in (**a**). Bottom panels: changes of smoothed gene expression (snCv3) for representative genes as a function of inferred pseudotime coloured by disease conditions. **g**. TAL UMAP as in Fig. 5a (snCv3) with projected RNA velocities, derived from a dynamical model for TAL repair modules, visualized as streamlines (Methods). **h**. Heatmap showing expression value dynamics (snCv3) along latent time inferred from RNA velocities for the top 300 likelihood-ranked genes. Top colour bar indicates aTAL repair modules. **i**. Scatter plots (u, unspliced; s, spliced; t, latent time) for putative driver genes (snCv3) identified by high likelihoods in the dynamical model. **j**. Gene regulatory networks associated with TAL repair modules (Methods, see Supplementary Table 23). Eigenvector centrality scores were plotted for select factors with high influence on different states. **k**. UMAP embedding (snCv3) showing pseudotime gradient and the derived vector field associated with TAL repair. **l-m**. UMAP embedding showing simulated vector fields following *TFAP2B* (**l**) or *NR3C1* (**m**) perturbation. Barplots show inner product calculations (perturbation scores) comparing directionality and size of TAL repair flow vectors and the simulated perturbation vectors. Negative perturbation scores indicate a block in differentiation.

To obtain a deeper understanding of the genetic networks underlying the progression and potential pathology of altered tubular epithelium, we performed trajectory inference on the snCv3/SNARE2 and scCv3 subpopulations (Fig. 5a,b, Methods and Extended Data Fig. 9). Although most degenerative states appeared too disconnected, aEpi trajectories showed dynamic gene expression and regulatory transitions from dedifferentiated to mature functional states (Supplementary Tables 16–21). We further identified transitory states or modules that may be associated with either successful or maladaptive repair. Early repair cells showed expression signatures associated with progenitor states (*PROM1*), microtubule reorganization (*DCDC1*) and AKI (*HAVCR1*, *SPP1*) (Fig. 5b and Extended Data Fig. 9c,f). The directionality of these repair trajectories was confirmed from RNA velocities estimated from dynamical modelling of transcript splicing kinetics, and the alignment with mouse AKI subpopulations (Fig. 5a and Extended Data Fig. 9b,g). These analyses enabled the identification of TAL repair signatures that were either conserved across species or human specific (Fig. 5b).

Epithelial repair signalling was enriched for several growth factors and pathways with known roles in promoting normal tubulogenesis, as well as maladaptive repair, fibrosis and inflammation. These include Wnt, Notch, TGF-β, EGF, MAPK (FOS/JUN), JAK/STAT and Rho/Rac signalling^28–36^ (Fig. 5c, Extended Data Fig. 9d and Supplementary Tables 19–21), with dynamic transcription of several pathway regulators mapped to the TAL repair modules (Extended Data Fig. 9h, i). In support of MAPK signalling, PT cells that showed expression of PROM1 were subjacent to phosphorylated JUN (p-JUN) (Extended Data Fig. 9e). Progressively active REL/NF-κB signalling along the aTAL and aPT trajectories further expands on previous roles for this pathway in injured PTs^15^ (Fig. 5c and Supplementary Table 19). We also found increased cAMP signalling (CREB transcription factors in aPT) capable of promoting dedifferentiation^37^ and increased ELF3 activities that are potentially required for mesenchymal–epithelial transition^38^, both indicating that adaptive states may be poised for re-epithelialization.

Through integration of SNARE2 epigenomic profiles with snCv3 transcriptomes, detailed gene regulatory networks (GRNs) were inferred for TAL trajectory modules. Transcription factors with high network importance were identified in each repair state, confirming key roles for several major signalling pathways, including their downstream target genes and processes (Extended Data Fig. 9j and Supplementary Tables 22–24). This highlighted a critical role for TRAP2B (AP-2β), which was previously found to be required for terminal differentiation of distal tubule cells through activated expression of *KCTD1*^39^. Both factors were active or expressed within mid-repair states (Fig. 5c) and simulated perturbation of *TRAP2B* disrupted the repair trajectory transition (Extended Data Fig. 9l,m). We therefore find adaptive epithelial trajectories sharing common molecular profiles that progressively upregulate cytokine signalling involved in tubule regeneration, while also providing molecular links to pathways associated with fibrosis, inflammation and end-stage kidney disease.

**Extended Data Fig. 10:**
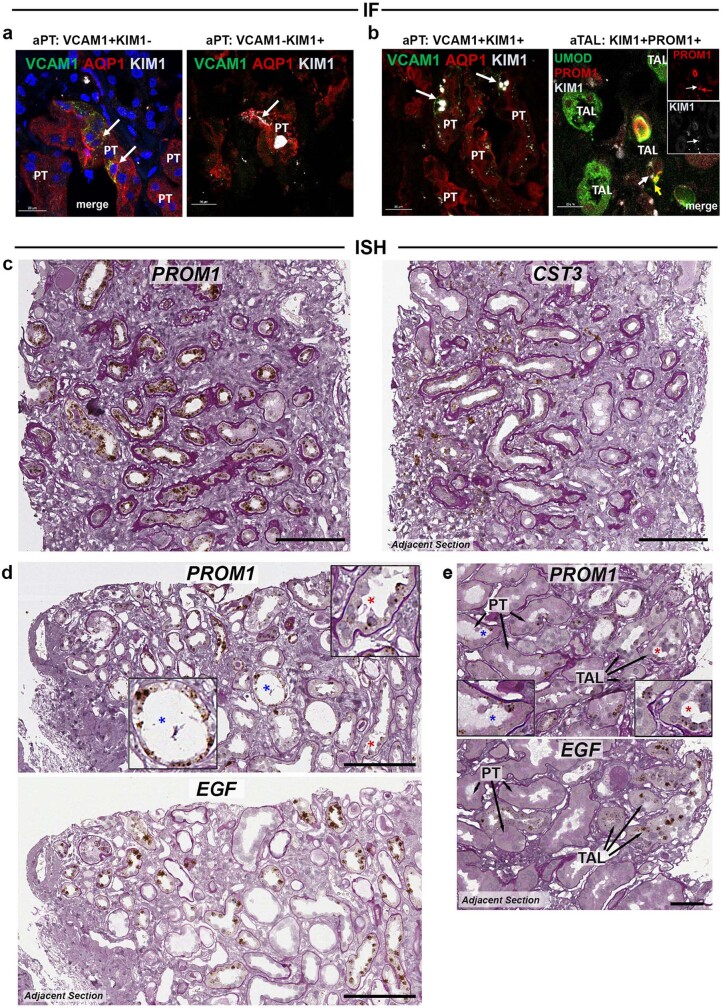
Adaptive epithelia localized to areas of injury. **a**. Immunofluorescent (IF) staining of VCAM1, AQP1, KIM1 (HAVCR1) in the aPT (performed on replicate sections from 3 individuals). Scale bars represent 20 µm. **b**. IF staining of UMOD, PROM1 and KIM1 in the TAL (performed on replicate sections from 3 individuals). Scale bars represent 20 µm. **c-e**. RNA *in situ* hybridization (ISH) for *PROM1*, *CST3* or *EGF* (performed on adjacent sections from 6 individuals). **c**. ISH for *PROM1* and *CST3* in adjacent sections. *PROM1* is localized to an area showing interstitial fibrosis and tubular atrophy. Scale bar is 100 μm. **d**. RNA ISH for *PROM1* (left panel) and *EGF* (right panel) in adjacent corticomedullary sections. *PROM1* positive epithelial cells seen in injured tubules (epithelial simplification, loss of nuclei) that are *EGF* negative (blue asterisks, upper inset image) and *EGF* positive healthy TAL (red asterisks, lower inset image). Scale bar is 100 μm. **e**. ISH for *PROM1* and *EGF* (healthy TAL) showing *PROM1* localization to PT (blue asterisks, left inset) and TAL (red asterisks, right inset) showing histological evidence of injury (epithelial thinning, nuclei loss, brush border loss in PT). Adjacent section (lower panel) shows *EGF* positivity in healthy TAL cells. Scale bar is 50 μm.

**Extended Data Fig. 11:**
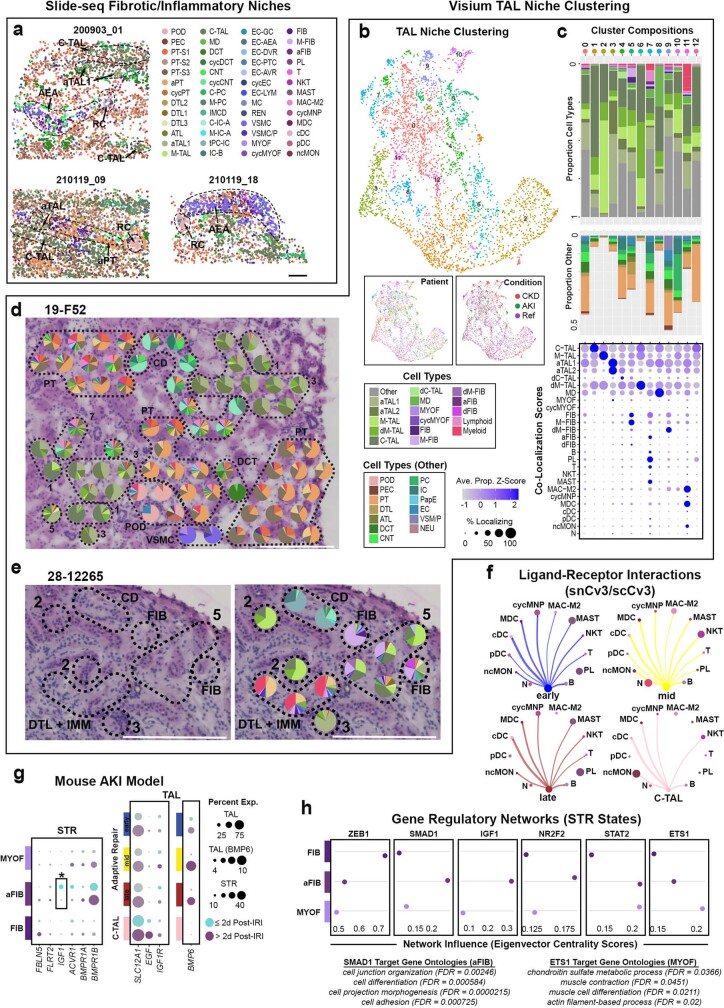
TAL adaptive or maladaptive repair niches. **a**. Slide-seq fibrotic/inflammatory niches from Fig. 5d showing full predicted subclass level 3 cell type distributions. Scale bar is 100 μm. **b**. Visium TAL niches were identified by clustering TAL dominant spots according to Seurat label transfer scores. The UMAP denotes 13 TAL niches which were distributed across the 23 samples (patient inset) and across disease state conditions (condition inset). **c**. Visium niche cluster compositions. Signature proportions of TAL cell types, injury cell states, stromal cells, and immune cells. Niche 5 contained significant stromal, niche 7 contained lymphoid, and niche 11 contained myeloid cell signatures. Some niches (e.g. 9) had significant contributions from neighbouring non-TAL epithelial cells (“Proportion Other” bar plot). The colocalization score (Methods) for cell types within each niche is based on Seurat label transfer scores and provided as a dot plot. **d**. A subset of TAL niches (1, 3, 5, 7) were overlaid upon a histologic image of the cortex in sample M19-F52_3, with each niche often represented by multiple contiguous spots. Scale bar is 300 μm in length. **e**. Representative region (patient 28-12265) showing niche 5 (STR) localized in proximity to interstitial fibrosis, and niche 3 (aTAL) localized adjacent to myeloid cell infiltration. Scale bar is 300 μm. **f**. Circle plot of ligand-receptor cell cell communications between TAL repair modules or states and immune cell subclasses. Dot size indicates relative proportion of the subclasses and TAL module, edge width represents strength of the communication. **g**. Dotplots showing expression level and percent expressed for select ligands or receptors within the mouse AKI data. Data were grouped into injury groups less than or equal to 2 days (including control cells) and groups greater than 2 days post-injury. The asterix highlights an *IGF1* expression difference found between early and late injury groups of the aFIB population. **h**. Gene regulatory networks associated with STR cell types (see Supplementary Table 27). Eigenvector centrality scores were plotted for select factors with high influence on different subclasses. Ontologies for target genes downstream of select transcription factors are shown. Source Data

Slide-seq, Visium, immunofluorescence staining and RNA in situ hybridization (ISH) experiments confirmed spatial localization of adaptive states into injury niches (Fig. 5d,e and Extended Data Fig. 10). aTAL populations in Slide-seq-processed tissues (3 niches, 2 individuals; Fig. 5d and Extended Data Fig. 11a) were marked by an upregulation of the aTAL marker *ITGB6* and downregulated *EGF* expression, which is known to occur after TAL injury^40^. These were identified adjacent to areas of aStr enrichment, evidenced by elevated *COL1A1* expression. These potentially fibrotic regions also showed diverse inflammation for both lymphoid (T cell) and myeloid (MAC-M2/MDC) cell types that co-localized around vessels (Fig. 5d). Analogously, aTAL injury niches were identified in Visium data as spots (55 µm) colocalizing with stromal, lymphoid and myeloid cells (Fig. 5e, Methods and Extended Data Fig. 11b–e). Localization of aTAL states to injured tubules was further confirmed by ISH, in which *PROM1-*expressing cells showed clear histological evidence of injury, including epithelial simplification (thinning), loss of nuclei and loss of brush border in PTs (Extended Data Fig. 10e). Overall, aTAL, aStr and immune expression profiles from spatial transcriptomics were consistent with those identified from snCV3 and scCv3, providing both validation and spatial co-localization of these cell types and states into niches of ongoing injury and repair.

**Fig. 6:**
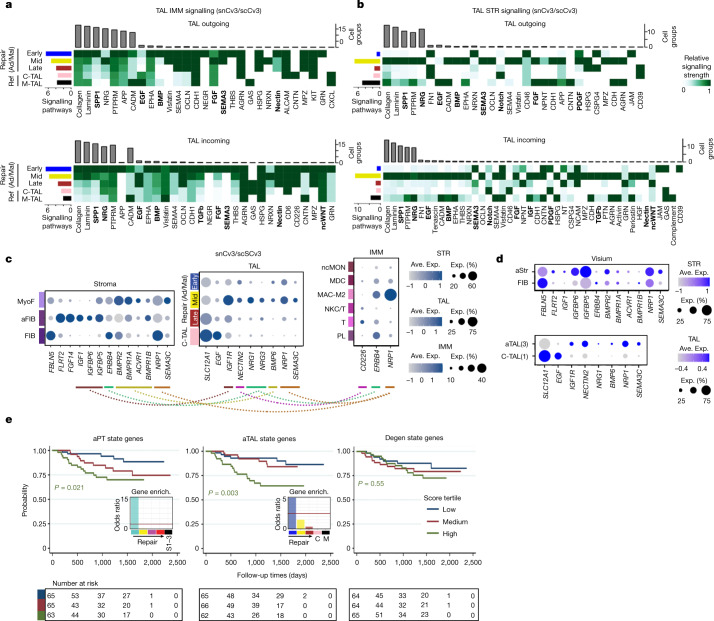
Maladaptive repair signatures. **a**,**b**, The ligand–receptor signalling strength between TAL states and IMM subclasses (**a**) or STR subclasses (**b**). The coloured bars indicate the total signalling strength of the cell group by summarizing signalling pathways. The grey bars indicate the total signalling strength of a signalling pathway by summarizing cell groups. Members of key signaling pathways described in the main text are in bold. **c**, The average gene expression values for select ligand–receptor combinations using snCv3/scCv3 integrated data. **d**, Dot plots validating select markers shown in **c** in the Visium data. **e**, Unadjusted Kaplan–Meier curves by cell state scores for composite of end-stage renal disease (ESRD) or for 40% drop in eGFR from time of biopsy in the NEPTUNE adult patient cohort (199 patients; Supplementary Table 30). Patients who reached the end point between screening and biopsy were excluded. Enrich., enrichment. *P* values calculated using log-rank tests for trend are shown (*P* = 0.021 (aPT), *P* = 0.003 (aTAL), *P* = 0.55 (degenerative)).

Given the upregulation of fibrotic cytokine signalling in epithelial repair, these regenerating cells may represent maladaptive states if they accumulate or fail to complete tubulogenesis. We therefore investigated the contribution of these states to cell–cell secreted ligand–receptor interactions within a fibrotic niche (Supplementary Table 25). From spatial assays, this niche may comprise aEpi cells adjacent to normal and altered arteriole cells and fibroblasts, and immune cells that include lymphoid and myeloid cells (Figs. 3–5). Using snCv3 and scCv3 datasets associated with trajectory modules, we identified aTAL repair states as having a higher number of interactions first with immune cells (early repair), then with the stroma (mid-repair; Fig. 6a,b). This was associated with secreted growth factors of the FGF, BMP, WNT, EGF, IGF and TGF-β families and the gain of interactions with MAC-M2 and T cells (Extended Data Fig. 11f). This indicates that adaptive tubule states may recruit activated fibroblasts and MyoF both primarily and secondarily through their recruitment of immune cells.

We also found additional evidence for the activation of EGF pathway signalling within the adaptive epithelial trajectories, which in itself may lead to activation of TGF-β signalling and create a niche capable of promoting fibrosis^36^. Consistently, EGF ligands NRG1 and NRG3 both become expressed in aEpi states for a possible role in stromal cells (STR) and MAC-M2 recruitment (Figs. 5d,e and 6c,d). Early and mid-repair TAL states may also recruit or stimulate T cells through expression of the CD226-interacting protein NECTIN2 (Fig. 6c,d). Alternatively, BMP6 signalling from mid-repair states may have a role in preventing fibrosis^41^ through possible SMAD1 activation of fibroblast differentiation within aFIB populations (Fig. 6c,d, Extended Data Fig. 11g,h and Supplementary Tables 26–28). *BMP6* expression was also detected in repair states of the mouse AKI model at late timepoints when aFIB cells already showed reduced *IGF1* expression (Extended Data Fig. 11g). IGF1 secreted from aFIB cells may signal to both stimulate MYOF differentiation^42^ and promote regeneration of the repairing epithelial cells through IGF1R^43^ (Fig. 6c,d). Given the timing of *BMP6* and *IGF1* expression after acute injury, BMP6-induced differentiation pathways within the aFIB cells may represent a late aTAL signal to dampen the fibroblast response. We therefore identify state- and niche-dependent signalling for reparative states in proximal and distal tubules that may ultimately influence the extent of fibrosis and inflammation.

## Adaptive states can be maladaptive

**Extended Data Fig. 12:**
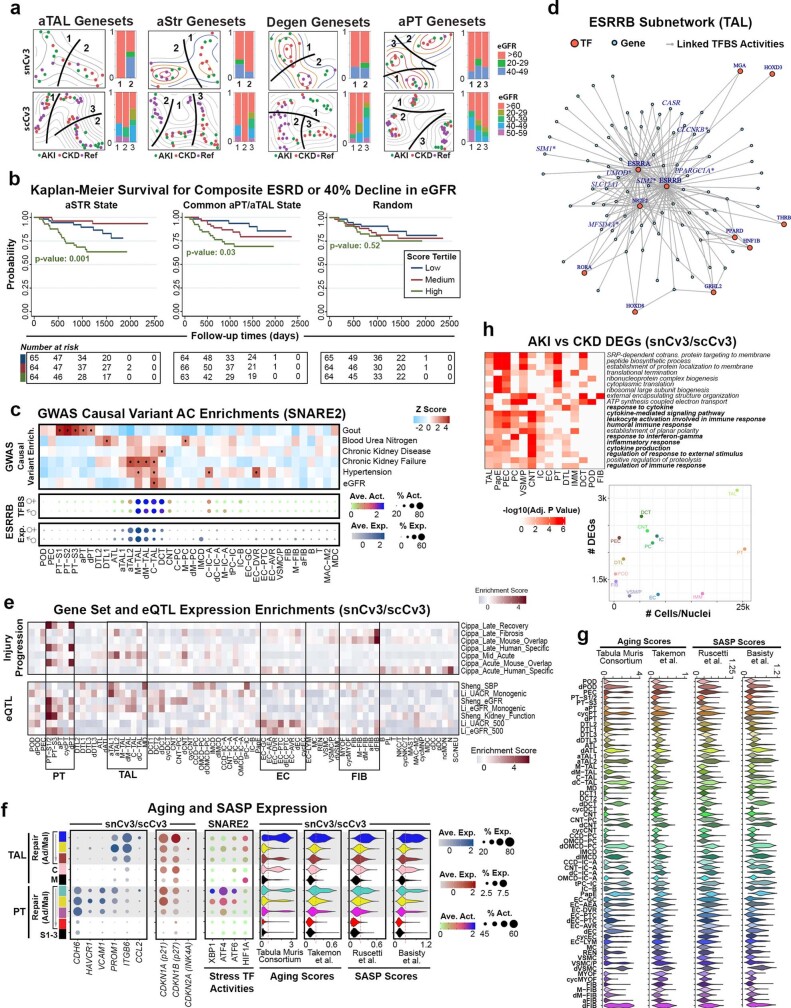
Association of cell state scores with clinical phenotypes. **a**. Embedding plots: grouping of patient-level expression profiles for the aTAL, aStr, Degen, and aPT genesets used for clinical outcome association (Supplementary Table 27) for snCv3 (Top) and scCv3 (Bottom). Barplots: the distribution of eGFR among the identified groups. **b**. Unadjusted Kaplan Meier curves by aStr (*P* = 0.001) and common aPT and aTAL (*P* = 0.03) state scores for composite of ESRD or 40% drop in eGFR from time of biopsy in Neptune adult patient cohort (see Supplementary Table 30). A score generated using 100 randomly selected genes failed to show any correlation (*P* = 0.52) with disease survival. **c**. Heatmap of causal variants (z-scores) that were enriched in SNARE2 cell-type specific accessible chromatin. Dots represent Z-scores > 2 (or *P* value < 0.05). Dotplots show averaged ESRRB binding site accessibility or gene expression (log values) and percent accessible or expressed. **d**. ESRRB subnetwork of TF connections to target genes generated using SNARE2 RNA and AC data, demonstrating a central role for ESRRB in regulating TAL marker genes. Inset shows the ESRRB motif. Boxes represent ESRRB target genes showing causal variant enrichment (**c**) within linked regulatory regions (AC peaks). **e**. Heatmap showing enrichment scores (scaled -log10(p values)) for the RNA expression (snCv3/scCv3) of gene sets associated with eQTL linked to kidney function or disease^88,89^ or associated with progression of acute to chronic injury^90^. **f**. Dot plots of averaged gene expression values (snCv3/scCv3) or TF binding site accessibilities (SNARE) and proportion expressed/accessible. Violin plots show gene expression scores for gene sets associated with aging (Tabula Muris Consortium^48^ and Takemon et al.^69^) or SASP (Ruscetti et al.^70^ or Basisty et al.^71^). **g**. Violin plots showing expression scores for gene sets shown in (**f**) for all non-immune subclasses. **h**. Bottom: Number of differentially expressed genes between AKI and CKD cases for each major cell type in snCv3 and scCv3 datasets. Top: enrichment of functional gene ontology terms for each major cell type. Colour indicates -log adjusted p-value (derived from GSEA and calculated based on permutation). Source Data

Although recruitment of stromal and immune cells is necessary for normal wound healing, persistent recruitment by aEpi cells may impair epithelial function or lead to continued release of cytokines promoting disease progression. Consistent with this, we found that aEpi gene signatures that were conserved across snCv3 and scCv3 (Supplementary Table 29) were associated with poor renal function in CKD cases (Extended Data Fig. 12a). Thus, successful or maladaptive repair within the TAL may have a role in the transition to chronic disease. Notably, aTAL signatures underlying early repair states were significantly associated with disease progression using unadjusted and sequentially adjusted survival models within the Nephrotic Syndrome Study Network (NEPTUNE) cohort of 193 patients^44^ (Fig. 6e, Methods, Extended Data Fig. 12b and Supplementary Table 30). Furthermore, in an independent cohort of 131 patients with kidney disease in the European Renal cDNA Bank (ERCB) cohort, aEpi scores varied by kidney disease diagnosis relative to living donors^45^. Specifically, patients with diabetes, hypertension and focal segmental glomerular sclerosis had higher aPT and common aPT–aTAL signatures compared with that of living donors after adjusting for age and sex. In the diabetes group, the aPT and common aPT–aTAL signatures remained higher than that of living donors even after adjusting for age, sex and estimated glomerular filtration rate (eGFR; Methods and Supplementary Table 30). Nevertheless, it is important to note that the clinical correlations are based on a small sample size and should therefore be interpreted with care.

These findings indicate that altered TAL functionality, including its GFR-regulatory role through tubuloglomerular feedback, may represent a major contributing factor to progressive kidney failure. Furthermore, causal variants for eGFR and chronic kidney failure were enriched within TAL regulatory regions that were also enriched for oestrogen-related receptor (ESSR) transcription-factor motifs (Extended Data Fig. 12c and Supplementary Table 31). ESRR transcription factors (especially ESRRB), which are key players in TAL ion transporter expression^46^, are central regulators of the TAL expression network (Extended Data Fig. 12d), become inactivated in adaptive states (Fig. 5c) and, in experimental models, could exacerbate AKI and fibrosis^47^. Expression quantitative trait loci (eQTL) associated with kidney function that were previously shown to be enriched primarily in PTs also showed enrichment within the TAL, along with signatures associated with acute injury and fibrosis in a human AKI to CKD progression study (Extended Data Fig. 12e). Thus, we demonstrate both a potential maladaptive role for the aEpi states and a potential central role for the TAL segment in maintaining the health and homeostasis of the human kidney. This is consistent with the finding that the top renal genes showing decline in a mouse ageing cell atlas were associated with the TAL^48^.

Our findings implicate an accumulation of maladaptive epithelia during disease progression that may also be consistent with chronically senescent cells^5^. This is supported by both increased expression of ageing-related genes, stress-response transcription factor activities and an apparent senescence-associated secretory phenotype (SASP) for these cells (Extended Data Fig. 12f,g). As such, we detected *CDKN1A* (also known as p21^cip1^), *CDKN1B* (also known as p27^kip1^), *CDKN2A* (also known as p16^ink4a^) and *CCL2* expression in late aPT and aTAL states. Furthermore, expression signatures for reparative processes in aEpi states were downregulated in the CKD (*n* = 28) over AKI (*n* = 22) cases used in this study (snCv3/scCv3; Supplementary Table 32). This is distinct from the immune response signatures that were more enriched in AKI cases more globally across cell types (Extended Data Fig. 12h and Supplementary Table 33). Overall, our findings are consistent with pro-inflammatory repair processes that may persist after injury^22^, or may subsequently transition to maladaptive or senescent pro-fibrotic states during disease progression.

## Discussion

In contrast to recent work to broadly integrate major healthy kidney cell types across disparate data modalities^49^, here we present a comprehensive spatially resolved healthy and injured single-cell atlas across the corticomedullary axis of the kidney. Signals between tubuli, stroma and immune cells that underlie normal and pathological cell neighbourhoods were identified, including putative adaptive or maladaptive repair signatures within the epithelial segments that may reflect a failure to complete differentiation and tubulogenesis. Spatial analyses identified that these epithelial repair states have elevated cytokine production, increased interactions with the distinct fibrotic and inflammatory cell types, and expression signatures linked to senescence and progression to end-stage kidney disease. Failure of these cells to complete tubulogenesis, which might arise from an incompatible cytokine milieu within the fibrotic niche, in itself might ultimately contribute to a progressive decline in kidney function. In turn, the high-cytokine-producing nature of these cells may further contribute to kidney disease through promotion of fibrosis. We portray a clear role for the relatively understudied TAL segment of the nephron, a region that is critical for maintaining osmotic gradient and blood pressure through tubuloglomerular feedback. The insights, discoveries and interactive data visualization tools provided here will serve as key resources for studies into normal physiology and sex differences, pathways associated with transitions from healthy and injury states, clinical outcomes, disease pathogenesis and targeted interventions.

## Methods

### Statistics and reproducibility

For 3D imaging and immunofluorescence staining experiments, each staining was repeated on at least two separate individuals or separate regions. For immunofluorescence validation studies, commercially available antibodies were used; 13 out of the 15 tissue samples were also analysed using snCv3 or scCv3. For ISH, 6 tissue samples (4 biopsies and 2 nephrectomies) were analysed. For Slide-seq, 67 tissue pucks (6 individuals) were analysed, with 2 individuals also analysed using snCv3 or Visium. For Visium, 23 kidney tissue sections (22 individuals) were imaged, including 6 that were also analysed using snCv3 or scCv3 and one examined using Slide-seq. Orthogonal validation of spatial transcriptomic annotations revealed similar marker gene expression in snCv3/scCv3 and these technologies, as well as spatial localization that corresponded with histologically validated Visium spot mapping. Although multiomic data from the same samples would be the most informative, this remains technically challenging. However, wherever possible, several technologies were performed on a subset of samples from the same patient and, in some cases, the same tissue block was used to generate multimodal data (Extended Data Fig. 1a and Supplementary Table 3). This heterogeneous sampling approach ensured cell type discovery while minimizing assay-dependent biases or artifacts encountered when using different sources of kidney tissue. We recognize that the heterogeneity of sample sources for several technologies is a potential limitation due logistics and limited patient biopsy material.

### Ethical compliance

We have complied with all ethical regulations related to this study. All experiments on human samples followed all relevant guidelines and regulations. Human samples (Supplementary Table 1) collected as part of the KPMP consortium (https://KPMP.org) were obtained with informed consent and approved under a protocol by the KPMP single IRB of the University of Washington Institutional Review Board (IRB 20190213). Samples as part of the HuBMAP consortium were collected by the Kidney Translational Research Center (KTRC) under a protocol approved by the Washington University Institutional Review Board (IRB 201102312). Informed consent was obtained for the use of data and samples for all participants at Washington University, including living patients undergoing partial or total nephrectomy or rejected kidneys from deceased donors. Cortical and papillary biopsy samples from patients with stone disease were obtained with informed consent from Indiana University and approved by the Indiana University Institutional Review Board (IRB 1010002261). For Visium spatial gene expression, reference nephrectomies and kidney biopsy samples were obtained from the KPMP under informed consent or the Biopsy Biobank Cohort of Indiana (BBCI)^50^ under waived consent as approved by the Indiana University Institutional Review Board (IRB 1906572234). Living donor biopsies as part of the HCA were obtained with informed consent under the Human Kidney Transplant Transcriptomic Atlas (HKTTA) under the University of Michigan IRB HUM00150968. Deidentified leftover frozen COVID-19 AKI kidney biopsies were obtained from the Johns Hopkins University pathology archive under waived consent approved by the Johns Hopkins Institutional Review Board (IRB 00090103).

### Single-cell and single-nucleus human tissue samples

For single-nucleus omic assays, tissues were processed according to a protocol available online (10.17504/protocols.io.568g9hw). For nucleus preparation, around 7 sections of 40 µm thickness were collected and stored in RNAlater solution (RNA assays) or kept on dry ice (accessible chromatin assays) until processing or used fresh. To confirm tissue composition, 5 µm sections flanking these thick sections were obtained for histology and the relative amount of cortex or medulla composition including glomeruli was determined. For single-cell omic assays, tissues used (15 CKD,12 AKI and 18 living donor biopsy cores) were preserved using CryoStor (StemCell Technologies).

### Single-cell, single-nucleus and SNARE2 RNA-seq, quality control and clustering

#### Isolation of single nuclei

Nuclei were isolated from cryosectioned tissues according to a protocol available online (10.17504/protocols.io.ufketkw) with the exception that 4′,6-diamidino-2-phenylindole (DAPI) was excluded from the nuclear extraction buffer and used only to stain a subset of nuclei used for counting. Nuclei were used directly for omic assays.

#### Isolation of single cells

Single cells were isolated from frozen tissues according to a protocol available online (10.17504/protocols.io.7dthi6n). The single-cell suspension was immediately transferred to the University of Michigan Advanced Genomics Core facility for further processing.

#### 10x Chromium v3 RNA-seq analysis

10x single-nucleus RNA-seq and 10x single-cell RNA-seq were performed according to protocols available online (10.17504/protocols.io.86khzcw and 10.17504/protocols.io.7dthi6n, respectively), both using the 10x Chromium Single-Cell 3′ Reagent Kit v3. Sample demultiplexing, barcode processing and gene expression quantifications were performed using the 10x Cell Ranger (v.3) pipeline using the GRCh38 (hg38) reference genome with the exception of a subset of scCv3 experiments that used hg19 (indicated in Supplementary Table 1). For single-nucleus data, introns were included in the expression estimates.

#### SNARE2 dual RNA and ATAC-seq analysis

SNARE-seq2^17^, as outlined previously^18^, was performed according to a protocol available online (10.17504/protocols.io.be5gjg3w). Accessible chromatin and RNA libraries were sequenced separately on the NovaSeq 6000 (Illumina) system (NovaSeq Control Software v.1.6.0 and v.1.7.0) using the 300 cycle and 200 cycle reagent kits, respectively.

#### SNARE2 data processing

Detailed step-by-step processing for SNARE2 data has been outlined previously^18^. This has now been developed as an automated data processing pipeline that is available at GitHub (https://github.com/huqiwen0313/snarePip). snarePip (v.1.0.1) was used to process all the SNARE2 datasets. The pipeline provides an automated framework for complex single-cell analysis, including quality assessment, doublet removal, cell clustering and identification, robust peak generation and differential accessible region identification, with flexible analysis modules and generation of summary reports for both quality assessment and downstream analysis. The directed acyclic graph was used to incorporate the entirety of the data-processing steps for better error control and reproducibility. For RNA processing, this involved removal of accessible chromatin contaminating reads using cutadapt (v.3.1)^51^, dropEst (v.0.8.6)^52^ to extract cell barcodes and STAR (version 2.5.2b)^53^ to align tagged reads to the genome (GRCh38). For accessible chromatin data, this involved snaptools (v.1.2.3)^54^ and minimap (v.2-2.20)^55^ for alignment to the genome (GRCh38).

#### Quality control of sequencing data

##### 10x snRNA-seq (snCv3)

Cell barcodes passing 10x Cell Ranger filters were used for downstream analyses. Mitochondrial transcripts (MT-*) were removed, doublets were identified using the DoubletDetection software (v.2.4.0)^56^ and removed. All of the samples were combined across experiments and cell barcodes with greater than 400 and less than 7,500 genes detected were retained for downstream analyses. To further remove low-quality datasets, a gene UMI ratio filter (gene.vs.molecule.cell.filter) was applied using Pagoda2 (https://github.com/hms-dbmi/pagoda2).

##### 10x scRNA-seq (scCv3)

As a quality-control step, a cut-off of <50% mitochondrial reads per cell was applied. The ambient mRNA contamination was corrected using SoupX (v.1.5.0)^57^. The mRNA content and the number of genes for doublets are comparatively higher than for single cells. To reduce doublets or multiplets from the analysis, we used a cut-off of >500 and <5,000 genes per cell.

##### SNARE2 RNA

Cell barcodes for each sample were retained with the following criteria: having an DropEst cell score of greater than 0.9; having greater than 200 UMI detected; having greater than 200 and less than 7,500 genes detected. Doublets identified by both DoubletDetection (v.3.0) and Scrublet (https://github.com/swolock/scrublet; v.0.2.2) were removed. To further remove low-quality datasets, a gene UMI ratio filter (gene.vs.molecule.cell.filter) was applied using Pagoda2.

##### SNARE2 ATAC

Cell barcodes for each sample that had already passed quality filtering from RNA data were further retained with the following criteria: having transcriptional start site (TSS) enrichment greater than 0.15; having at least 1,000 read fragments and at least 500 UMI; having fragments overlapping the promoter region ratio of greater than 0.15. Samples were retained only if they exhibited greater than 500 dual omic cells after quality filtering.

#### Clustering snCv3

Clustering analysis was performed using Pagoda2, whereby counts were normalized to the total number per nucleus, batch variations were corrected by scaling expression of each gene to the dataset-wide average. After variance normalization, all 5,526 significantly variant genes were used for principal component analysis (PCA). Clustering was performed at different *k* values (50, 100, 200, 500) on the basis of the top 50 principal components, with cluster identities determined using the infomap community detection algorithm. The primary cluster resolution (*k* = 100) was chosen on the basis of the extent of clustering observed. Principal components and cluster annotations were then imported into Seurat (v.4.0.0) and uniform manifold approximation and projection (UMAP) dimensionality reduction was performed using the top 50 principal components identified using Pagoda2. Subsequent analyses were then performed in Seurat. A cluster decision tree was implemented to determine whether a cluster should be merged, split further or labelled as an altered state. For this, differentially expressed genes between clusters were identified for each resolution using the FindAllMarkers function in Seurat (only.pos = TRUE, max.cells.per.ident = 1000, logfc.threshold = 0.25, min.pct = 0.25). Possible altered states were initially defined for clusters with one or more of the following features: low genes detected, a high number of mitochondrial transcripts, a high number of endoplasmic-reticulum-associated transcripts, upregulation of injury markers (*CST3*, *IGFBP7*, *CLU*, *FABP1*, *HAVCR1*, *TIMP2*, *LCN2*) or enrichment in AKI or CKD samples. Clusters (*k* = 100) that showed no distinct markers were assessed for altered-state features; if present, then these clusters were tagged as possible altered states, if absent then clusters were merged on the basis of their cluster resolution at *k* = 200 or 500. If this merging occurred across major classes (epithelial, endothelial, immune, stromal) at higher k values, then these clusters were instead labelled as ambiguous or low quality (including possible multiplets). For *k* = 100 clusters (non-epithelial only) that did show distinct markers, their *k* = 50 subclusters were assessed for distinct marker genes; if present, then these clusters were split further. The remaining split and unsplit clusters were then assessed for altered-state features. If present, they were tagged as possible altered states, if absent they were assessed as the final cluster. Annotations of clusters were based on known positive and negative cell type markers^11,12,58–60^ (Supplementary Table 5), the regional distribution of the clusters across the corticomedullary axis and altered state (including cell cycle) features. For separation of EC-DVR from EC-AEA, the combined population was independently clustered using Pagoda2 and clusters associated with medullary sampling were annotated as EC-DVR. For separation of the REN cluster, stromal cells expressing *REN* were selected on the basis of normalized expression values of greater than 3. Final overall assessment of clustering accuracy was performed using the Single Cell Clustering Assessment Framework (SCCAF v.0.0.10) using the default settings, and compared against that associated with broad cell type classifications (subclass level 1).

#### Annotating snCv3 clusters

To overcome the challenge of disparate nomenclature for kidney cell annotations, we leveraged a cross-consortium effort to use the extensive knowledge base from human and rodent single-cell gene expression datasets, as well as the domain expertise from pathologists, biologists, nephrologists and ontologists^11,12,22,58–61^ (see also Supplementary Tables 4 and 5 and the HuBMAP ASCT+B Reporter at GitHub (https://hubmapconsortium.github.io/ccf-asct-reporter)). This enabled the adoption of a standardized anatomical and cell type nomenclature for major and minor cell types and their subclasses (Supplementary Table 4), showing distinct and consistent expression profiles of known markers and absence of specific segment markers for some of the cell types (Extended Data Fig. 2a and Supplementary Table 5). The knowledge of the regions dissected and histological composition of snCv3 data further enabled stratification of distinct cortical and outer and inner medullary cell populations (Fig. 2b and Extended Data Fig. 1). The cell type identities and regional locations were confirmed through orthogonal validation using spatial technologies presented here and correlations with existing human or rodent stromal, immune, endothelial and epithelial datasets^4,25,58,59,61,62^ (Extended Data Fig. 2b–l).

### Atlas cell type resolution

Our atlas now includes a higher granularity for the loop of Henle, distal convoluted tubule and collecting duct segments, now resolving three descending thin limb cell types (DTL1, 2, 3); different subpopulations of medullary or cortical thick ascending limb cells (M-TAL/C-TAL); two types of distal convoluted tubule cells (DCT1, 2); intercalated and principal cells of the connecting tubules (CNT-IC and CNT-PC); cortical, outer medullary and inner medullary collecting duct subpopulations (CCD, OMCD, IMCD); and papillary tip epithelial cells abutting the calyx (PapE). Molecular profiles for rare cell types important in homeostasis were annotated, including juxtaglomerular renin-producing granular cells (REN); macula densa cells (MD); and a cell population with enriched Schwann/neuronal (SCI/NEU) genes *NRXN1*, *PLP1* and *S100B*. Major endothelial cell types were stratified, including endothelial cells of the lymphatics (EC-LYM) and vasa recta (EC-AVR, EC-DVR). Specific stromal and immune cell types were distinguished, including distinct fibroblast populations across the cortico-medullary axis and 12 immune cell types from lymphoid and myeloid lineages.

### Integrating snCv3 and SNARE2 datasets

Integration of snCv3 and SNARE RNA data was performed using Seurat (v.4.0.0) using snCv3 as reference. All counts were normalized using sctransform, anchors were identified between datasets based on the snCv3 Pagoda2 principal components. SNARE2 data were then projected onto the snCv3 UMAP structure and snCv3 cell type labels were transferred to SNARE2 using the MapQuery function. Both datasets were then merged and UMAP embeddings were recomputed using the snCv3 projected principal components. Integrated clusters were identified using Pagoda2, with the *k*-nearest neighbour graph (*k* = 100) based on the integrated principal components and using the infomap community detection algorithm. The SNARE2 component of the integrated clusters was then annotated to the most overlapping, correlated and/or predicted snCv3 cluster label, with manual inspection of cell type markers used to confirm identities. Integrated clusters that overlapped different classes of cell types were labelled as ambiguous or low-quality clusters. Segregation of EC-AEA, EC-DVR and REN subpopulations was performed as described for snCv3 above.

### Integrating snCv3 and scCv3 datasets

Integration of snCv3 and scCv3 data was performed using Seurat v.4.0.0 with snCv3 as a reference. All counts were normalized using sctransform, anchors were identified between datasets based on the snCv3 Pagoda2 principal components. scCv3 data were then projected onto the snCv3 UMAP structure and snCv3 cell type labels were transferred to scCv3 using the MapQuery function. Both datasets were then merged and UMAP embeddings recomputed using the snCv3 projected principal components. Integrated clusters were identified using Pagoda2, with the *k*-nearest neighbour graph (*k* = 100) based on the integrated principal components and using the infomap community detection algorithm. The scCv3 component of the integrated clusters was then annotated to the most overlapping or correlated snCv3 subclass, with manual inspection of cell type markers used to confirm identities. Cell types that could not be accurately resolved (PT-S1/PT-S2) were kept merged. Integrated clusters that overlapped different classes of cell types or that were too ambiguous to annotate were considered to be low quality and were removed from the analysis. Segregation of EC-AEA, EC-DVR and REN subpopulations was performed as described above.

### Assessment of snCv3, scCv3 and SNARE2 data integration

As described above, we used the demonstrated Seurat v.4.0.0 integration strategy^63^ to project query datasets (scCv3, SNARE2 RNA) into the same PCA space as our snCv3 reference. These imputed principal components were used to generate an integrated embedding and integrated clustering through Pagoda2. Query datasets within these integrated clusters were manually annotated on the basis of co-clustering with the reference data, predicted subclass levels and the manual inspection of marker genes. This process was necessary to account for misalignments that occurred for altered states showing more ambiguous marker gene expression profiles, especially for mapping between single-nucleus and single-cell technologies. To assess the accuracy in our alignments, we performed correlation of average expression signatures between the assigned query cell populations and the original reference cell populations (Extended Data Fig. 3e). Although several samples were examined using more than one platform (Supplementary Table 3 and Extended Data Fig. 1a), not all conditions could be covered by all technologies, with AKI/CKD biopsies too limited in size to process with SNARE2 and deeper medullary region capture being less likely for needle biopsies. Despite the differences in patient conditions and regions sampled, we were able to confirm cross-platform sampling with minimal batch contributions for a majority of our subclass (level 3) assignments (77 total). This was demonstrated through integrated bar plots for assay, patient, sex and condition contributions (Extended Data Fig. 3e). The degree to which cells/nuclei between assays were mixed within these subclasses was confirmed using normalized relative entropy weighted by subclass size^64^, with an average assay entropy across subclasses (covered by more than one technology) of 0.71 and an average patient entropy of 0.71 (out of 1). Mixing within the subclasses was also assessed on the cell embeddings (principal components) using the average silhouette width or ASW (scib.metrics.silhouette_batch function of the scIB package v.1.0.3^65^), with an average score of 0.86 for assays and 0.82 for patients (out of 1). Finally, the average of *k*-nearest neighbour batch effect test (kBET) score per subclass, computed for all patients using the scib.metrics.kBET function of the scIB package, was 0.49 (out of 1), which is consistent with other integration efforts^65^.

### Integrating snCv3 with published datasets

Integration with published data was performed using Seurat v.4.0.0 with snCv3 as a reference. All counts were normalized using sctransform, anchors were identified between datasets on the basis of the snCv3 Pagoda2 principal components. Published data were then projected onto the snCv3 UMAP structure and snCv3 cell type labels were transferred to the published dataset using the MapQuery function. Ref. ^12^ snDrop-seq data are available at the Gene Expression Omnibus (GEO: GSE121862). Ref. ^15^ single-nucleus RNA-seq and ref. ^14^ single-cell RNA-seq count matrices and metadata tables were downloaded from the UCSC Cell Browser (Cell Browser dataset IDs human-kidney-atac and kidney-atlas, respectively).

### NSForest marker genes

To identify a minimal set of markers that can identify snCv3 clusters and subclasses (subclass.l3), or scCv3 integrated subclasses (subclass.l3), we used the Necessary and Sufficient Forest^66^ (NSForest v.2; https://github.com/JCVenterInstitute/NSForest/releases/tag/v2.0) software using the default settings.

### Correlation analyses

For correlation of RNA expression values between snCv3 and scCv3, or SNARE2, average scaled expression values were generated, and pairwise correlations were performed using variable genes identified from Pagoda2 analysis of snCv3 (top 5,526 genes). For comparison with mouse single-cell RNA-seq data of healthy reference tissue^59^, raw counts were downloaded from the GEO (GSE129798). For comparison with mouse single-cell RNA-seq from IRI tissue^4^, raw counts were downloaded from the GEO (GSE139107). For human fibroblast and myofibroblast data^25^, raw counts were downloaded from Zenodo (10.5281/zenodo.4059315). For each dataset, raw counts were processed using Seurat: counts for all cell barcodes were scaled by total UMI counts, multiplied by 10,000 and transformed to log space. For comparison with mouse single-cell types of the distal nephron^61^, the precomputed Seurat object was downloaded from the GEO (GSE150338). For mouse bulk distal segment data^61^, normalized counts were downloaded from the GEO (GSE150338) and added to the ‘data’ slot in a Seurat object. Bulk-sorted immune cell reference data were obtained using the celldex package^67^ using the MonacoImmuneData()^62^ and ImmGenData()^67,68^ functions and log counts imported into the ‘data’ slot of Seurat. For correlation against these reference datasets, averaged scaled gene expression values for each cluster were calculated (Seurat) using an intersected set of variable genes identified for each dataset (identified using Padoda2 for snCv3 and Seurat for reference datasets). For immune reference correlations, a list of immune-related genes downloaded from ImmPort (https://immport.org) was used instead of the variable genes. Correlations were plotted using the corrplot package (https://github.com/taiyun/corrplot). Immune annotations within our atlas were further confirmed by manual comparison with recently reported data^14^.

### Cross-species alignment of cell types/states

For mouse single-nucleus RNA-seq data from IRI tissue^4^, raw counts were downloaded from the GEO (GSE139107). Integration was performed using Seurat v.4.0.0 with snCv3 as a reference. All counts were normalized using sctransform, anchors were identified between datasets on the basis of the snCv3 Pagoda2 principal components. Mouse data were then projected onto the snCv3 UMAP structure and snCv3 cell type labels were transferred using the MapQuery function.

### Computing single-nucleus/cell-level expression scores

To identify markers associated with altered states (degenerative; adaptive—epithelial or aEpi; adaptive—stromal or aStr; cycling), snCv3 and scCv3 data were independently used to identify differentially expressed genes between reference and corresponding altered states for each subclass level 1 (subclass.l1). To ensure general state-level markers, differentially expressed genes were identified using the FindConservedMarkers function (grouping.var = “condition.l1”, min.pct = 0.25, max.cells.per.ident = 300) in Seurat. A minimal set of general degenerative conserved genes was identified as enriched (*P* < 0.05) in the degenerative state of each condition.l1 (reference, AKI and CKD) and in at least 4 out of the 11 (snCv3) or 9 (scCv3) subclass.l1 cell groupings. A minimal set of conserved aEpi genes was identified as enriched (*P* < 0.05) in the adaptive state of each condition.l1 (reference, AKI and CKD) in both the aPT and aTAL cell populations. This aEpi gene set was then further trimmed to include only those genes that were enriched within the adaptive epithelial population (aPT/aTAL) versus all others using the FindMarkers function and with a minimum *P* value of 0.05 and average log_2_-transformed fold change of >0.6. A minimal set of conserved aStr genes was identified as enriched (*P* < 0.05) in the adaptive state of each condition.l1 (reference, AKI and CKD for snCv2; reference and AKI for scCv3) for stromal cells. To increase representation from MyoF in scCv3 showing a small number of these cells, MyoF-alone enriched genes (average log_2_-transformed fold change ≥ 0.6; adjusted *P* < 0.05) were included for the scCv3 gene set. The aStr gene sets were then further trimmed to include only those genes that were enriched within the adaptive stromal population (aFIB and MYOF) compared with all others using the FindMarkers function and with a minimum *P* value of 0.05 and average log_2_-transformed fold change of >0.6. A minimal set of cycling-associated genes was identified as those enriched (adjusted *P* < 0.05 and average log_2_-transformed fold change > 0.6) in the cycling state across all associated subclass.l1 cell groupings.

Scores for altered state, extracellular matrix and for gene sets associated with ageing or SASP were computed for each cell from averaged normalized counts using only the genes showing a minimum correlation to the averaged whole gene set of 0.1 (ref. ^25^) (https://github.com/mahmoudibrahim/KidneyMap). Ageing and SASP genes were obtained from ref. ^48^ (top 20 genes upregulated in ageing kidney)^48^, ref. ^69^ (genes from table S3, group.age A), ref. ^70^ (SASP genes from figure 2c) or ref. ^71^ (from table S1 (sheet IR Epithelial SASP), having a positive AVE log_2_ ratio)^71^.

### Gene set enrichment analyses (GSEA)

To compute gene set enrichments for aPT and aTAL, conserved genes differentially expressed in the adaptive over reference states were identified as indicated above. Gene set ontologies from the Molecular Signatures Database (MSigDB) were downloaded from https://gsea-msigdb.org and pathway enrichments were computed using fgsea^72^ and gage^73^, retaining only Gene Ontology terms that were significant (*P* < 0.05) for both. Redundant pathways were collapsed using the fgsea function collapsePathways and visualized using ggplot.

### SNARE2 accessible chromatin analyses

SNARE2 chromatin data were analysed using Signac^74^ (v.1.1.1). Peak calling was performed using the CallPeaks function and MACS (v.3.0.0a6; https://github.com/macs3-project/MACS) separately for clusters, subclass.l1 and subclass.l3 annotations. Peak regions were then combined and used to generate a peak count matrix using the FeatureMatrix function, then used to create a new assay within the SNARE2 Seurat object using the CreateChromatinAssay function. Gene annotation of the peaks was performed using GetGRangesFromEnsDb(ensdb = EnsDb.Hsapiens.v86). TSS enrichment, nucleosome signal and blacklist fractions were all computed using Signac. Jaspar motifs (JASPAR2020, all vertebrate) were used to generate a motif matrix and motif object that was added to the Seurat object using the AddMotifs function. For motif activity scores, chromVAR^75^ (v.1.12.0; https://greenleaflab.github.io/chromVAR) was performed using the RunChromVAR function. The chromVAR deviation score matrix was then added to a separate assay slot of the Seurat object. To assess the chromatin data, UMAP embeddings were computed from *cis*-regulatory topics that were identified through latent Dirichlet allocation using CisTopic^76^ (v.0.3.0; https://github.com/aertslab/cisTopic) and the runCGSModels function. Only regions accessible in 50 nuclei and nuclei with 200 of these accessible regions were used for cisTopic and downstream analyses. The UMAP coordinates for the remaining nuclei were added to the Seurat object. To ensure high-quality accessible chromatin profiles, only clusters with more than 50 nuclei were retained for downstream analyses (Supplementary Table 7). For joint embedding of SNARE2 accessible chromatin and gene expression, a weighted nearest-neighbour graph was computed using the FindMultiModalNeighbors function (Seurat) based on PCA (RNA) and latent semantic indexing or LSI (accessible chromatin) dimensionality reductions. UMAP dimensionality reduction was performed to visualize the joint embedding.

### DAR analyses

Sites that were differentially accessible for a given cell grouping (subclass) were identified against a selection of background cells with the best matched total peak counts, to best account for technical differences in the total accessibility for each cell. For this, the total peaks in each cell were used for estimation of the distribution of total peaks (depth distribution) for the cells belonging to the test cluster, and 10,000 background cells with a similar depth distribution to the test cluster were randomly selected. Differentially accessible sites (DARs) were then identified as significantly enriched in the positive cells over selected background cells using the CalcDiffAccess function (https://github.com/yanwu2014/chromfunks), where *P* values were calculated using Fisher’s exact tests on a hypergeometric distribution and adjusted *P* values (or *q* values) were calculated using the Benjamini–Hochberg method. For subclass level 2 DARs, VSM/P clusters were merged and the MD was combined with C-TAL before to DAR calling. Subclasses with >100 DARs with *q* < 0.01 were used for further analysis. Co-accessibility between all peak regions was computed using Cicero^77^ (v.1.8.1). Sites were then linked to genes on the basis of co-accessibility with promoter regions, occurring within 3,000 bp of a gene’s TSS, using the RegionGeneLinks function (https://github.com/yanwu2014/chromfunks) and the ChIPSeeker package^78^. DARs associated with markers for each subclass (identified at the subclass.l2 level using snCv3, *P* < 0.05) and showing *q* < 0.01 and a log-transformed fold change of >1 were selected for visualization. For this, DAR accessibility (peak counts) was averaged, scaled (trimming values to a minimum of 0 and a maximum of 5) and visualized using the ggHeat plotting function of the SWNE package^79^. Motif enrichment within cell type DARs was computed using the hypergeometric test (FindMotifs function) in Signac.

### Transcription factor analyses

To identify active transcription factors from SNARE2 accessible chromatin data, differential activities (or deviation scores) of TFBSs between different populations were assessed using the Find[All]Markers function through logistic regression and using the number of peak counts as a latent variable. Only transcription factors with expression detected within the corresponding cluster, subclass or state grouping were included. For PT and TAL clusters, TFBSs that were differentially active (*P* < 0.05, average log_2_-transformed fold change of >0.35) and associated with transcription factors with expression detected in at least 2.5% of nuclei (SNARE2) were identified between clusters. Common aPT/aTAL TFBS activities were identified from an intersection of those differentially active and expressed within adaptive PT and TAL clusters. For aPT and aTAL trajectory modules, TFBSs showing differential activity between modules (adjusted *P* < 0.05, average log_2_-transformed fold change of >0.35) and expression detected within at least 2.5% of nuclei/cells (snCv3/scCv3) were identified. For common degenerative state TFBS activities, differentially active TFBSs were identified between reference and degenerative states for each level 1 subclass. Significant degenerative state TFBS activities (*P* < 0.05, average log_2_-transformed fold change of >0.35) in three or more subclass.l1 were trimmed to those showing expression detected in more than 20% of the degenerative state nuclei/cells for snCv3/scCv3.

### Ligand–receptor interaction analyses

Ligand–receptor analyses were performed on the basis of the CellChat package (v.1.0.0; https://github.com/sqjin/CellChat). Only cells in TAL, immune and stroma of subclass level 2 (immune: cDC, cycMNP, MAC-M2, MAST, MDC, N, ncMON, NKT, pDC, PL, T and B; stroma: MyoF, FIB, dFIB, cycMyoF and aFIB) and interactions for secreted ligands were used to infer the cell–cell communication. For cells in the TAL trajectory, we computed the intercellular cell communication probability between each module and other cell populations using the CellChat function computeCommunProb (see ref. ^80^ for a detailed description of the method). The overall scaled communication probability was then visualized based on a circle plot using a customized plot_communication function (Code availability). To further understand which signals contribute most to the ligand–receptor (LR) interaction pathways, we generated the pathway enrichment heat map of each interaction for incoming, outgoing and overall signals using the plotSigHeatmap function (Code availability). The contribution of significant LR pairs of each interaction was also identified using netAnalysis_contribution in the CellChat package.

### GWAS analyses

To link SNARE2 cell types to kidney genome-wide association study (GWAS) traits and diseases, we first summed the binary peak accessibility profiles for all cells belonging to the same cell type to create a pseudobulk peak-by-subclass accessibility matrix. Pseudobulk analyses give more stable results, especially as SNARE2 accessibility data can be sparse. To ensure sufficient coverage, we used subclass level 2 groupings with the following modifications: VSM/P clusters were merged; MD was combined with C-TAL; subclasses with <100 DARs with *q* < 0.01 were excluded. We used g-chromVAR^81^ (v.0.3.2), an extension of chromVAR for GWAS data, to identify cell types with higher than expected accessibility of genomic regions overlapping GWAS-linked single-nucleotide polymorphisms (SNPs). Running g-chromVAR requires first identifying GWAS-linked SNPs that are more likely to be causal, a process known as fine-mapping. For the chronic kidney failure GWAS traits, we used existing fine-mapped SNPs from the CausalDB database, using the posterior probabilities generated by CAVIARBF^82,83^. The original GWAS summary statistics files were obtained from an atlas of genetic associations from the UK BioBank^84^. We manually fine-mapped the CKD, eGFR, blood urea nitrogen and gout traits using the same code that was used to generate the CausalDB database (https://github.com/mulinlab/CAUSALdb-finemapping-pip). The summary statistics for all of these traits are available at the CKDGen Consortium site (https://ckdgen.imbi.uni-freiburg.de/)^85,86^. We also manually fine-mapped the hypertension trait and the original summary statistics can be found on the EBI GWAS Catalog^87^. We looked only at causal SNPs with a posterior causal probability of at least 0.05 to ensure that SNPs with low causal probabilities did not cause false-positive signals. Moreover, as g-chromVAR selects a semi-random set of peaks with similar average accessibility and GC content as background peaks, the method has an element of randomness. To ensure stable results, we ran g-chromVAR 20 times and averaged the results. Cluster/trait *z*-scores were plotted using ggheat (https://github.com/yanwu2014/swne).

To link causal SNPs to genes, we used functions outlined in the chromfunks repository (https://github.com/yanwu2014/chromfunks; /R/link_genes.R). This involved the identification of causal peaks for each cell type and trait (minimum peak *Z* score of 1, minimum peak posterior probability score of 0.025). Sites were then linked to genes on the basis of co-accessibility (Cicero) with promoter regions, occurring within 3,000 bp of a gene’s TSS. Only sites associated with genes detected as expressed in 10% of TAL nuclei/cells (snCv3/scCv3) were included. Motif enrichment within the causal SNP and TAL-associated peaks was performed using the FindMotifs function in Seurat and only motifs for transcription factors expressed in 10% of TAL nuclei/cells (snCv3/scCv3) were included (Supplementary Table 31). For a TAL-associated ESRRB transcription factor subnetwork, peaks were linked to genes using Cicero, then subset to those associated with TAL (C-TAL, M-TAL) marker genes that were identified using the Find[All]Markers function in Seurat for subclass.l3 (*P* < 0.05). Transcription factors were then linked to gene-associated peaks on the basis of the presence of the motif and correlation of peak and TFBS co-accessibility (chromVAR), using a correlation cut-off of 0.3. Only transcription factors with expression detected within 20% of TAL cells or nuclei (snCv3/scCv3) were included. Eigenvector centralities were then computed using igraph and the transcription-factor-to-gene network was visualized using PlotNetwork in chromfunks.

### Disease-associated gene set enrichment analyses

Genes linked with CKDGen consortium GWAS loci for the kidney functional traits eGFR and urinary albumin-creatinine ratio (UACR) were obtained from table S14 of ref. ^88^. These included the top 500 genes per trait or only those genes also implicated in monogenic glomerular diseases. eQTLs associated with eGFR, systolic blood pressure and general kidney function were obtained from tables S20, S21 and S22 of ref. ^89^, respectively. Genes associated with the transition from acute to chronic organ injury after ischaemia–reperfusion were obtained from ref. ^90^ from the following supplementary tables: Acute_Human_Specific (table S3, Human specific column); Acute_Mouse_Overlap (table S3, Shared column); Mid_Acute (table S8, cluster 2 genes); Late_Human_Specific (table S9, Human specific column); Late_Mouse_Overlap (table S9, Shared column); Late_Fibrosis (table S6, positive logFC); Late_Recovery (table S6, negative logFC). Each gene set was assessed for its enrichment within combined snCv3 and scCv3 subclass (level 3) differentially expressed genes (adjusted *P* < 0.05, log-transformed fold change of >0.25). Enrichments were performed using Fisher’s exact tests and the resultant −log_10_[*P*] values were scaled and visualized using ggplot2.

### Patient cohorts used for clinical association analyses

NEPTUNE^91^ (193 adult patients) and ERCB^45^ (131 patients) expression data were used as validation cohorts to determine the significance between patients with different levels of cell state gene expression. NEPTUNE (NCT01209000) is a multicentre (21 sites) prospective study of children and adults with proteinuria recruited at the time of first clinically indicated kidney biopsy (Supplementary Table 34). The study participants were followed prospectively, every 4 months for the first year, and then biannually thereafter for up to 5 years. At each study visit, medical history, medication use and standard local laboratory test results were recorded, while blood and urine samples were collected for central measurement of serum creatinine and urine protein/creatinine ratio (UPCR) and eGFR (ml per min per 1.73 m^2^). End-stage kidney disease (ESKD) was defined as initiation of dialysis, receipt of kidney transplant or eGFR <15 ml per min per 1.73 m^2^ measured at two sequential clinical visits; and the composite end point of kidney functional loss by a combination of ESKD or 40% reduction in eGFR^92^. Genome-wide transcriptome analysis was performed on the research core obtained at the time of a clinically indicated biopsy using RNA-seq by the University of Michigan Advanced Genomics Core using the Illumina HiSeq2000 system. Read counts were extracted from the fastq files using HTSeq (v.0.11). NEPTUNE mRNA-seq data and clinical data are controlled access data and will be available to researchers on request to NEPTUNE-STUDY@umich.edu.

ERCB is the European multicentre study that collects biopsy tissue for gene expression profiling across 28 sites. Transcriptional profiles of biopsies from patients in the ERCB were obtained from the GEO (GSE104954).

### Clinical association of cell state scores

The gene expression data from the tubulointerstitial compartment of the kidney biopsies from NEPTUNE patients was used to calculate the composite scores for the genes enriched in degenerative, aPT, aTAL and aStr states. The expression of the genes that were uniquely enriched in the cell state (described above) and that were found in both snCv3 and scCv3 were used to calculate the composite cell state score (Supplementary Table 29). As scCv3 did not efficiently identify all stromal cell types, the union of the enriched genes from scCv3 and snCv3 data were used to calculate the aStr cell state score. We also generated a cell state score for the genes that were commonly enriched in aPT and aTAL cells (common).

For outcome analyses (40% loss of eGFR or ESKD) in the NEPTUNE cohort, patient profiles were binned according to the degree of cell state score by tertile. Clinical outcomes were available on 193 participants with a total of 30 events. Kaplan–Meier analyses were performed using log-rank tests to determine significance between patients in different tertiles of cell state gene expression. Moreover, for the different cell state scores, multivariable adjusted Cox proportional hazard analyses were performed using five statistical models adjusting for different sets of potential confounding effects given the overall few number of events: (1) age, sex and race; (2) baseline eGFR and UPCR; (3) immunosuppressive treatment and FSGS status; (4) eGFR, UPCR and race self-reported as Black (factors that were associated with outcome in this dataset); and (5) immunosuppressive treatment, eGFR and UPCR (Supplementary Table 30). Note that the adjusted models simply assess whether the association with outcome persists after adjusting for common clinical features (that is, confounding effects), but do not assess for prediction accuracy.

In the ERCB cohort, differential expression analyses using multivariable regression modelling were performed between the cell state scores in the disease groups and living donors. Age and sex were used as covariates. The cell state scores for both NEPTUNE and ERCB bulk mRNA transcriptomics data were generated^93^. In brief, the cell state scores were generated by creating *Z* scores for each of the cell state gene sets and then using the average *Z* score as the cell state composite score. These analyses found scores for all adaptive epithelial, but not degenerative, states were significantly higher in the patients with diabetic nephropathy patients compared to that of living donors (Supplementary Table 30). After adjusting for sex and age, both aPT and aTAL were significant when scores from patients with diabetic nephropathy were compared with those of living donors and aPT scores were significant even after correcting for the different disease groups.

### Sample-level analysis and clustering on clinical association gene sets

To find association of patients based on altered-state gene signatures that were used in clinical association analyses (Supplementary Table 30), we performed sample-level clustering. All of the cells from the same patient in snCv3 and scCv3 were aggregated to get pseudo-bulk count matrices on the basis of the associated clinical gene set. The matrices were further normalized by RPKM followed by *t*-distributed stochastic neighbour embedding (*t*-SNE) dimensionality reduction. Groups of patients were then identified based on *k*-means clustering and density-based spatial clustering (DBSCAN) methods in the reduced space. To associate the patient clusters with clinical features, we calculated the distribution of eGFR in each identified group (Code availability).

To identify gene sets that best differentiate between AKI and CKD patients in the PT and TAL cell populations, we trained a gene-specific logistic regression model based on the sample-level gene expression. The model was used to assess the predictive power that differentiate patients with AKI and CKD in both snCv3 and scCv3 measured by area under the curve (AUC). The genes with AUC > 0.65 on both snCV3 and scCv3 were selected for downstream analysis (Supplementary Table 32).

To identify genes that were differentially expressed between AKI and CKD across all cell types, we aggregated the cells associated with each subclass (level 1) to generate cell-type-specific pseudocounts for each sample and performed differential gene expression analysis based on the DEseq2 method using the estimatePerCellTypeDE function in the Cacoa package (v.0.2.0; https://github.com/kharchenkolab/cacoa).

### Pseudotime analysis of PT and TAL cells

To find cells associated with disease progression, we performed trajectory analysis for PT and TAL cells. To get accurate pseudotime and trajectory estimation, we removed degenerative cell populations in both PT and TAL and inferred the trajectory for single nuclei and single cells separately using the Slingshot package^94^ (v.2.0.0). We specified normal cell populations as the end points for trajectory inference (S1–S3 in PT and M-TAL in TAL) using the Slingshot parameter end.clus. The correspondent trajectory embedding was visualized using the plotEmbedding function in the Pagoda2 package.

To identify whether the gene expression was statistically significantly associated with the inferred trajectory, we modelled the expression of a gene as a function of the estimated pseudotime by fitting a gam model with cubic spline regression using formula exp_*i*_ = *f*(*t*) + *ϵ*, where *t* is the pseudotime and *f* is the function of cubic spline. The model is then compared to a reduced model exp_*i*_ = *f*(1) + *ϵ* to get *P*-value estimates using the *F*-test. The Benjamin–Hochberg method was used to calculate the adjusted *P* values. To further identify candidate genes showing potential differences between patients with AKI and CKD, we extended the base gam model by fitting a conditional-smooth interaction using CKD as a reference.

### Gene module detection and cell assignment

To identify expression modules for significant gene sets along the estimated trajectories, we applied the module detection algorithm implemented in the WGCNA package^95^ (v.1.70-3) based on the smoothed gene expression matrix with parameters softPower = 10 and minModuleSize = 20. The similar modules detected by the original parameters were further merged. In total, we identified five different modules in PT and six modules in TAL cells. For the gene sets in each module, we further performed pathway analysis using the Reactome online tool^96^ (https://reactome.org/PathwayBrowser/). The enrichment of clinical associated gene sets for each module (Fig. 6e) was assessed by performing log ratio enrichment tests. To predict the transcription factor activities of PT and TAL subclass genes, we used the DoRothEA package (v.1.7.2) as targets. DoRothEA transcription factors and transcriptional targets were curated from both human and mouse evidence. The transcription factor activity scores for each cell type were calculated based on the run_viper function of the viper package (v.3.15; https://bioconductor.org/packages/release/bioc/html/viper.html).

To identify cells that are associated with each module, we developed a systematic approach. In brief, for the cells in the smoothed expression matrix, we performed dimension reduction using PCA followed by Louvain clustering. This enabled the identification of cell clusters along the trajectory. For the identified cell clusters, we then performed hierarchical clustering to calculate the correlation of each module on the basis of mean gene expression values and further linked the clusters with associated modules by cutting the hierarchical tree. Finally, module labels for each cell were assigned on the basis of its associated clusters. To link single-cell datasets with single-nucleus modules, we performed *k*-means clustering based on the joint embedding of PT or TAL cells and assigned the cells in scCv3 to modules on the basis of the majority voting from its *k*’s nearest neighbours (Code availability).

To further investigate cluster-free compositional change between disease conditions, we also performed cell density analysis, in which we compared the normalized cell density between AKI and CKD conditions through 2D kernel estimates using Cacoa Package. *Z* scores were calculated to identify the regions that showed significant differences in cell density.

To validate the direction of modules inferred from human data, we performed joint alignment of the human and mouse trajectories. The individual trajectories inferred separately from these two species (Slingshot, described above) were aligned to generate a joint trajectory using CellAlign (https://github.com/shenorrLab/cellAlign) with parameters winSz = 0.1 and NumPts = 1000. The collection groups (timepoints from injury) derived from mouse data were then projected to human cells based on the joint trajectory. The genes that were conserved or divergent between the two species were specified as overlapping/distinct gene sets that were tested for significance based on a gam model inferred from the trajectory (see above).

### RNA velocity analyses

Spliced and unspliced reads were counted from Cell Ranger BAM files for each snCv3 run using velocyto^97^ (v.0.17.17) and using the GRCh38 gene annotations prepackaged with the Cell Ranger pipeline. Repetitive elements were downloaded from the UCSC genome browser and masked from these counts. Corresponding loom files were loaded into R using the SeuratWrappers function ReadVelocity and converted to Seurat objects using the as.Seurat function. aPT or aTAL trajectory populations were then subset and RNA velocity estimates were calculated using scVelo^98^ (v.0.2.4) through a likelihood-based dynamical model. Velocity embeddings on the trajectory UMAPs were visualized using the pl.velocity_embedding_stream function. Latent times based on transcriptional dynamics predicted from splicing kinetics were computed and the top 300 dynamical genes were plotted using the pl.heatmap function. Top likelihood genes were computed for each TAL module to identify potential drivers for these states. Spliced versus unspliced or latent time scatter plots were generated using the pl.scatter function.

### GRN analyses

GRNs associated with TAL trajectory modules were constructed using Celloracle (v.0.9.1) with the default parameters outlined in the provided tutorials (https://morris-lab.github.io/CellOracle.documentation). The base GRN was first constructed from SNARE2 accessible chromatin data. Co-accessible peaks across cell types identified using Cicero (v.1.8.1) were linked to genes through their TSS peaks to identify accessible promoter/enhancer DNA regions. Peaks were then scanned for transcription-factor-binding motifs (gimme.vertebrate.v5.0) to generate a base GRN. snCv3 data were then used to identify TAL state-specific GRNs. To ensure that relevant genes were used, we included genes that varied across the aTAL trajectory (Supplementary Table 17), showed dynamic module-specific transcription from scVelo analyses (Supplementary Table 21), were variably expressed across TAL cells (Pagoda2) or that were associated with differential transcription factor activities (Supplementary Table 20). GRN inference through regularized machine learning regression models was performed to prune inactive (insignificant or weak) connections and to select active edges associated with regulatory connections within each module or state, retaining the top 2,000 edges ranged by edge strength. Network scores for different centrality metrics were then calculated and visualized using Celloracle plotting functions. For in silico transcription factor perturbation analyses, target gene expression was set to 0 and resultant gene expression values were extrapolated or interpolated using the default parameters of Celloracle and according to the provided tutorial. Stromal GRN construction was performed as indicated above, except using a gene subset that included variable STR genes identified using Pagoda2; subclass level 3 markers for FIB, aFIB, MyoF (adjusted *P* < 0.05); or transcription factors with expression detected in at least 2.5% of nuclei (SNARE2) and having binding sites that were differentially active between STR subclasses (*P* < 0.05). To ensure BMP target SMADs were represented, SMAD1/5/8 were also included.

### SLIDE-seq2

#### Puck preparation and sequencing

Tissue pucks were prepared from fresh frozen kidney tissue either embedded in Optimal Cutting Temperature (OCT) compound or frozen in liquid nitrogen and sequenced^20,99^ according to a step-by-step protocol (10.17504/protocols.io.bvv6n69e). Libraries were sequenced on the NovaSeq S2 flowcell (NovaSeq 6000) with a standard loading concentration of 2 nM (read structure: read 1, 42 bp; index 1, 8 bp; read 2, 60 bp; index 2, 0 bp). Demultiplexing, genome alignment and spatial matching was performed using Slide-seq tools (https://github.com/MacoskoLab/slideseq-tools/releases/tag/0.1).

#### Deconvolution

We used Giotto^100^ (v.1.0.3) for handling the slide-seq data and RCTD^101^ (v.1.2.0) for the cell type deconvolution. As only reference tissue was used for slide-seq, all degenerative states as well as PapE, NEU, B and N were removed from the snCv3 Seurat object prior to deconvolution. The Seurat object was randomly subsampled to have at most 3,000 cells from each level 2 (l2) subtype and the level 1 (l1) subclasses of ATL and DTL were merged. For each data source, that is, HuBMAP or KPMP (Supplementary Table 2), the counts from all beads across all pucks were pooled and deconvolved hierarchically: first, beads with less than 100 UMIs and genes detected in less than 150 beads were removed. Then, the broad l1 subclass annotations in the Seurat object were used to deconvolve all beads (gene_cutoff = 0.0003, gene_cutoff_reg = 0.00035, fc_cutoff = 0.45, fc_cutoff_reg = 0.6, manually adding *REN* in the RCTD gene list and merging ATL and DTL subtypes as TL). The prediction weights were normalized to sum to 100 per bead. Beads for which one cell type had a relative weight of 40% or higher were classified as that l1 subclass. Then, for each l1 subclass, all classified beads were further deconvolved using the l2 annotation of that subclass, as well as the remaining subclass l1 annotations (same parameters as l1). Note that, for each l2 deconvolution, the bulk parameters in RCTD were fitted using all beads and then the RCTD object was subsetted to only contain the selected beads for the l2 deconvolution. Classification at subclass l2 was done similar to l1 with the maximum relative weight cut-off of 30% or 50% depending on the stringency needed for an analysis (50% for Figs. 2c,f and Extended Data Fig. 4b and 30% in other analyses). For plotting gene counts, the scaling was performed with the command normalizeGiotto(gObj, scalefactor = 10000, log_norm = T, scale_genes = T, scale_cells = F). The marker gene dot plots were plotted using the DotPlot function in Seurat (v.4.0.0).

#### Cell type interaction

Coarse cell–cell interactions can be revealed by looking for cell types that tend to be in close proximity. For each puck, we created a neighbourhood graph based on Delaunay triangulation in which each bead is connected by an edge to at least one other neighbouring bead, provided that their distance is smaller than 50 µm. For each pair of cell types, we count the number of times they are connected by edges. Then, the cell type labels are randomly permuted 2,500 times to form the null distribution of the number of connections. The expected number of connections between pairs of cell types is calculated from this simulation and the proximity enrichment is defined as the ratio of the observed over the expected frequency of connections. The network construction and enrichment analysis were performed using Giotto’s createSpatialNetwork and cellProximityEnrichment commands, respectively. Those beads with maximum level 2 weight less than 30% were removed. We further excluded spurious beads that were outside of the visual boundary of the tissue (only for the pucks of which the names start with ‘Puck_210113’) by manually specifying straight lines that follow the tissue boundary. For cortical pucks (Supplementary Table 2), M-PC, C-PC and IMCD labels were relabelled as PC; M-TAL and C-TAL as TAL; and EC-DVR was merged into EC-AEA. Other medullary and cycling subtypes were removed. For medullary pucks, M-PC and C-PC were relabelled as PC; M-TAL and C-TAL as TAL; all DTL subtypes as DTL; and EC-AEA was merged into EC-DVR. Other cortical and cycling subtypes were removed.

To generate the proximity plots in Extended Data Fig. 4, the enrichment values for each cell type pair were averaged across all pucks from the same region and plots were generated using the R package ggGally (v.2.1.2). For the cortex and medulla, respectively, only the connections with mean enrichment values higher than 0.7 and 0.8 were plotted.

### 10x Visium spatial transcriptomics

#### Preparation, imaging and sequencing

Human kidney tissue was prepared and imaged according to the Visium Spatial Gene Expression (10x Genomics) manufacturer’s protocol (CG000240, Visium Tissue Preparation Guide) and as previously described^102^. Nephrectomy (*n* = 6), AKI (*n* = 6) and CKD (*n* = 11) samples were sectioned at 10 µm thickness from OCT-compound-embedded blocks. These 23 samples represent 22 participants because 2 samples (1 cortex and 1 medulla) were obtained from the same participant with CKD. A Keyence BZ-X810 microscope equipped with a Nikon ×10 CFI Plan Fluor objective was used to acquire H&E-stained bright-field mosaics, which were subsequently stitched. mRNA was isolated from stained tissue sections after permeabilization for 12 min. Released mRNA was bound to oligonucleotides in the fiducial capture areas. mRNA was then reverse-transcribed and underwent second strand synthesis, denaturation, cDNA amplification and SPRIselect cDNA cleanup (Visium CG000239 protocol) as part of library preparation. Sequencing was performed on the Illumina NovaSeq 6000 system^103^.

#### Gene expression analysis

Space Ranger (v.1.0 or higher) with the reference genome GRCh38 was used to perform expression analysis, mapping, counting and clustering. Summary statistics and quality-control metrics are included in Extended Data Fig. 5 and Supplementary Table 2. Normalization was performed using SCTransform^104^. Final data processing was performed in Seurat (v.3.2.3). Expression feature plots depict the intensity of transcript expression in each spot. In each Visium sample, the outermost layer of spots was eliminated from comparative analyses if the edge was manually cut by a razor.

#### Deconvolution

Using Seurat (v.3.2.0), a transfer score system was used to assess and map the proportion of signatures arising from each 55 µm spot. The transfer score reflects a probability between each spot’s signature and its association with a given snCv3 subclass (level 2). The highest probability transfer scores have the highest proportion mapped within each spatial transcriptomics spot pie graph. For cell type feature plots (Figs. 2g and 3f and Extended Data Fig. 7i), subclass level 2 cell type transfer scores were mapped to convey the proportion of signature underlying each spot. For cell state feature plots (Fig. 3b), instead of mapping subclass level 2 cell types, the aEpi cell state annotated in snCv3 was mapped across all spots in the samples. We summed the proportion of signatures arising from all cell types corresponding to each of the 6 cell states in all spots of all samples (Fig. 3a). The proportions of cell state were compared across nephrectomy, AKI and CKD samples using Fisher’s exact tests.

#### Colocalization of epithelial, immune and stromal cells

In all spots across all samples, we categorized spots into healthy, adaptive or degenerative epithelial cell states on the basis of the highest proportion of epithelial cell state signature as calculated in Fig. 3a. For stromal or immune cell type colocalization, we first selected spots with non-zero transfer scores of each cell type in all 23 samples. The presence of stromal or immune cell signature was considered colocalized with an epithelial cell if its stromal or immune transfer score exceeded its mean transfer score across all selected spots. An odds ratio was calculated for colocalization between the healthy, adaptive and degenerative epithelial cell state with stromal or immune cell signature.

#### Cell state marker expression

To compare marker gene expression associated with the healthy, adaptive and degenerative cell states (Fig. 3d), we first categorized a subset of spots from AKI and CKD samples into 1 of 5 predominant cell types: POD, PT, TAL, CD or FIB. For the PT, TAL and fibroblasts, a spot was selected if the highest proportion of its signature (level 1 mapping) corresponded to one of these cell types. For the CD subset, a spot was selected if the sum of level 1 mapping proportions for the PC and IC contributed most to its signature. POD spots were defined by the presence of a minimum of 20% signature arising from the level 1 POD label. Once the subsets of PT, TAL, fibroblast, CD and POD spots were selected, each spot was further divided into healthy, adaptive or degenerative cell state groups based on the highest proportion of cell state signature as calculated in Fig. 3a. For PODs, the presence of EC-GC signature was considered to be a degenerative equivalent given that a loss of POD markers was associated with an observed gain in EC-GC signatures within DKD samples.

#### Niche analysis

To examine the diversity of cell types colocalizing with TAL epithelial cells, we selected spots with more than 20% TAL signature and in which the highest proportion of signature arose from level 1 TAL mapping. Using Seurat clustering methodology, selected spots were reclustered after Seurat label transfer scores were substituted in lieu of gene expression. Spots with similar proportions of signature arising from TAL cell types and states, stromal cells and immune cells were clustered into 13 niches. Niches were mapped over the 23 kidney samples and the marker gene expression in each niche was determined. To depict the relative proportion of each cell type, the transfer score average was first computed in each niche. Next, a *z* score for each cell type was calculated across the niches.

#### Histological validation

To determine whether the 74 snCv3 subclasses (level 2) were appropriately mapped to histological structures, the proportion of signature in each spot was compared to a histologically validated set of six unsupervised clusters defined by Space Ranger^102^ (Extended Data Fig. 5a). These six unsupervised clusters (glomerulus, PT, loop of Henle, distal convoluted tubule, connecting tubule and collecting duct, and the interstitium) had an overall alignment of 97.6% with the underlying histopathologic structures in the H&E image. In each sample, regions of cortex and medulla were defined by histological evaluation, including the presence of glomeruli. Of the 23 samples, 18 samples were composed of only cortex, 4 samples were a combination of cortex and medulla and 1 sample was completely medulla.

### Label-free and multifluorescence large-scale 3D imaging

Kidney biopsy cores frozen in OCT from patients with AKI or CKD enrolled in KPMP were used for label-free imaging followed by multiplexed-fluorescence large-scale 3D imaging as outlined in the protocol (10.17504/protocols.io.9avh2e6) and described in a recent publication^27^. Frozen biopsies were sectioned to a thickness of 50 µm using a cryostat and then immediately fixed in 4% fresh paraformaldehyde (PFA) for 24 h and subsequently stored at 4 °C in 0.25% PFA.

The first step in imaging consists of label-free imaging with multiphoton microscopy to collect autofluorescence and second harmonic images of the unlabelled tissue mounted in non-hardening mounting medium. Imaging was conducted using a Leica SP8 confocal scan-head mounted to an upright DM6000 microscope. For large-scale imaging of tissues at the sub-micrometer resolution, the Leica Tile Scan function was used to collect a mosaic of smaller image volumes using a high-power, high-numerical aperture objective. Leica LASX software (v.3.5) was then used to stitch these component volumes into a single image volume of the entire sample. The scanner zoom and focus motor control were set to provide voxel dimensions of 0.5 × 0.5 μm laterally and 1 μm axially.

Labelling of tissue for fluorescence microscopy was preceded by washing in phosphate-buffered saline (PBS) and blocking with PBS with 0.1% Triton X-100 (MP Biomedical) and 10% normal donkey serum (Jackson Immuno Research). Antibodies for indirect immunofluorescence were applied first for 8–16 h at room temperature, followed by washing cycles of PBS with 0.1% Triton X-100. An incubation cycle with secondary antibodies occurred next, followed by washing and finally application of directly labelled antibodies. Antibodies targeting markers for tubular cells and structures (aquaporin-1, uromodulin, F-actin) and immune cells (myeloperoxidase, CD68, CD3, siglec 8) were used, in addition to nuclei labelling using DAPI (Supplementary Table 35). After the final washing cycles, the tissue was mounted in Prolong Glass (Thermo Fisher Scientific).

Confocal microscopy was conducted using a Leica ×20/0.75 NA multi-immersion objective (adjusted for oil immersion), with excitation sequentially provided by a solid-state laser launch with laser lines at 405 nm, 488 nm, 552 nm and 635 nm. Images in 16 channels (emission spectra collected by PMT detectors adjusted for the following ranges: 410–430 nm, 430–450 nm, 450–470 nm, 470–490 nm, 500–509 nm, 510–519 nm, 520–530 nm, 530–540 nm, 570–590 nm, 590–610 nm, 610–630 nm, 631–651 nm, 643–664 nm, 664–685 nm, 685–706 nm and 706–726 nm) were collected for each focal plane of each panel of the 3D mosaic. The resulting 16-channel image was then spectrally deconvolved (by linear unmixing using the Leica LASX linear unmixing software) to discriminate the eight fluorescent probes in the sample. Validation of the linear unmixing was described previously^27^.

### Confocal immunofluorescence microscopy

Human kidney tissue samples from the cortex or medulla were fixed in 4% PFA, cryopreserved in 30% sucrose and frozen in OCT cryomolds, and were cut into 5 μm sections. The sections were post-fixed with 4% PFA for 15 min at room temperature, blocked in blocking buffer (1% BSA, 0.2% skimmed milk, 0.3% Triton X-100 in 1× PBS) for 30 min at room temperature and then immunofluorescence microscopy was performed, first by overnight incubation at 4 °C with primary antibodies, followed by labelling with secondary antibodies. The primary antibodies included NRXN-1β, TUJ1, collagen I and III, synapsin-1, NPSH-1, SLC14A2, UMOD, CD31, CD34, CD11b, PROM1, KIM1, VCAM1, AQP1, AQP2, CD45 and S100 (Supplementary Table 36). After washing, labelling with the secondary antibodies was performed using Alexa-488-conjugated goat anti-mouse IgG, Cy3-conjugated goat anti-rabbit IgG or Cy5-conjugated donkey anti-goat IgG at room temperature for 1 h. After washing, the sections were counterstained with DAPI for nuclear staining. Images were acquired with a Nikon 80i C1 confocal microscope.

### In situ hybridization

Human kidney tissues were sectioned with 3 μm from formalin-fixed, paraffin-embedded (FFPE) blocks. In situ detections of PROM1, CST3 and EGF mRNA transcripts were performed with the use of RNAscope Probes Hs-PROM1 (311261, Advanced Cell Diagnostics), Hs-CST3 (528181, Advanced Cell Diagnostics), and Hs-EGF (605771, Advanced Cell Diagnostics) and RNAscope kit (322330, Advanced Cell Diagnostics) according to the manufacturer’s protocol. RNAscope Positive Control Probe Hs-UBC (310041, Advanced Cell Diagnostics) was used as a positive control. A horseradish-peroxidase-based signal amplification system (322310, RNAscope 2.0 HD Detection Kit-Brown, Advanced Cell Diagnostics) was used to hybridize with target probes followed by DAB staining. The sections were then counterstained with haematoxylin (3535-16, RICCA Chemical Company). Positive staining was determined by brown dots. After rehydrating, the sections were immersed in periodic acid solution (0.5%, P7875, Sigma-Aldrich) for 5 min, rinsed in three changes of distilled water, incubated with Schiff’s reagent (3952016, Sigma-Aldrich) for 15 min and then rinsed in running tap water for 5 min. Nuclei were counterstained with haematoxylin 2 (220-102, Thermo Fisher Scientific) for 2 min. The sections were then rinsed in water, dehydrated in alcohol, cleared in xylene and finally mounted with Cytoseal XYL (8312-4, Thermo Fisher Scientific).

### Tissue cytometry and in situ cell classification

Tissue cytometry and analysis were conducted using the Volumetric Tissue Exploration and Analysis (VTEA) software (v.1.0a-r9). VTEA is a 3D image processing workspace that was developed as a plug-in for ImageJ^105^. The version of VTEA, which includes the supervised and unsupervised labelling of cells and combining spatial and features based gating strategies, used here is available at GitHub (https://github.com/icbm-iupui/volumetric-tissue-exploration-analysis) and through the FIJI updater. In this analytical pipeline, each individual nucleus was segmented using intensity thresholding and connected component segmentation built into VTEA and ImageJ. Each surveyed nucleus became a surrogate for a cell, to which the location and marker staining around or within the nucleus could be registered. This captured information could be used to classify cells on the basis of marker intensity or spatial features using scatterplot displays that enable various gating strategies and statistical analysis, including export as .csv files of all segmented cells and the associated features^106^. Cells classified on the basis of marker intensity are summarized in Supplementary Table 37. Gated cells were mapped back directly into the image volumes, which enabled immediate validation of the gates. Moreover, direct gating on the image could be performed, which could trace all of the cells within the chosen region-of-interest back to the data display on the scatter plot. Thus, cell classification could also be performed based on direct annotation of regions-of-interest (ROIs) within the image volumes. Annotated ROIs were determined by the pixel-wise agreement between 3 of 4 experts who performed annotation on each biopsy specimen separately.

Using tissue cytometry, 14 cell classes were defined based on the following features: (1) PT cells: AQP1^+^ cells in cortex ± brush border staining. (2) C-TAL cells: UMOD^+^ cells in cortex. (3) Glomerular cells (which encompass PODs, glomerular endothelium and mesangial cells) annotated ROIs based on morphology and F-actin staining. (4) Cortical large and medium vessel cells: annotated ROIs based on morphology and F-actin staining. (5) Cortical distal nephron cells (distal tubules (CD), connecting tubules (CNT) and collecting ducts (C-CD) or cortical distal nephrons): AQP1^−^UMOD^−^ and annotated ROIs based on unique morphology in cortex. (6) M-TAL cells: UMOD^+^ cells in the medulla. (7) DTL: AQP1^+^ cells in the medulla. (8) Medullary collecting ducts: AQP1^−^UMOD^−^ and annotated ROIs based on unique morphology in the medulla. (9) Vascular bundles in the medulla: annotated ROIs based onunique morphology in the medulla and F-actin staining. (10) Neutrophils: MPO^+^ cells. (11) Activated macrophages: MPO^−^CD68^+^ cells. (12) T cells: CD3^+^ cells. (13) Cells in altered regions: annotated ROIs based on loss of (unrecognizable) tubular morphology, expanded interstitium, increased fibrosis (by second harmonic generation imaging) and cell infiltrates. (14) Not determined: unable to be classified on the basis of the above criteria.

Using such an approach,1,540,563 cells were labelled from all the biopsies used in this analysis.

### 3D neighbourhood building and representation

3D neighbourhoods were calculated for every cell in each biopsy using VTEA and a radius of 25 μm (50 voxels in *x* and *y* and 25 voxels in *z*). We reasoned the largest measurable neighbourhood/niche in our 3D approach is limited by the 50 μm thickness of the sections imaged (*z* dimension). Thus, per Nyquist sampling, the radius used was about 25 μm, which is consistent with previous approaches^107–109^. For each 3D neighbourhood, VTEA was used to calculate the features: fraction-of-total and sum of each labelled cell per neighbourhood. A list of neighbourhoods, positions in 3D and their features was exported by biopsy sample as .csv files.

### Neighbourhood visualization and statistical analysis

CSV files of neighbourhoods by biopsy sample were generated in VTEA and imported into R (v.4.0.4), parsed for the sum of each labelled cell and monotypic neighbourhoods removed. These features were scaled by *Z*-standardization and used for Louvain community detection (R packages FNN (v.1.1.3) and igraph (v.1.2.6)) and *t*-SNE manifold projection (R package Rtsne (v.0.15)). To understand the interactions within neighbourhoods, pairwise interactions by neighbourhood were tallied and plotted on a chord plot (R package: circlize (v.0.4.12)) and Pearson’s correlation coefficients were calculated and plotted (R packages Hmisc (v.4.5.0) and corrplot (v.0.84)). Subclasses of neighbourhoods, those with at least one cell with a specific label were selected and plotted as network plots (R package igraph (v.1.2.6)) with edges in CD3 and Altered neighbourhoods scaled at 40% of all other subclasses to facilitate visualization. All scripts are provided as an annotated RStudio notebook file (.rmd).

### Plots and figures

UMAP, feature, dot and violin plots for snCv3, scCv3, SNARE2 and Visium data were generated using Seurat. Correlation plots were generated using the corrplot package. Genome coverage plots were performed using Signac. Plots for 3D cytometry and neighbourhood analysis were generated in R with circlize, ggplot2 and igraph.

### Reporting summary

Further information on research design is available in the Nature Portfolio Reporting Summary linked to this article.

## Online content

Any methods, additional references, Nature Portfolio reporting summaries, source data, extended data, supplementary information, acknowledgements, peer review information; details of author contributions and competing interests; and statements of data and code availability are available at 10.1038/s41586-023-05769-3.

### Supplementary information

Supplementary Information Legends for Supplementary Figs. 1–3, legends for Supplementary Tables 1–37; and a list of KPMP consortium members (not authors).

Reporting Summary

Supplementary Fig. 1

Supplementary Fig. 2

Supplementary Fig. 3

Supplementary Tables 1–37.

### Source data

Source Data Fig. 2

Source Data Fig. 3

Source Data Fig. 4

Source Data Fig. 5

Source Data Extended Data Fig. 1

Source Data Extended Data Fig. 2

Source Data Extended Data Fig. 3

Source Data Extended Data Fig. 4

Source Data Extended Data Fig. 5

Source Data Extended Data Fig. 6

Source Data Extended Data Fig. 8

Source Data Extended Data Fig. 11

Source Data Extended Data Fig. 12

## Data Availability

Processed data, interactive and visualization tools: the snCv3, scCv3, SNARE2, Slide-seq and Visium processed data files are all available for download at the GEO (Superseries GSE183279). snCv3 healthy reference data are available for reference-based single-cell mapping using the Azimuth tool (https://azimuth.hubmapconsortium.org/). All snCv3 and scCv3 processed data can be accessed and viewed at cellxgene (https://cellxgene.cziscience.com/collections/bcb61471-2a44-4d00-a0af-ff085512674c). snCv3 (excluding COVID-AKI and CKD nephrectomy samples), scCv3, Visium (KPMP biopsies) and 3D imaging can all be visualized and examined using the KPMP Data Atlas Explorer (https://atlas.kpmp.org/explorer/). For 3D imaging, the cytometry data, cell classifications, gates and neighbourhood analysis data are available at Zenodo (10.5281/zenodo.7120941). Raw sequencing and imaging data: raw sequencing data are under controlled access (human data) as they are potentially identifiable and can be accessed from the respective sources indicated below (summarized in Supplementary Tables 1 and 2). Raw and processed sequencing and imaging data (snCv3, scCv3, 3D imaging and Visium) generated as part of the KPMP have been deposited (https://atlas.kpmp.org/repository/) and compiled (10.48698/3z31-8924) online. 3D imaging raw data are freely available to download; however, KPMP raw sequencing data (snCv3, scCv3, Visium) have restricted access. These can be requested from KPMP by contacting A.L.D. (info@kpmp.org) and are available by signing a data use agreement (DUA) promising to abide by KPMP security standards and to not re-identify participants, share data outside those named on the DUA Exhibit A or sell the data. Data access is granted to anyone signing the KPMP DUA as is. KPMP will respond to initial data requests within 12–36 h and provide data up to one month after the DUA has been signed. Manuscripts resulting from KPMP controlled access data are requested to go through the KPMP publications and presentations (P&P) committee to ensure that KPMP is acknowledged appropriately and authorship follows ICJME standards. The KPMP P&P committee reviews and approves manuscripts every 2 weeks and, to date, no manuscript has been rejected. Any analysis resulting from KPMP data may be published or shared provided that it does not re-identify KPMP participants. Slide-seq raw sequencing data generated as part of KPMP pilot nephrectomy tissue are available for download from the GEO (Superseries GSE183279). Raw sequencing data (snCv3, SNARE2, Slide-seq) generated as part of the Human Biomolecular Atlas Project (HuBMAP) have been deposited (https://portal.hubmapconsortium.org/) and compiled (10.35079/hbm776.rgsw.867) online. The HuBMAP raw sequencing data have restricted access and are available for download from the database of Genotypes and Phenotypes (dbGaP: phs002249) by requesting for authorized access following instructions on the dbGaP website. The process to request access to this dbGaP study is available online (https://dbgap.ncbi.nlm.nih.gov/aa/wga.cgi?adddataset=phs002249&page=login). In brief, to download the human sequencing data for this study after obtaining authorization from the NIH DAC, one would go through the SRA (https://www.ncbi.nlm.nih.gov/bioproject/PRJNA671343). snCv3 data not deposited to KPMP or HuBMAP are available from the GEO (GSE183279) or, for Covid AKI raw sequencing files, on request from Washington University Kidney Translational Research Center (KTRC) to S.J. (sanjayjain@wustl.edu) due to patient confidentiality. Response to requests or questions will be addressed within a week. Code access and data use agreement forms can be accessed online (https://research.wustl.edu/core-facilities/ktrc/). Once the executed form is received and approved, data will be distributed within a month. There is no authorship restriction on the use of COVID data. Additional published/public datasets: the following publicly available RNA-seq datasets were used in this study: mouse kidney single cell (GEO: GSE129798); mouse kidney injury single nucleus (GEO: GSE139107); human fibroblast and myofibroblast single cell (Zenodo: 10.5281/zenodo.4059315); mouse distal nephron single cell and bulk distal segment (GEO: GSE150338); human kidney mature immune single cell (https://kidney-atlas.cells.ucsc.edu); and human kidney single nucleus (GEO: GSE151302; https://human-kidney-atac.cells.ucsc.edu). GWAS summary statistics were from the CKDGen Consortium (all eGFR; https://ckdgen.imbi.uni-freiburg.de/files/Wuttke2019), EBI GWAS Catalog (hypertension; https://www.ebi.ac.uk/gwas/efotraits/EFO_0000537) and the CausalDB database (release 1.1 2019-09-29; http://www.mulinlab.org/causaldb). NEPTUNE sequencing and clinical data were obtained from NEPTUNE. Owing to patient confidentiality, these data have restricted access and are available only on request to NEPTUNE-STUDY@umich.edu. ERCB data were obtained from the GEO (GSE104954). Raw sequencing data (scCv3) on living donor biopsies as part of the Chan Zuckerberg Initiative (CZI) and HCA are available from the GEO (GSE169285). Additional Visium spatial transcriptomic data not in the KPMP repository are available from the GEO (GSE171406). Figures: schemata of the human nephron and renal corpuscle were developed by the KPMP and HuBMAP (10.48698/DEM4-0Q93). Source data are provided with this paper.

## Code availability

Code to reproduce figures are available to download from GitHub (https://github.com/KPMP/Cell-State-Atlas-2022). No additional custom computational code was generated in this study.

## References

[1] Schreibing F, Kramann R (2022). Mapping the human kidney using single-cell genomics. Nat. Rev. Nephrol..

[2] Park J (2018). Single-cell transcriptomics of the mouse kidney reveals potential cellular targets of kidney disease. Science.

[3] Sheng L, Zhuang S (2020). New insights into the role and mechanism of partial epithelial-mesenchymal transition in kidney fibrosis. Front. Physiol..

[4] Kirita Y, Wu H, Uchimura K, Wilson PC, Humphreys BD (2020). Cell profiling of mouse acute kidney injury reveals conserved cellular responses to injury. Proc. Natl Acad. Sci. USA.

[5] Docherty M-H, O’Sullivan ED, Bonventre JV, Ferenbach DA (2019). Cellular senescence in the kidney. J. Am. Soc. Nephrol..

[6] Lameire NH (2013). Acute kidney injury: an increasing global concern. Lancet.

[7] Zuk A, Bonventre JV (2016). Acute kidney injury. Annu. Rev. Med..

[8] HuBMAP Consortium. (2019). The human body at cellular resolution: the NIH Human Biomolecular Atlas Program. Nature.

[9] de Boer IH (2021). Rationale and design of the Kidney Precision Medicine Project. Kidney Int..

[10] Regev, A. et al. The Human Cell Atlas. *eLife* **6**, e27041 (2017). 10.7554/eLife.27041 PMC5762154 29206104

[11] El-Achkar TM (2021). A multimodal and integrated approach to interrogate human kidney biopsies with rigor and reproducibility: guidelines from the Kidney Precision Medicine Project. Physiol. Genom..

[12] Lake BB (2019). A single-nucleus RNA-sequencing pipeline to decipher the molecular anatomy and pathophysiology of human kidneys. Nat. Commun..

[13] Menon R (2020). Single cell transcriptomics identifies focal segmental glomerulosclerosis remission endothelial biomarker. JCI Insight.

[14] Stewart BJ (2019). Spatiotemporal immune zonation of the human kidney. Science.

[15] Muto Y (2021). Single cell transcriptional and chromatin accessibility profiling redefine cellular heterogeneity in the adult human kidney. Nat. Commun..

[16] Chen S, Lake BB, Zhang K (2019). High-throughput sequencing of the transcriptome and chromatin accessibility in the same cell. Nat. Biotechnol..

[17] Bakken TE (2021). Comparative cellular analysis of motor cortex in human, marmoset and mouse. Nature.

[18] Plongthongkum N, Diep D, Chen S, Lake BB, Zhang K (2021). Scalable dual-omics profiling with single-nucleus chromatin accessibility and mRNA expression sequencing 2 (SNARE-seq2). Nat. Protoc..

[19] Rodriques SG (2019). Slide-seq: a scalable technology for measuring genome-wide expression at high spatial resolution. Science.

[20] Stickels RR (2021). Highly sensitive spatial transcriptomics at near-cellular resolution with Slide-seqV2. Nat. Biotechnol..

[21] Murray PT (2014). Potential use of biomarkers in acute kidney injury: report and summary of recommendations from the 10th Acute Dialysis Quality Initiative consensus conference. Kidney Int..

[22] Gerhardt LMS, Liu J, Koppitch K, Cippà PE, McMahon AP (2021). Single-nuclear transcriptomics reveals diversity of proximal tubule cell states in a dynamic response to acute kidney injury. Proc. Natl Acad. Sci. USA.

[23] Bussolati B (2005). Isolation of renal progenitor cells from adult human kidney. Am. J. Pathol..

[24] Cohen-Zontag O (2020). Human kidney clonal proliferation disclose lineage-restricted precursor characteristics. Sci Rep..

[25] Kuppe C (2021). Decoding myofibroblast origins in human kidney fibrosis. Nature.

[26] Andresen E, Günther G, Bullwinkel J, Lange C, Heine H (2011). Increased expression of beta-defensin 1 (DEFB1) in chronic obstructive pulmonary disease. PLoS ONE.

[27] Ferkowicz MJ (2021). Large-scale, three-dimensional tissue cytometry of the human kidney: a complete and accessible pipeline. Lab. Invest..

[28] Schueler M (2015). *DCDC2* mutations cause a renal-hepatic ciliopathy by disrupting Wnt signaling. Am. J. Hum. Genet..

[29] Yu J (2009). A Wnt7b-dependent pathway regulates the orientation of epithelial cell division and establishes the cortico-medullary axis of the mammalian kidney. Development.

[30] Park J-S, Valerius MT, McMahon AP (2007). Wnt/beta-catenin signaling regulates nephron induction during mouse kidney development. Development.

[31] Miller RK, McCrea PD (2010). Wnt to build a tube: contributions of Wnt signaling to epithelial tubulogenesis. Dev. Dyn..

[32] Patel S (2019). Rac-GTPase promotes fibrotic TGF-β1 signaling and chronic kidney disease via EGFR, p53, and Hippo/YAP/TAZ pathways. FASEB J..

[33] Edeling M, Ragi G, Huang S, Pavenstädt H, Susztak K (2016). Developmental signalling pathways in renal fibrosis: the roles of Notch, Wnt and Hedgehog. Nat. Rev. Nephrol..

[34] Meecham A, Marshall JF (2020). The ITGB6 gene: its role in experimental and clinical biology. Gene X.

[35] Nanjundan M (2003). Plasma membrane phospholipid scramblase 1 promotes EGF-dependent activation of c-Src through the epidermal growth factor receptor. J. Biol. Chem..

[36] Harskamp LR, Gansevoort RT, van Goor H, Meijer E (2016). The epidermal growth factor receptor pathway in chronic kidney diseases. Nat. Rev. Nephrol..

[37] Puri P (2018). Ectopic phosphorylated Creb marks dedifferentiated proximal tubules in cystic kidney disease. Am. J. Pathol..

[38] Sengez B (2019). The transcription factor Elf3 is essential for a successful mesenchymal to epithelial transition. Cells.

[39] Marneros AG (2020). AP-2β/KCTD1 control distal nephron differentiation and protect against renal fibrosis. Dev. Cell.

[40] Ju W (2015). Tissue transcriptome-driven identification of epidermal growth factor as a chronic kidney disease biomarker. Sci. Transl. Med..

[41] Dendooven A (2011). Loss of endogenous bone morphogenetic protein-6 aggravates renal fibrosis. Am. J. Pathol..

[42] Chetty A, Cao G-J, Nielsen HC (2006). Insulin-like growth factor-I signaling mechanisms, type I collagen and alpha smooth muscle actin in human fetal lung fibroblasts. Pediatr. Res..

[43] Wu Z, Yu Y, Niu L, Fei A, Pan S (2016). IGF-1 protects tubular epithelial cells during injury via activation of ERK/MAPK signaling pathway. Sci. Rep..

[44] Gadegbeku CA (2013). Design of the Nephrotic Syndrome Study Network (NEPTUNE) to evaluate primary glomerular nephropathy by a multidisciplinary approach. Kidney Int..

[45] Yasuda, Y., Cohen, C. D., Henger, A., Kretzler, M. & European Renal cDNA Bank (ERCB) Consortium. Gene expression profiling analysis in nephrology: towards molecular definition of renal disease. *Clin. Exp. Nephrol.* **10**, 91–98 (2006). 10.1007/s10157-006-0421-z 16791393

[46] Krid H, Dorison A, Salhi A, Cheval L, Crambert G (2012). Expression profile of nuclear receptors along male mouse nephron segments reveals a link between ERRβ and thick ascending limb function. PLoS ONE.

[47] Wang, X. X. et al. Estrogen-related receptor agonism reverses mitochondrial dysfunction and inflammation in the aging kidney. Preprint at *bioRxiv*10.1101/755801 (2020).10.1016/j.ajpath.2023.07.00837717940

[48] Tabula Muris Consortium. (2020). A single-cell transcriptomic atlas characterizes ageing tissues in the mouse. Nature.

[49] Hansen J (2022). A reference tissue atlas for the human kidney. Sci. Adv..

[50] Eadon MT (2020). Kidney histopathology and prediction of kidney failure: a retrospective cohort study. Am. J. Kidney Dis..

[51] Martin M (2011). Cutadapt removes adapter sequences from high-throughput sequencing reads. EMBnet J..

[52] Petukhov V (2018). dropEst: pipeline for accurate estimation of molecular counts in droplet-based single-cell RNA-seq experiments. Genome Biol..

[53] Dobin A (2013). STAR: ultrafast universal RNA-seq aligner. Bioinformatics.

[54] Fang R (2021). Comprehensive analysis of single cell ATAC-seq data with SnapATAC. Nat. Commun..

[55] Li H (2018). Minimap2: pairwise alignment for nucleotide sequences. Bioinformatics.

[56] Gayoso, A., Shor, J., Carr, A. J., Sharma, R. & Pe’er, D. GitHub: DoubletDetection (2019); 10.5281/zenodo.2678042.

[57] Young MD, Behjati S (2020). SoupX removes ambient RNA contamination from droplet-based single-cell RNA sequencing data. Gigascience.

[58] Chen L, Chou C-L, Knepper MA (2021). A comprehensive map of mRNAs and their isoforms across all 14 renal tubule segments of mouse. J. Am. Soc. Nephrol..

[59] Ransick A (2019). Single-cell profiling reveals sex, lineage, and regional diversity in the mouse kidney. Dev. Cell.

[60] Börner, K. et al. Anatomical structures, cell types and biomarkers of the Human Reference Atlas. *Nat. Cell Biol.* **23**, 1117–1128 (2021). 10.1038/s41556-021-00788-6 PMC10079270 34750582

[61] Chen L, Chou C-L, Knepper MA (2021). Targeted single-cell RNA-seq identifies minority cell types of kidney distal nephron. J. Am. Soc. Nephrol..

[62] Monaco G (2019). RNA-seq signatures normalized by mRNA abundance allow absolute deconvolution of human immune cell types. Cell Rep..

[63] Hao Y (2021). Integrated analysis of multimodal single-cell data. Cell.

[64] Barkas N (2019). Joint analysis of heterogeneous single-cell RNA-seq dataset collections. Nat. Methods.

[65] Luecken MD (2022). Benchmarking atlas-level data integration in single-cell genomics. Nat. Methods.

[66] Aevermann BD (2018). Cell type discovery using single-cell transcriptomics: implications for ontological representation. Hum. Mol. Genet..

[67] Aran D (2019). Reference-based analysis of lung single-cell sequencing reveals a transitional profibrotic macrophage. Nat. Immunol..

[68] Heng, T. S. P., Painter, M. W. & Immunological Genome Project Consortium. The Immunological Genome Project: networks of gene expression in immune cells. *Nat. Immunol.* **9**, 1091–1094 (2008). 10.1038/ni1008-1091 18800157

[69] Takemon Y (2021). Proteomic and transcriptomic profiling reveal different aspects of aging in the kidney. eLife.

[70] Ruscetti M (2018). NK cell-mediated cytotoxicity contributes to tumor control by a cytostatic drug combination. Science.

[71] Basisty N (2020). A proteomic atlas of senescence-associated secretomes for aging biomarker development. PLoS Biol..

[72] Korotkevich, G. et al. Fast gene set enrichment analysis. Preprint at *bioRxiv*10.1101/060012 (2016).

[73] Luo W, Friedman MS, Shedden K, Hankenson KD, Woolf PJ (2009). GAGE: generally applicable gene set enrichment for pathway analysis. BMC Bioinform..

[74] Stuart, T. et al. Single-cell chromatin state analysis with Signac. *Nat. Methods* **18**, 1333–1341 (2021). 10.1038/s41592-021-01282-5 PMC9255697 34725479

[75] Schep AN, Wu B, Buenrostro JD, Greenleaf WJ (2017). chromVAR: inferring transcription-factor-associated accessibility from single-cell epigenomic data. Nat. Methods.

[76] Bravo González-Blas C (2019). cisTopic: *cis*-regulatory topic modeling on single-cell ATAC-seq data. Nat. Methods.

[77] Pliner HA (2018). Cicero predicts *cis*-regulatory DNA interactions from single-cell chromatin accessibility data. Mol. Cell.

[78] Yu G, Wang L-G, He Q-Y (2015). ChIPseeker: an R/Bioconductor package for ChIP peak annotation, comparison and visualization. Bioinformatics.

[79] Wu Y, Tamayo P, Zhang K (2018). Visualizing and interpreting single-cell gene expression datasets with similarity weighted nonnegative embedding. Cell Syst..

[80] Jin S (2021). Inference and analysis of cell-cell communication using CellChat. Nat. Commun..

[81] Ulirsch JC (2019). Interrogation of human hematopoiesis at single-cell and single-variant resolution. Nat. Genet..

[82] Wang J (2020). CAUSALdb: a database for disease/trait causal variants identified using summary statistics of genome-wide association studies. Nucleic Acids Res..

[83] Chen W (2015). Fine mapping causal variants with an approximate bayesian method using marginal test statistics. Genetics.

[84] Canela-Xandri O, Rawlik K, Tenesa A (2018). An atlas of genetic associations in UK Biobank. Nat. Genet..

[85] Tin A (2019). Target genes, variants, tissues and transcriptional pathways influencing human serum urate levels. Nat. Genet..

[86] Wuttke M (2019). A catalog of genetic loci associated with kidney function from analyses of a million individuals. Nat. Genet..

[87] Zhu Z (2019). Genetic overlap of chronic obstructive pulmonary disease and cardiovascular disease-related traits: a large-scale genome-wide cross-trait analysis. Respir. Res..

[88] Li Y (2020). Integration of GWAS summary statistics and gene expression reveals target cell types underlying kidney function traits. J. Am. Soc. Nephrol..

[89] Sheng X (2021). Mapping the genetic architecture of human traits to cell types in the kidney identifies mechanisms of disease and potential treatments. Nat. Genet..

[90] Cippà PE (2018). Transcriptional trajectories of human kidney injury progression. JCI Insight.

[91] Barisoni L (2013). Digital pathology evaluation in the multicenter Nephrotic Syndrome Study Network (NEPTUNE). Clin. J. Am. Soc. Nephrol..

[92] Levey AS (2014). GFR decline as an end point for clinical trials in CKD: a scientific workshop sponsored by the National Kidney Foundation and the US Food and Drug Administration. Am. J. Kidney Dis..

[93] Tao J (2018). JAK-STAT signaling is activated in the kidney and peripheral blood cells of patients with focal segmental glomerulosclerosis. Kidney Int..

[94] Street K (2018). Slingshot: cell lineage and pseudotime inference for single-cell transcriptomics. BMC Genom..

[95] Zhang, B. & Horvath, S. A general framework for weighted gene co-expression network analysis. *Stat. Appl. Genet. Mol. Biol.* **4**, Article17 (2005). 10.2202/1544-6115.1128 16646834

[96] Jassal B (2020). The reactome pathway knowledgebase. Nucleic Acids Res..

[97] La Manno G (2018). RNA velocity of single cells. Nature.

[98] Bergen V, Lange M, Peidli S, Wolf FA, Theis FJ (2020). Generalizing RNA velocity to transient cell states through dynamical modeling. Nat. Biotechnol..

[99] Stickels, R. et al. Library generation using slide-seqV2 v2. *Protocols.io*10.17504/protocols.io.bvv6n69e (2021).

[100] Dries R (2021). Giotto: a toolbox for integrative analysis and visualization of spatial expression data. Genome Biol..

[101] Cable DM (2021). Robust decomposition of cell type mixtures in spatial transcriptomics. Nat. Biotechnol..

[102] Melo Ferreira R (2021). Integration of spatial and single cell transcriptomics localizes epithelial-immune cross-talk in kidney injury. JCI Insight.

[103] Janosevic D (2021). The orchestrated cellular and molecular responses of the kidney to endotoxin define a precise sepsis timeline. eLife.

[104] Melo Ferreira R, Freije BJ, Eadon MT (2021). Deconvolution tactics and normalization in renal spatial transcriptomics. Front. Physiol..

[105] Winfree S (2017). Quantitative three-dimensional tissue cytometry to study kidney tissue and resident immune cells. J. Am. Soc. Nephrol..

[106] Winfree S (2017). Large-scale 3-dimensional quantitative imaging of tissues: state-of-the-art and translational implications. Transl. Res..

[107] Stoltzfus CR (2020). CytoMAP: a spatial analysis toolbox reveals features of myeloid cell organization in lymphoid tissues. Cell Rep..

[108] Stoltzfus CR (2021). Multi-parameter quantitative imaging of tumor microenvironments reveals perivascular immune niches associated with anti-tumor immunity. Front. Immunol..

[109] Leal JM (2021). Innate cell microenvironments in lymph nodes shape the generation of T cell responses during type I inflammation. Sci. Immunol..

