# Supplementary material for: An atlas of healthy and injured cell states and niches in the human kidney
Source: Nature. 2023 Jul 19;619(7970):585–94. doi: 10.1038/s41586-023-05769-3 (PMC10356613; doi:10.1038/s41586-023-05769-3)
Supplement: Supplementary file 1 — Legends for Supplementary Figs. 1–3, legends for Supplementary Tables 1–37; and a list of KPMP consortium members (not authors). [file 41586_2023_5769_MOESM1_ESM.pdf]

nature portfolio

https://doi.org/10.1038/s41586-023-05769-3

Supplementary information

An atlas of healthy and injured cell states and niches in the human kidney

In the format provided by the
authors and unedited

Supplemental Data

An atlas of healthy and injured cell states and niches in the human kidney

Blue B. Lake[1,21,41], Rajasree Menon[2,41], Seth Winfree[3,41], Qiwen Hu[4,41], Ricardo Melo Ferreira[5,41], Kian Kalhor[1,41], Daria Barwinska[5], Edgar A. Otto[6], Michael Ferkowicz[5], Dinh Diep[1,21], Nongluk Plongthongkum[1], Amanda Knoten[7], Sarah Urata[1], Laura Mariani[6], Abhijit S. Naik[6], Sean Eddy[6], Bo Zhang[7], Yan Wu[1,21], Diane Salamon[7], James C. Williams[5], Xin Wang[4], Karol S. Balderrama[8], Paul Hoover[8], Evan Murray[8], Jamie L. Marshall[8], Teia Noel[8], Anitha Vijayan[7], Austin Hartman[9], Fei Chen[8], Sushrut S. Waikar[10], Sylvia Rosas[11], Francis P. Wilson[12], Paul M. Palevsky[13], Krzysztof Kiryluk[14], John R. Sedor[15], Robert D. Toto[16], Chirag R. Parikh[17], Eric H. Kim[18], Rahul Satija[9], Anna Greka[8], Evan Z. Macosko[8], Peter V. Kharchenko[4,21], Joseph P. Gaut[19], Jeffrey B. Hodgin[20], KPMP consortium*, Michael T. Eadon[5, ǂ], Pierre C. Dagher[5, ǂ], Tarek M. El-Achkar[5, ǂ], Kun Zhang[1,21, ǂ], Matthias Kretzler[6, ǂ], Sanjay Jain[7,19, ǂ].

[1]Department of Bioengineering, University of California, San Diego, La Jolla, CA 92093, USA
[2]Department of Computational Medicine and Bioinformatics, University of Michigan, Ann Arbor, MI 48109, USA
[3]Department of Pathology and Microbiology, University of Nebraska Medical Center, Omaha, NE 68198, USA
[4]Department of Biomedical Informatics, Harvard Medical School, Boston, MA 02115, USA
[5]Department of Medicine, Indiana University School of Medicine, Indianapolis, IN 46202, USA
[6]Department of Internal Medicine, Division of Nephrology, University of Michigan, Ann Arbor, MI 48109, USA
[7]Department of Medicine, Washington University School of Medicine, St. Louis, MO 63110, USA
[8]Broad Institute of Harvard and MIT, Cambridge, MA 02142, USA
[9]New York Genome Center, New York, NY 10013, USA
[10]Section of Nephrology, Boston University School of Medicine and Boston Medical Center, Boston, MA 02118, USA
[11]Joslin Diabetes Center and Harvard Medical School, Boston, MA 02215, USA
[12]Department of Medicine, Yale University School of Medicine, New Haven, CT 06510, USA
[13]Department of Medicine, University of Pittsburgh School of Medicine, Pittsburgh, PA 15213, USA
[14]Department of Medicine, Columbia University, New York, NY 10032, USA
[15]Lerner Research and Glickman Urology and Kidney Institutes, Cleveland Clinic, Cleveland, OH 44195, USA
[16]Department of Internal Medicine, UT Southwestern Medical Center, Dallas, TX 75390, USA
[17]Division of Nephrology, Johns Hopkins School of Medicine, Baltimore, MD 21287, USA
[18]Department of Surgery, Washington University School of Medicine, St. Louis, MO 63110, USA
[19]Department of Pathology and Immunology, Washington University School of Medicine, St. Louis, MO 63110, USA
[20]Department of Pathology, University of Michigan, Ann Arbor, MI 48109, USA
[21]Present address: San Diego Institute of Science, Altos Labs, San Diego, CA 92121, USA
*A list of authors and their affiliations appears at the end of the paper.
[41]These authors contributed equally: Blue B. Lake, Rajasree Menon, Seth Winfree, Qiwen Hu, Ricardo Melo Ferreira, Kian Kalhor

ǂCorresponding Authors:
Sanjay Jain sanjayjain@wustl.edu
Matthias Kretzler kretzler@med.umich.edu
Kun Zhang kzhang@bioeng.ucsd.edu
Tarek M. El-Achkar telachka@iu.edu
Pierre C. Dagher pdaghe2@iu.edu
Michael T. Eadon meadon@iupui.edu

This data file (excel) file includes:

1. Supplementary Table Legends
2. Supplementary Figure Legends
3. KPMP Consortium member list (not authors)

Supplementary Table Legends

Supplementary Table 1. Omic experiments. Summary of omic experiments performed on 35 reference tissue donors and 36 disease tissue patients, including information for accessing raw data.

Supplementary Table 2. 3D imaging and spatial transcriptomic experiments. Summary of spatial experiments encompassing 11 reference tissue donors and 30 disease tissue patients, including information for accessing raw data.

Supplementary Table 3. Sample Clinical Data. Clinical and demographic attributes for all 45 healthy and 48 disease kidney tissue donors used in this study. Abbreviations: BMI, body mass index; BUN, blood urea nitrogen; Cr, creatinine; eGFR, estimated glomerular filtration rate; A1C, hemoglobin A1C.

Supplementary Table 4. snCv3 cluster annotations. Cell type annotations at different subclass levels and corresponding cell state identities for all snCv3 clusters. Mean genes, mean UMI and number of nuclei per region, condition and patient for all clusters are provided.

Supplementary Table 5. snCv3 Cell Type Marker Genes. Curated table of marker genes identified from both this study and published sources (indicated references) for all snCv3 subclasses.

Supplementary Table 6. scCv3 cluster annotations. Cell type annotations at different subclass levels and corresponding cell state identities for all scCv3 predicted subclasses. Mean genes, mean UMI and number of cells per condition and patient for all clusters are provided.

Supplementary Table 7. SNARE2 (RNA/AC) cluster annotations. Cell type annotations at different subclass levels and corresponding cell state identities for all SNARE2 predicted clusters (Post-AC processing and clusters > 50 nuclei, Methods). Mean genes, mean UMI and number of nuclei per region, condition and patient for all clusters are provided.

Supplementary Table 8. snCv3 and scCv3 Computational Marker Genes. Marker genes identified using NSForestv2 for all snCv3 clusters and snCv3/scCv3 subclasses. The minimal set of combinatorial markers distinguishing each cell population are listed. Binary markers identified for each subclass, either unique to that subclass or also identified within other subclasses, are listed.

Supplementary Table 9. Cell type-specific epigenomic features. Left table: SNARE2 accessible chromatin data was used to identify differentially accessible regions (DARs) across all subclasses. P-values were calculated using Fisher's exact test on a hypergeometric distribution and adjusted P values (or Q values) were calculated using the Benjamini–Hochberg method. Middle table: transcription factor (TF) motif enrichments were identified for marker gene associated DARs. Observed - number of features containing the motif; background - total number of features containing the motif; P-values were calculated using a hypergeometric test. Right table: subclass-specific TF motif accessibilities assessed using ChromVar. P-Values were calculated using the Wilcoxon Rank Sum test and adjusted P-Values were calculated using the Benjamini–Hochberg method.

Supplementary Table 10. Cell state definitions. Table of cell states and their corresponding descriptions. Subclass groupings used to identify state-specific gene expression signatures are provided.

Supplementary Table 11. aEpi differentially expressed genes (snCv3). Left table: differentially expressed genes (DEGs) between proximal tubules (PT) and adaptive PT (aPT) for snCv3 that were found conserved across patient conditions (Reference, AKI, CKD). P values were calculated using the Wilcoxon Rank Sum test and adjusted P values were calculated using the Benjamini–Hochberg method. Maximum and minimum P values across the conditions are provided. Right table: same as for the left table except showing conserved DEGs between the thick ascending limb (TAL) and adaptive TAL (aTAL).

Supplementary Table 12. Conserved degenerative state marker genes. List of degenerative marker genes identified separately for snCv3 and scCv3. Filter criteria used to achieve minimal and expanded gene sets are provided. Indicated P values (pval) were calculated using the Wilcoxon Rank Sum test and adjusted P values (pval_adj) were calculated using the Benjamini–Hochberg method. Log2fc is log2 fold change; number of cell types refers to the number of cell types having degenerative states where the gene was identified as a degen marker.

Supplementary Table 13. Conserved adaptive epithelial (aEpi) state marker genes. List of adaptive epithelial (aPT/aTAL) marker genes identified separately for snCv3 and scCv3. Filter criteria used to achieve minimal, full and expanded gene sets are provided. Indicated P values (pval) were calculated using the Wilcoxon Rank Sum test and adjusted P values (pval_adj) were calculated using the Benjamini–Hochberg method. Log2fc is log2 fold change.

Supplementary Table 14. Conserved adaptive stromal (aStr) state marker genes. List of adaptive stromal (aFIB, MYOF) marker genes identified separately for snCv3 and scCv3. Filter criteria used to achieve minimal, full and expanded gene sets are provided. Indicated P values (pval) were calculated using the Wilcoxon Rank Sum test and adjusted P values (pval_adj) were calculated using the Benjamini–Hochberg method. Log2fc is log2 fold change; number of cell types refers to the number of adaptive states (aFIB, MYOF) where the gene was identified as an adaptive marker.

Supplementary Table 15. Conserved cycling state marker genes. List of cycling genes identified separately for snCv3 and scCv3. Filter criteria used to achieve minimal and full gene sets are provided. Indicated P values (pval) were calculated using the Wilcoxon Rank Sum test and adjusted P values (pval_adj) were calculated using the Benjamini–Hochberg method. Log2fc is log2 fold change.

Supplementary Table 16. Adaptive PT trajectory gene expression modules. Genes showing expression differences as a function of estimated pseudo-time across the proximal tubule (PT) trajectory (snCv3). Corresponding trajectory modules are indicated. P values were calculated using F-test and adjusted P values were calculated using the Benjamini–Hochberg method.

Supplementary Table 17. Adaptive TAL trajectory gene expression modules. Genes showing expression differences as a function of estimated pseudo-time across the thick ascending limb (TAL) trajectory (snCv3). Corresponding trajectory modules are indicated. P values were calculated using F-test and adjusted P values were calculated using the Benjamini–Hochberg method.

Supplementary Table 18. Adaptive epithelial module reactome pathway analyses. Pathway analysis using the Reactome online tool (reactome.org/PathwayBrowser/) for gene sets enriched in each aPT or aTAL module. P values were calculated using a Binomial Test and adjusted P values, or False Discovery Rates (FDR), were calculated using the Benjamini-Hochberg approach.

Supplementary Table 19. Predicted PT and TAL state TF activities. TF activity scores predicted from PT and TAL subclass DEGs. Average values indicate the predicted mean TF activity for a specific cell population.

Supplementary Table 20. aEpi trajectory TFBS accessibilities. Differential TFBS accessibilities (ChromVar) were identified from SNARE2 data for each of the aEpi (aPT and aTAL) trajectory modules. P values were calculated using the Wilcoxon Rank Sum test and adjusted P values were calculated using the Benjamini–Hochberg method.

Supplementary Table 21. aTAL dynamical genes. Left table: the top dynamical genes by latent time identified using scVelo for all TAL modules. Right tables: associated gene ontologies for each TAL module gene set found in the left table. P values were calculated using the Fisher's Exact test and adjusted P values (FDR) were calculated using the Benjamini–Hochberg method.

Supplementary Table 22. TAL Module Gene Regulatory Networks (GRNs). Network scores and corresponding metrics for TAL trajectory module-specific GRNs that were generated using Celloracle.

Supplementary Table 23. TAL Module GRN Filtered Links. TF and corresponding gene target links that were identified from TAL trajectory module-specific GRNs (Celloracle). P values were calculated using one-sample t-tests.

Supplementary Table 24. TAL Module GRN TF Targets. Left table: summary of module-specific target genes for select TFs (individual or grouped by signaling pathways) from TAL trajectory module-specific GRNs (Supplementary Table 23). P values were calculated using one-sample t-tests. Right tables: corresponding gene ontology analyses for gene sets shown in left tables. P values were calculated using the Fisher's Exact test and adjusted P values (FDR) were calculated using the Benjamini–Hochberg method.

Supplementary Table 25. TAL repair niche ligand-receptor pairs. CellChat ligand-receptor pairs between TAL repair (adaptive/maladaptive) niche cell types.

Supplementary Table 26. Stromal (STR) Gene Regulatory Networks (GRNs). Network scores and corresponding metrics for STR subpopulation-specific GRNs that were generated using Celloracle.

Supplementary Table 27. STR GRN Filtered Links. TF and corresponding gene target links that were identified from STR subpopulation-specific GRNs (Celloracle). P values were calculated using one-sample t-tests.

Supplementary Table 28. STR GRN TF Targets. Left table: summary of subclass-specific target genes for select TFs (individual or grouped by signaling pathways) from STR subpopulation-specific GRNs (Supplementary Table 27). P values were calculated using one-sample t-tests. Right tables: corresponding gene ontology analyses for gene sets shown in left tables. P values were calculated using the Fisher's Exact test and adjusted P values (FDR) were calculated using the Benjamini–Hochberg method.

Supplementary Table 29. Clinical outcomes gene sets. Altered state gene sets used for clinical outcomes assessment.

Supplementary Table 30. Clinical analyses of altered state scores. Results of clinical outcomes assessments for altered state gene sets using the NEPTUNE (left tables) and ERCB (right tables) cohorts.

Supplementary Table 31. Motif enrichments within TAL-associated causal SNP peaks. Transcription factor (TF) motif enrichments were identified for chromosomal regions colocalizing with kidney disease- or trait-associated causal variant SNPs. Only chromosomal regions that were further linked to genes expressed within the TAL were used. Observed - number of features containing the motif; background - total number of features containing the motif; P-values were calculated using a hypergeometric test.

Supplementary Table 32. Gene sets that distinguish AKI and CKD patients in aPT and aTAL trajectories. Left tables: gene sets that best differentiate AKI from CKD patients in PT and TAL cell populations (snCv3 and scCv3) as measured by their area under the curve (AUC) scores. Right tables: corresponding functional gene module detection (HumanBase) was performed for gene sets shown in left tables. Q values are calculated using one-sided Fisher's exact tests and the Benjamini–Hochberg correction method.

Supplementary Table 33. Gene sets that distinguish AKI and CKD patients in broad subclasses. Genes that were differentially expressed (DEGs) between AKI and CKD across all subclasses (level 1). P values were calculated using the DESeq2 Wald test adjusted P values were calculated using the Benjamini–Hochberg method.

Supplementary Table 34. Neptune cohort biopsy characteristics. Time of biopsy characteristics of participants in Neptune cohort used in this study. Continuous, normally distributed variables are presented as mean (SD). Continuous, non-normally distributed variables are presented as median (IQR).

Supplementary Table 35. 3D imaging antibody table. Antibodies and dilutions used in mesoscale 3D imaging for 3D cytometry.

Supplementary Table 36. Immunofluorescent (IF) imaging antibody table. Antibodies and dilutions used in confocal IF imaging.

Supplementary Table 37. 3D cytometry markers. Tabulation of select marker based 3D cytometry results.

Supplementary Figure Legends

Supplementary Figure 1. SNARE2 cell type regulatory profiles. a. Coverage plots showing SNARE2 AC read pile-ups for genomic regions associated with cell type marker genes. Violin plots show corresponding SNARE2 RNA gene expression values. **b.** Heatmaps showing averaged scaled chromatin accessibility values for differentially accessible regions (DARs) identified for cell type specific differentially expressed genes (DEGs, Methods). Select TF motifs enriched within the cell type specific DARs are shown. **c.** Dot plots showing average TFBS accessibilities (chromVAR) and proportion accessible for SNARE2 AC cell types. This permitted discovery of cell-type-specific TF activities, including HNF4A in proximal tubule (PT), ESRRB in the TAL, GATA3 in the collecting tubules, FOXI1 in IC cells, SOX17 in ECs and MEF2D in VSMC/P.

Supplementary Figure 2. Altered state expression signatures. a. Violin plots showing degenerative state scores and degenerative features (percent mitochondrial transcripts; percent ER or ribosomal transcripts; *CST3*, *CLU* and *IGFBP7* expression) for reference or degenerative states of snCv3 level 1 subclasses. **b.** Dot plots showing SNARE2 average accessibilities (chromVAR) and proportion accessible for common degenerative TFBSs showing differential activity in 3 or more subclass level 1 cell types.

Supplementary Figure 3. A healthy kidney reference atlas. a. UMAP plot of reference state level 3 subclasses for both snCv3 and SNARE2 (RNA) data. Only samples with condition level 1 and state level 1 being classified as reference were included (see **Supplementary Tables 1**, **4** and **7**). Insets show mapping of the tissue region, sex and assay identities. **b.** Dot plot showing averaged marker gene expression values (log scale) and proportion expressed for integrated snCv3/SNARE2 **(a)** level 3 subclasses.

KPMP Consortium Members other than authors

Publication Name	Site
Steve Bogen	Boston Cell Standards
Laurence H. Beck	Boston University
Joel M. Henderson	Boston University
Ingrid Onul	Boston University
Ashish Verma	Boston University
M. Todd Valerius	Brigham & Women's Hospital
Mia R. Colona	Brigham & Women's Hospital
Yijiang M. Chen	Case Western Reserve
Andrew Janowczyk	Case Western Reserve
Vidya S. Viswanathan	Case Western Reserve
William S. Bush	Case Western Reserve University
Dana C. Crawford	Case Western Reserve University
Anant Madabhushi	Case Western Reserve University
Petter M. Bjornstad	Colorado
Laura Pyle	Colorado
Helmut Rennke	Harvard University
Sharon Bledsoe	Indiana University
Andreas Bueckle	Indiana University
Bruce W. Herr	Indiana University
Ellen M. Quardokus	Indiana University
Elizabeth Record	Indiana University
Marcelino Rivera	Indiana University
Jing Su	Indiana University
Pottumarthi Prasad	Northshore
Anand Srivastava	Northwestern
John P. Shapiro	Ohio State University
Jessica Lukowski	Pacific Northwest National Laboratories
George (Holt) Oliver	Parkland
Aaron Wong	Princeton University
Ari Pollack	Seattle Children's Hospital
Yury Goltsev	Stanford University
Nicholas Lucarelli	SUNY Buffalo
Pinaki Sarder	SUNY Buffalo
Tariq Mukatash	UC San Francisco
Rita R. Alloway	University of Cincinnati
Ashley R. Burg	University of Cincinnati
Paul J. Lee	University of Cincinnati
Adele Rike	University of Cincinnati

Tiffany Shi	University of Cincinnati
E. Steve Woodle	University of Cincinnati
Ninive C. Conser	University of Michigan
Jinghui Luo	University of Michigan
Renee Frey	University of Michigan
Ashley Berglund	University of Washington
Brooke Berry	University of Washington
Christine Limonte	University of Washington
Natalya Sarkisova	University of Washington
Kasra Rezaei	University of Washington
Jamie Snyder	University of Washington
Ruikang Wang	University of Washington
Stephanie M. Grewenow	University of Washington
Richard Montellano	UT Health San Antonio
Hongping Ye	UT Health San Antonio
S. Susan Hedayati	UT Southwestern
Asra Kermani	UT Southwestern
Christopher Y. Lu	UT Southwestern
R. Tyler Miller	UT Southwestern
Orson W. Moe	UT Southwestern
Jiten Patel	UT Southwestern
Anil Pillai	UT Southwestern
Kamalanathan Sambandam	UT Southwestern
Jose Torrealba	UT Southwestern
Miguel Vazquez	UT Southwestern
Nancy Wang	UT Southwestern
Dianbo Zhang	UT Southwestern
Simon C. Lee	UT Southwestern
Harold Park	UT Southwestern
Natasha Wen	UT Southwestern
Richard M. Caprioli	Vanderbilt
Nathan Patterson	Vanderbilt
Kavya Sharman	Vanderbilt
Jeffrey M. Spraggins	Vanderbilt
Raf Van de Plas	Vanderbilt
Jeanine Basta	Washington University St. Louis
Sabine M. Diettman	Washington University St. Louis
Michael I. Rauchman	Washington University St. Louis
Lloyd G. Cantley	Yale University
