# Supplementary material for: An atlas of healthy and injured cell states and niches in the human kidney
Source: Nature. 2023 Jul 19;619(7970):585–94. doi: 10.1038/s41586-023-05769-3 (PMC10356613; doi:10.1038/s41586-023-05769-3)
Supplement: Supplementary file 2 — Reporting Summary [file 41586_2023_5769_MOESM2_ESM.pdf]

nature portfolio

Corresponding author(s): Michael T. Eadon, Pierre C. Dagher, TarekM. el-Achkar, Kun Zhang, Matthias Kretzler, Sanjay Jain

Last updated by author(s): Oct 20, 2022

Reporting Summary

Nature Portfolio wishes to improve the reproducibility of the work that we publish. This form provides structure for consistency and transparency in reporting. For further information on Nature Portfolio policies, see our Editorial Policies and the Editorial Policy Checklist.

Statistics

For all statistical analyses, confirm that the following items are present in the figure legend, table legend, main text, or Methods section.

n/a	Confirmed	
☐	☒	The exact sample size (*n*) for each experimental group/condition, given as a discrete number and unit of measurement
☐	☒	A statement on whether measurements were taken from distinct samples or whether the same sample was measured repeatedly
☐	☒	The statistical test(s) used AND whether they are one- or two-sided<br>*Only common tests should be described solely by name; describe more complex techniques in the Methods section.*
☐	☒	A description of all covariates tested
☐	☒	A description of any assumptions or corrections, such as tests of normality and adjustment for multiple comparisons
☐	☒	A full description of the statistical parameters including central tendency (e.g. means) or other basic estimates (e.g. regression coefficient) AND variation (e.g. standard deviation) or associated estimates of uncertainty (e.g. confidence intervals)
☐	☒	For null hypothesis testing, the test statistic (e.g. *F*, *t*, *r*) with confidence intervals, effect sizes, degrees of freedom and *P* value noted<br>*Give P values as exact values whenever suitable.*
☒	☐	For Bayesian analysis, information on the choice of priors and Markov chain Monte Carlo settings
☒	☐	For hierarchical and complex designs, identification of the appropriate level for tests and full reporting of outcomes
☐	☒	Estimates of effect sizes (e.g. Cohen's *d*, Pearson's *r*), indicating how they were calculated

Our web collection on statistics for biologists contains articles on many of the points above.

Software and code

Policy information about availability of computer code

Data collection

10x Chromium v3 and Illumina Novaseq 6000 instrument control software (v1.6.0 and 1.7.0); Leica LASX software (v. 3.5); 3D label free autofluorescence and fluorescence imaging data were captured using a Leica SP8 confocal scan-head mounted to an upright DM6000 microscope. For large-scale imaging of tissues at submicron resolution, the Leica Tile Scan function was used to collect a mosaic of smaller image volumes using a high-power, high-numerical aperture objective. Leica LASX software (v. 3.5) was then used to stitch these component volumes into a single image volume of the entire sample. The scanner zoom and focus motor control were set to provide voxel dimensions of 0.5 x 0.5 um laterally and 1 um axially. 2D Immunofluorescence images and data were captured using Nikon EZ-C1 (3.91) confocal system and images produced using NIS-elements software (BR3.2 64 bit).

Data analysis

Code to reproduce figures are available to download from github.com/KPMP/Cell-State-Atlas-2022.

snCv3 and scCv3 sample demultiplexing, barcode processing, and gene expression quantifications were performed with the 10X Cell Ranger v3 pipeline using the GRCh38 (hg38) or GRCh37 (hg19, indicated in Comments column of Supplementary Table 1)) reference genome. For single nucleus data, introns were also included in the expression estimates. SNARE2 data processing pipeline (snarePIP v1.0.1) is available at github.com/huqiwen0313/snarePip. For SNARE2 RNA processing, this involved removal of AC contaminating reads using cutadapt (version 3.1), dropEst (version 0.8.6) to extract cell barcodes and STAR (version 2.5.2b) to align tagged reads to the genome (GRCh38). For SNARE2 AC data, this involved snaptools (version v1.2.3) and minimap (version 2-2.20) for alignment to the genome (GRCh38). snCv3 doublets were identified using DoubletDetection software (version 2.4.0). SNARE2 doublets were identified by both DoubletDetection (version 3.0) and Scrublet (github.com/swolock/scrublet, version 0.2.2). Ambient RNA in scCv3 was corrected using SoupX (version 1.5.0). snCv3/scCv3/SNARE analyses involved the following R packages: Seurat (version 4.0.0), Pagoda2 (version 1.0.2), corrplot (version 0.84), Signac (version 1.1.1), MACS (version 3.0.0a6), chromVAR (version 1.12.0), CisTopic (version 0.3.0), Cicero (version 1.8.1), swne (version 0.6.20), ggdendro (version 0.1.20), circlize (version 0.4.12), g-chromVAR (version 0.3.2), Slingshot (version 2.0.0), WGCNA package (version 1.70-3), Cacao (version 0.2.0), SCCAF (version 0.0.10), scIB (version 1.0.3), DoRothEA (version 1.7.2), viper (version 3.15), CellAlign (https://github.com/shenorrLab/cellAlign), velocyto (version 0.6), CellChat (version 1.0.0). The following python packages were also used: NSForest (version 2.0), velocyto (version 0.17.17), scVelo (version 0.2.4), Celloracle (version 0.9.1), CausalDB database (github.com/mulinlab/CAUSALdb-finemapping-pip). Additional

code for analysis of chromatin data is provided at github.com/yanwu2014/chromfunks.

Slide-seq2 demultiplexing, genome alignment and spatial matching was performed using Slide-seq tools github.com/MacoskoLab/slideseq-tools/releases/tag/0.1. Slide-seq analysis was performed using: Giotto (version 1.0.3), RCTD (version 1.2.0), ggGally (version 2.1.2) and Seurat (version 4.0.0). 10X visium expression analysis, mapping, counting, and clustering was performed using Space Ranger (version 1.0.0) and final data processing was done in Seurat (version 3.2.0 and 3.2.3). Tissue cytometry and analysis were conducted using the Volumetric Tissue Exploration and Analysis (VTEA) software (version 1.0a-r9, www.github.com/icbm-iupui/volumetric-tissue-exploration-analysis) and RStudio (version 1.4) with R (version 4.0.2), corrplot (version 0.84), igraph (version 1.2.6), FNN (version 1.1.3), circlize (version 0.4.12), Hmisc (version 4.5.0), corrplot (version 0.84) and Rtsne (version 0.15).

For manuscripts utilizing custom algorithms or software that are central to the research but not yet described in published literature, software must be made available to editors and reviewers. We strongly encourage code deposition in a community repository (e.g. GitHub). See the Nature Portfolio guidelines for submitting code & software for further information.

Data

Policy information about availability of data

All manuscripts must include a data availability statement. This statement should provide the following information, where applicable:

- Accession codes, unique identifiers, or web links for publicly available datasets
- A description of any restrictions on data availability
- For clinical datasets or third party data, please ensure that the statement adheres to our policy

Processed data, interactive and visualization tools: The snCv3, scCv3, SNARE2, Slide-seq and Visium processed data files are all available for download from GEO (Superseries GSE183279). snCv3 healthy reference data is available for reference-based single cell mapping by the Azimuth tool: https://azimuth.hubmapconsortium.org/. All snCv3 and scCv3 processed data can be accessed and viewed at cellxgene (https://cellxgene.cziscience.com/collections/bcb61471-2a44-4d00-a0af-ff085512674c). snCv3 (excluding COVID-AKI and CKD nephrectomy samples), scCv3, Visium (KPMP biopsies) and 3D imaging can all be visualized and interrogated using the KPMP Data Atlas Explorer: https://atlas.kpmp.org/explorer/. For 3D imaging, the cytometry, cell classifications, gates and neighborhood analysis data are located at: https://doi.org/10.5281/zenodo.7120941.

Raw sequencing and imaging data: Raw sequencing data are under controlled access (human data) as they are potentially identifiable and can be accessed from the respective sources indicated below (summarized in Supplementary Table 1). Raw and processed sequencing and imaging data (snCv3, scCv3, 3D imaging, Slide-seq, Visium) generated as part of the Kidney Precision Medicine Project (KPMP) has been deposited at https://atlas.kpmp.org/repository/ and compiled at https://doi.org/10.48698/3z31-8924. Raw sequencing data can be requested and are available by signing a data use agreement with KPMP. Raw sequencing data (snCv3, SNARE2, Slide-seq) generated as part of the Human Biomolecular Atlas Project (HuBMAP) has been deposited at https://portal.hubmapconsortium.org/ and compiled at https://doi.org/10.35079/hbm776.rgsw.867. The HuBMAP raw data are available for download from the database of Genotypes and Phenotypes (dbGaP, phs002249). snCv3 data not deposited to KPMP or HuBMAP are available from GEO (GSE183279) or, for Covid AKI raw sequencing files, upon request from WU KTRC (sanjayjain@wustl.edu) due to patient confidentiality.

Additional published/public data sets: The following publicly available RNA-seq data sets were used in this study: mouse kidney single-cell (GEO, GSE129798); mouse kidney injury single-nucleus (GEO, GSE139107); human fibroblast and myofibroblast single-cell (Zenodo, 10.5281/zenodo.4059315); mouse distal nephron single-cell and bulk distal segment (GEO, GSE150338); human kidney mature immune single-cell (https://kidney-atlas.cells.ucsc.edu); and human kidney single-nucleus (GEO, GSE151302; https://human-kidney-atac.cells.ucsc.edu). GWAS summary statistics were from the CKDGen Consortium (all eGFR, https://ckdgen.imbi.uni-freiburg.de/files/Wuttke2019), EBI GWAS Catalog (hypertension, https://www.ebi.ac.uk/gwas/efotraits/EFO_0000537), and the CausalDB database (Release 1.1 2019-09-29, http://www.mulinlab.org/causaldb). NEPTUNE sequencing and clinical data were obtained from the Nephrotic Syndrome Study Network and are available upon request to NEPTUNE-STUDY@umich.edu due to patient confidentiality. ERCB data was obtained from GEO (GSE104954). Raw sequencing data (scCv3) on living donor biopsies as part of the Chan Zuckerberg Initiative (CZI) and Human Cell Atlas (HCA) are available from GEO (GSE169285). Additional visium spatial transcriptomic data not in the KPMP repository are available from GEO (GSE171406).

Figures: Source data are provided with this paper. Additional figures can be accessed at Zenodo https://doi.org/10.5281/zenodo.6987337. Schemata of the human nephron and renal corpuscle were developed by the Kidney Precision Medicine Project and HuBMAP (https://doi.org/10.48698/DEM4-0Q93).

Field-specific reporting

Please select the one below that is the best fit for your research. If you are not sure, read the appropriate sections before making your selection.

☒ Life sciences ☐ Behavioural & social sciences ☐ Ecological, evolutionary & environmental sciences

For a reference copy of the document with all sections, see nature.com/documents/nr-reporting-summary-flat.pdf

Life sciences study design

All studies must disclose on these points even when the disclosure is negative.

Sample size	Sample sizes were not predetermined by statistical methods due to nature of this study. The strength lies in the number of individuals analyzed, technologies represented for orthogonal validation and cells analyzed (more than any existing study for the kidney). For snCv3 (n = 36), scCv3 (n = 45), SNARE2 (n = 7), 3D imaging (n = 15), 10X Visium (n = 22) and Slide-seq (n = 6) single nuclei, single cells or tissue sections were obtained from living or deceased donor tissues ("n" here refers to individuals, the number of independent samples is explained in detail in the "Replication" section below). These were obtained from healthy reference, AKI or CKD individuals. To ensure robust cell state profiles, reference tissues were obtained from multiple sources, and biopsies were collected from AKI and CKD patients under rigorous quality assurance and control procedures. This ensured that cell type clusters were not driven by technical artifacts and that our analyses showed rigor and reproducibility.

Data exclusions	Low quality cells or nuclei were excluded from analyses based on established quality filtering metrics:<br><br>snCv3:<br>CellRanger Empty barcode filter<br>Doublets identified using DoubletDetection software<br>< 400 or > 7500 genes detected per cell<br>Gene/UMI ratio filter (Pagoda2)<br><br>scCv3:<br>> 50% mitochondrial reads<br>< 500 or > 5000 genes detected per cell<br><br>SNARE2 - RNA:<br>DropEst cell score < 0.9<br>Doublets identified using DoubletDetection and Scrublet software<br>< 200 or > 7500 genes detected per cell<br>Gene/UMI ratio filter (Pagoda2)<br><br>SNARE2 - AC:<br>Cell barcodes not passing RNA QC filters<br>< 0.15 tss enrichment<br>< 1000 read fragments or 500 UMI per cell<br>< 0.15 of read fragments overlapping promoter regions<br>samples showing < 500 dual omic cells after quality filtering<br>Gene/UMI ratio filter (Pagoda2)<br><br>Visium 10x:<br>In each Visium sample, spots were eliminated if they did not overly tissue. In addition, the outermost layer of spots was eliminated from comparative analyses if the edge was manually cut by a razor.
Replication	RNA-Seq: snCv3 data was generated from 44 independent samples or experiments to cover 36 individuals, scCv3 was generated from 49 samples covering 45 individuals, and SNARE2 was generated from 17 samples covering 7 individuals. snCv3 clustering analysis was performed at multiple k values and cluster assignments were performed using a defined process (see Methods). Reproducibility of assigned cell type annotations was evident from consistent aligned populations found across technologies (scCv3, SNARE, Slide-seq, Visium) and high correlation values with reference (published) data sets.<br><br>Imaging: For 3D imaging and immunofluorescence staining experiments, each staining was repeated on at least 2 separate individuals or separate regions. For ISH, each stain was performed on 6 separate individuals. For Visium spatial transcriptomics, 23 samples from 22 individuals were included in the analysis. These included at least 6 samples from each of the reference, CKD, and AKI categories. For Slide-seq we generated 31 cortical and 36 medullary pucks from 6 individuals. For immunofluorescence validation studies, commercially available antibodies were used; the immunostaining included tissue from patients not contributing to omics data. Similarly, orthogonal validation of omics annotations and spatial localization in Visium studies also included more than four samples each from reference and disease biopsies that were not used to generate single cell gene expression data. This heterogeneity in sampling demonstrated the reproducibility and rigor of the atlas. All attempts at replication were successful for these imaging experiments.<br><br>Further, several technologies were performed on samples from the same individual and in some cases the same tissue block was used to generate multimodal data.
Randomization	Randomization was not used as it was not relevant for this study design as healthy and disease samples were obtained as available. Generation of data and processed files were agnostic to the disease conditions. Batch effects were corrected by scaling expression of each gene to the dataset-wide average and shown to have minimal effect from cell type or cluster contribution plots.
Blinding	All human specimens used in this study were de-identified, however select attributes (condition, age, sex) were available to all investigators. A majority of the analyses were not performed blind as these sample attributes were needed for accurate annotation of cell types or states and for the design of downstream analyses to create maps.

Behavioural & social sciences study design

All studies must disclose on these points even when the disclosure is negative.

Study description	*Briefly describe the study type including whether data are quantitative, qualitative, or mixed-methods (e.g. qualitative cross-sectional, quantitative experimental, mixed-methods case study).*
Research sample	*State the research sample (e.g. Harvard university undergraduates, villagers in rural India) and provide relevant demographic information (e.g. age, sex) and indicate whether the sample is representative. Provide a rationale for the study sample chosen. For studies involving existing datasets, please describe the dataset and source.*
Sampling strategy	*Describe the sampling procedure (e.g. random, snowball, stratified, convenience). Describe the statistical methods that were used to predetermine sample size OR if no sample-size calculation was performed, describe how sample sizes were chosen and provide a rationale for why these sample sizes are sufficient. For qualitative data, please indicate whether data saturation was considered, and what criteria were used to decide that no further sampling was needed.*
Data collection	*Provide details about the data collection procedure, including the instruments or devices used to record the data (e.g. pen and paper,*

Data collection	*computer, eye tracker, video or audio equipment) whether anyone was present besides the participant(s) and the researcher, and whether the researcher was blind to experimental condition and/or the study hypothesis during data collection.*
Timing	*Indicate the start and stop dates of data collection. If there is a gap between collection periods, state the dates for each sample cohort.*
Data exclusions	*If no data were excluded from the analyses, state so OR if data were excluded, provide the exact number of exclusions and the rationale behind them, indicating whether exclusion criteria were pre-established.*
Non-participation	*State how many participants dropped out/declined participation and the reason(s) given OR provide response rate OR state that no participants dropped out/declined participation.*
Randomization	*If participants were not allocated into experimental groups, state so OR describe how participants were allocated to groups, and if allocation was not random, describe how covariates were controlled.*

Ecological, evolutionary & environmental sciences study design

All studies must disclose on these points even when the disclosure is negative.

Study description	*Briefly describe the study. For quantitative data include treatment factors and interactions, design structure (e.g. factorial, nested, hierarchical), nature and number of experimental units and replicates.*
Research sample	*Describe the research sample (e.g. a group of tagged Passer domesticus, all Stenocereus thurberi within Organ Pipe Cactus National Monument), and provide a rationale for the sample choice. When relevant, describe the organism taxa, source, sex, age range and any manipulations. State what population the sample is meant to represent when applicable. For studies involving existing datasets, describe the data and its source.*
Sampling strategy	*Note the sampling procedure. Describe the statistical methods that were used to predetermine sample size OR if no sample-size calculation was performed, describe how sample sizes were chosen and provide a rationale for why these sample sizes are sufficient.*
Data collection	*Describe the data collection procedure, including who recorded the data and how.*
Timing and spatial scale	*Indicate the start and stop dates of data collection, noting the frequency and periodicity of sampling and providing a rationale for these choices. If there is a gap between collection periods, state the dates for each sample cohort. Specify the spatial scale from which the data are taken*
Data exclusions	*If no data were excluded from the analyses, state so OR if data were excluded, describe the exclusions and the rationale behind them, indicating whether exclusion criteria were pre-established.*
Reproducibility	*Describe the measures taken to verify the reproducibility of experimental findings. For each experiment, note whether any attempts to repeat the experiment failed OR state that all attempts to repeat the experiment were successful.*
Randomization	*Describe how samples/organisms/participants were allocated into groups. If allocation was not random, describe how covariates were controlled. If this is not relevant to your study, explain why.*
Blinding	*Describe the extent of blinding used during data acquisition and analysis. If blinding was not possible, describe why OR explain why blinding was not relevant to your study.*

Did the study involve field work? ☐ Yes ☒ No

Field work, collection and transport

Field conditions	*Describe the study conditions for field work, providing relevant parameters (e.g. temperature, rainfall).*
Location	*State the location of the sampling or experiment, providing relevant parameters (e.g. latitude and longitude, elevation, water depth).*
Access & import/export	*Describe the efforts you have made to access habitats and to collect and import/export your samples in a responsible manner and in compliance with local, national and international laws, noting any permits that were obtained (give the name of the issuing authority, the date of issue, and any identifying information).*
Disturbance	*Describe any disturbance caused by the study and how it was minimized.*

Reporting for specific materials, systems and methods

We require information from authors about some types of materials, experimental systems and methods used in many studies. Here, indicate whether each material, system or method listed is relevant to your study. If you are not sure if a list item applies to your research, read the appropriate section before selecting a response.

Materials & experimental systems

n/a	Involved in the study
☐	☒ Antibodies
☒	☐ Eukaryotic cell lines
☒	☐ Palaeontology and archaeology
☒	☐ Animals and other organisms
☐	☒ Human research participants
☐	☒ Clinical data
☒	☐ Dual use research of concern

Methods

n/a	Involved in the study
☒	☐ ChIP-seq
☒	☐ Flow cytometry
☒	☐ MRI-based neuroimaging

Antibodies

Antibodies used

The antibodies used and associated details are tabulated in Supplemental tables 35 and 36.
For 3D cytometry studies
Primary antibody or Fluorescent probe, Target, Vendor, Dilution, Secondary antibody, Vendor, Dilution
Goat anti-aquaporin1(AQP1), Proximal tubules, Santa Cruz (sc-9878), 1:50, (please note, very minimal content about this discontinued antibody)
Alexa568 donkey anti-goat, ThermoFisher(A-11057), 1:200
Rabbit anti-myeloperoxidase(MPO), Neutrophils, Abcam (ab9535), 1:50
DyLight 594 donkey anti-rabbit, ThermoFisher(SA5-10040), 1:200
Mouse anti-CD68, Macrophages, Dako (M0876), 1:50, Alexa 633donkey anti-mouse, ThermoFisher(A16019)(Conjugated in-house with ThermoFisher (A20170), 1:200
Mouse Alexa660 anti-SIGLEC8, Eosinophils, Biolegend (347102)(Conjugated in-house with ThermoFisher (A20171)), 1:50
Sheep Alexa546 anti-Uromodulin(UMOD), Thick ascending limb, R&D Systems (AF5144), 1:200, conjugated in house with kit (https://www.thermofisher.com/order/catalog/product/A20183)
Mouse Alexa647 anti-CD3, T-cells, BD Pharmingen (557706), 1:50
DAPI, Nuclei, ThermoFisher (D1306), 1:100
Oregon Green Phalloidin, Filamentous actin (vasculature, brush border),ThermoFisher O7466, 1:200

For 2D confocal immunofluorescence microscopy
Primary Antibody, Against Raised in, Company Cat #, Primary antibody dilution, secondary Antibody, secondary Antibody dilution,
Uromodulin human mouse Ray biotech 119-13298 1:100 Goat anti-mouse alexa-488 1:400
CD133 (PROM1) human, mouse, rat Rabbit ThermoFisher PA5-38014 1:50 goat anti-ratbbit-cy3 1:400
KIM1 human Rabbit ThermoFisher PA5-79345 1:250 goat anti-rabbit -cy3 1:400
VCAM1 human, rat mouse ThermoFisher MA5-11447 1:50 Goat anti-mouse alexa-488 1:400
AQP1 human Rabbit santa cruz sc-20810 1:100 goat anti-rabbit -cy3 1:400

Validation

Validation of antibodies and confidence in their staining is derived from several sets of data including vendors specifications, omitting primary antibody, well-established expected cell-type staining pattern for the indicated antibodies in the literature, referring to human protein atlas data where available and orthogonal validations in the multiomics data presented.
3D IF antibodies:
AQP1 - https://www.scbt.com/p/aqp1-antibody-l-19
AF-568 - https://www.thermofisher.com/antibody/product/Donkey-anti-Goat-IgG-H-L-Cross-Adsorbed-Secondary-Antibody-Polyclonal/A-11057
MPO - https://www.abcam.com/myeloperoxidase-antibody-ab9535.html
DyLight-594 - https://www.thermofisher.com/antibody/product/Donkey-anti-Rabbit-IgG-H-L-Cross-Adsorbed-Secondary-Antibody-Polyclonal/SA5-10040
CD68 - https://www.agilent.com/en/product/immunohistochemistry/antibodies-controls/primary-antibodies/cd68-%28concentrate%29-76550
Conjugated in-house to AF-633 – kit # https://www.thermofisher.com/order/catalog/product/A20170
SIGLEC8 (AF660) - https://www.biolegend.com/de-at/products/purified-anti-human-siglec-8-antibody-6383
Conjugated in-house to AF-660 – kit # https://www.thermofisher.com/order/catalog/product/A20171
UMOD - https://www.rndsystems.com/products/human-uromodulin-antibody_af5144
Conjugated in-house to AF-546 – kit # https://www.thermofisher.com/order/catalog/product/A20183 (https://www.ncbi.nlm.nih.gov/pmc/articles/PMC8363780/)
CD3 - https://www.bdbiosciences.com/en-us/products/reagents/flow-cytometry-reagents/research-reagents/single-color-antibodies-ruo/alexa-fluor-647-mouse-anti-human-cd3.557706
DAPI - https://www.thermofisher.com/order/catalog/product/D21490
Phalloidin - https://www.thermofisher.com/order/catalog/product/O7466

2D Antibodies:
Uromodulin - https://www.raybiotech.com/mouse-anti-human-uromodulin/
CD133 (PROM1) - https://www.thermofisher.com/antibody/product/CD133-Antibody-Polyclonal/PA5-38014
KIM1 - https://www.thermofisher.com/antibody/product/KIM-1-Antibody-Polyclonal/PA5-79345
VCAM1 - https://www.thermofisher.com/antibody/product/VCAM-1-Antibody-clone-1-4C3-Monoclonal/MA5-11447
AQP1 - https://www.citeab.com/antibodies/789773-sc-20810-aqp1-antibody-h-55

Eukaryotic cell lines

Policy information about cell lines

Cell line source(s)	*State the source of each cell line used.*
Authentication	*Describe the authentication procedures for each cell line used OR declare that none of the cell lines used were authenticated.*
Mycoplasma contamination	*Confirm that all cell lines tested negative for mycoplasma contamination OR describe the results of the testing for mycoplasma contamination OR declare that the cell lines were not tested for mycoplasma contamination.*
Commonly misidentified lines (See ICLAC register)	*Name any commonly misidentified cell lines used in the study and provide a rationale for their use.*

Palaeontology and Archaeology

Specimen provenance	*Provide provenance information for specimens and describe permits that were obtained for the work (including the name of the issuing authority, the date of issue, and any identifying information). Permits should encompass collection and, where applicable, export.*
Specimen deposition	*Indicate where the specimens have been deposited to permit free access by other researchers.*
Dating methods	*If new dates are provided, describe how they were obtained (e.g. collection, storage, sample pretreatment and measurement), where they were obtained (i.e. lab name), the calibration program and the protocol for quality assurance OR state that no new dates are provided.*

☐ Tick this box to confirm that the raw and calibrated dates are available in the paper or in Supplementary Information.

Ethics oversight	*Identify the organization(s) that approved or provided guidance on the study protocol, OR state that no ethical approval or guidance was required and explain why not.*

Note that full information on the approval of the study protocol must also be provided in the manuscript.

Animals and other organisms

Policy information about studies involving animals; ARRIVE guidelines recommended for reporting animal research

Laboratory animals	*For laboratory animals, report species, strain, sex and age OR state that the study did not involve laboratory animals.*
Wild animals	*Provide details on animals observed in or captured in the field; report species, sex and age where possible. Describe how animals were caught and transported and what happened to captive animals after the study (if killed, explain why and describe method; if released, say where and when) OR state that the study did not involve wild animals.*
Field-collected samples	*For laboratory work with field-collected samples, describe all relevant parameters such as housing, maintenance, temperature, photoperiod and end-of-experiment protocol OR state that the study did not involve samples collected from the field.*
Ethics oversight	*Identify the organization(s) that approved or provided guidance on the study protocol, OR state that no ethical approval or guidance was required and explain why not.*

Note that full information on the approval of the study protocol must also be provided in the manuscript.

Human research participants

Policy information about studies involving human research participants

Population characteristics	The population used here were adults in the age interval 20-80 and included both sexes and participants of different races. The associated clinical metadata includes age, sex, race, comorbidities, eGFR, certain medications and is detailed in supplemental table 3. The clinical conditions include AKI and CKD.
Recruitment	Participants were recruited from different sites and IRB approval was obtained for use of tissue and data for research in a deidentifiable manner. To obtain consent, the coordinators would approach the participant after consultations with the clinical team, go over the study with them, address any questions and concerns. Once consent was obtained, samples are procured and preserved in a timely manner using standardized protocols that have been published and available on KPMP.org. Recruitment of AKI and CKD patients were per established clinical criteria (https://www.kpmp.org/for-clinicians). The reference tissue samples were selected from patients with normal kidney function and/or age appropriate histopathology as they became available. Samples under waived consent are described in the ethics statement. The associated clinical and pathological data is provided in Supplemental Table3 for readers to interpret the study results.
Ethics oversight	We have complied with all ethical regulations related to this study. Human samples (Supplementary Table 1) collected as part of the Kidney Precision Medicine Project (KPMP) consortium (KPMP.org) were obtained with informed consent and approved under a protocol by the KPMP single IRB of the University of Washington Institutional Review Board (IRB#20190213). Samples as part of the Human Biomolecular Atlas Program (HuBMAP) consortium were collected by the Kidney Translational Research Center (KTRC) under a protocol approved by the Washington University Institutional Review Board (IRB #201102312).

Informed consent was obtained for the use of data and samples for all participants at Washington University, including living patients undergoing partial or total nephrectomy or from discarded deceased kidney donors. Cortical and papillary biopsy samples from patients with stone disease were obtained with informed consent from Indiana University and approved by the Indiana University Institutional Review Board (IRB #1010002261). For Visium Spatial Gene Expression, reference nephrectomies and kidney biopsy specimens were obtained from the KPMP under informed consent or the Biopsy Biobank Cohort of Indiana (BBCI)49 under waived consent as approved by the Indiana University Institutional Review Board (IRB # 1906572234). Living donor biopsies as part of the Human Cell Atlas (HCA) were obtained with informed consent under the Human Kidney Transplant Transcriptomic Atlas (HKTTA) under IRB HUM00150968. Deidentified leftover frozen COVID-19 AKI kidney biopsies were obtained from the Johns Hopkins University pathology archive under waived consent approved by the Johns Hopkins Institutional Review Board (IRB 00090103).

Note that full information on the approval of the study protocol must also be provided in the manuscript.

Clinical data

Policy information about clinical studies

All manuscripts should comply with the ICMJE guidelines for publication of clinical research and a completed CONSORT checklist must be included with all submissions.

Clinical trial registration	NOT APPLICABLE
Study protocol	*Note where the full trial protocol can be accessed OR if not available, explain why.*
Data collection	*Describe the settings and locales of data collection, noting the time periods of recruitment and data collection.*
Outcomes	*Describe how you pre-defined primary and secondary outcome measures and how you assessed these measures.*

Dual use research of concern

Policy information about dual use research of concern

Hazards

Could the accidental, deliberate or reckless misuse of agents or technologies generated in the work, or the application of information presented in the manuscript, pose a threat to:

No	Yes	
☒	☐	Public health
☒	☐	National security
☒	☐	Crops and/or livestock
☒	☐	Ecosystems
☒	☐	Any other significant area

Experiments of concern

Does the work involve any of these experiments of concern:

No	Yes	
☒	☐	Demonstrate how to render a vaccine ineffective
☒	☐	Confer resistance to therapeutically useful antibiotics or antiviral agents
☒	☐	Enhance the virulence of a pathogen or render a nonpathogen virulent
☒	☐	Increase transmissibility of a pathogen
☒	☐	Alter the host range of a pathogen
☒	☐	Enable evasion of diagnostic/detection modalities
☒	☐	Enable the weaponization of a biological agent or toxin
☒	☐	Any other potentially harmful combination of experiments and agents

ChIP-seq

Data deposition

☐ Confirm that both raw and final processed data have been deposited in a public database such as GEO.

☐ Confirm that you have deposited or provided access to graph files (e.g. BED files) for the called peaks.

Data access links<br>*May remain private before publication.*	*For "Initial submission" or "Revised version" documents, provide reviewer access links. For your "Final submission" document, provide a link to the deposited data.*

Files in database submission	*Provide a list of all files available in the database submission.*
Genome browser session (e.g. UCSC)	*Provide a link to an anonymized genome browser session for "Initial submission" and "Revised version" documents only, to enable peer review. Write "no longer applicable" for "Final submission" documents.*

Methodology

Replicates	*Describe the experimental replicates, specifying number, type and replicate agreement.*
Sequencing depth	*Describe the sequencing depth for each experiment, providing the total number of reads, uniquely mapped reads, length of reads and whether they were paired- or single-end.*
Antibodies	*Describe the antibodies used for the ChIP-seq experiments; as applicable, provide supplier name, catalog number, clone name, and lot number.*
Peak calling parameters	*Specify the command line program and parameters used for read mapping and peak calling, including the ChIP, control and index files used.*
Data quality	*Describe the methods used to ensure data quality in full detail, including how many peaks are at FDR 5% and above 5-fold enrichment.*
Software	*Describe the software used to collect and analyze the ChIP-seq data. For custom code that has been deposited into a community repository, provide accession details.*

Flow Cytometry

Plots

Confirm that:

- ☐ The axis labels state the marker and fluorochrome used (e.g. CD4-FITC).
- ☐ The axis scales are clearly visible. Include numbers along axes only for bottom left plot of group (a 'group' is an analysis of identical markers).
- ☐ All plots are contour plots with outliers or pseudocolor plots.
- ☐ A numerical value for number of cells or percentage (with statistics) is provided.

Methodology

Sample preparation	*Describe the sample preparation, detailing the biological source of the cells and any tissue processing steps used.*
Instrument	*Identify the instrument used for data collection, specifying make and model number.*
Software	*Describe the software used to collect and analyze the flow cytometry data. For custom code that has been deposited into a community repository, provide accession details.*
Cell population abundance	*Describe the abundance of the relevant cell populations within post-sort fractions, providing details on the purity of the samples and how it was determined.*
Gating strategy	*Describe the gating strategy used for all relevant experiments, specifying the preliminary FSC/SSC gates of the starting cell population, indicating where boundaries between "positive" and "negative" staining cell populations are defined.*

☐ Tick this box to confirm that a figure exemplifying the gating strategy is provided in the Supplementary Information.

Magnetic resonance imaging

Experimental design

Design type	*Indicate task or resting state; event-related or block design.*
Design specifications	*Specify the number of blocks, trials or experimental units per session and/or subject, and specify the length of each trial or block (if trials are blocked) and interval between trials.*
Behavioral performance measures	*State number and/or type of variables recorded (e.g. correct button press, response time) and what statistics were used to establish that the subjects were performing the task as expected (e.g. mean, range, and/or standard deviation across subjects).*

Acquisition

Imaging type(s) | Specify: functional, structural, diffusion, perfusion.

Field strength | Specify in Tesla

Sequence & imaging parameters | Specify the pulse sequence type (gradient echo, spin echo, etc.), imaging type (EPI, spiral, etc.), field of view, matrix size, slice thickness, orientation and TE/TR/flip angle.

Area of acquisition | State whether a whole brain scan was used OR define the area of acquisition, describing how the region was determined.

Diffusion MRI ☐ Used ☐ Not used

Preprocessing

Preprocessing software | Provide detail on software version and revision number and on specific parameters (model/functions, brain extraction, segmentation, smoothing kernel size, etc.).

Normalization | If data were normalized/standardized, describe the approach(es): specify linear or non-linear and define image types used for transformation OR indicate that data were not normalized and explain rationale for lack of normalization.

Normalization template | Describe the template used for normalization/transformation, specifying subject space or group standardized space (e.g. original Talairach, MNI305, ICBM152) OR indicate that the data were not normalized.

Noise and artifact removal | Describe your procedure(s) for artifact and structured noise removal, specifying motion parameters, tissue signals and physiological signals (heart rate, respiration).

Volume censoring | Define your software and/or method and criteria for volume censoring, and state the extent of such censoring.

Statistical modeling & inference

Model type and settings | Specify type (mass univariate, multivariate, RSA, predictive, etc.) and describe essential details of the model at the first and second levels (e.g. fixed, random or mixed effects; drift or auto-correlation).

Effect(s) tested | Define precise effect in terms of the task or stimulus conditions instead of psychological concepts and indicate whether ANOVA or factorial designs were used.

Specify type of analysis: ☐ Whole brain ☐ ROI-based ☐ Both

Statistic type for inference (See Eklund et al. 2016) | Specify voxel-wise or cluster-wise and report all relevant parameters for cluster-wise methods.

Correction | Describe the type of correction and how it is obtained for multiple comparisons (e.g. FWE, FDR, permutation or Monte Carlo).

Models & analysis

n/a	Involved in the study
☐	☐ Functional and/or effective connectivity
☐	☐ Graph analysis
☐	☐ Multivariate modeling or predictive analysis

Functional and/or effective connectivity | Report the measures of dependence used and the model details (e.g. Pearson correlation, partial correlation, mutual information).

Graph analysis | Report the dependent variable and connectivity measure, specifying weighted graph or binarized graph, subject- or group-level, and the global and/or node summaries used (e.g. clustering coefficient, efficiency, etc.).

Multivariate modeling and predictive analysis | Specify independent variables, features extraction and dimension reduction, model, training and evaluation metrics.

[illegible signature]
10-22-22
